# Anatomical phenotyping and staging of brain arteriovenous malformations

**DOI:** 10.1093/braincomms/fcag039

**Published:** 2026-02-08

**Authors:** Benjamin Beyersdorf, Yannis Schwieger, Luis Padevit, Zsolt Kulcsar, Menno R Germans, Luca Regli, Kevin Akeret

**Affiliations:** Department of Neurosurgery, Clinical Neuroscience Centre, University Hospital Zurich and University of Zurich, 8091 Zurich, Switzerland; Department of Surgery, Stadtspital Triemli, 8063 Zurich, Switzerland; Department of Neurosurgery, Clinical Neuroscience Centre, University Hospital Zurich and University of Zurich, 8091 Zurich, Switzerland; Department of Neuroradiology, Clinical Neuroscience Centre, University Hospital Zurich and University of Zurich, 8091 Zurich, Switzerland; Department of Neurosurgery, Clinical Neuroscience Centre, University Hospital Zurich and University of Zurich, 8091 Zurich, Switzerland; Department of Neurosurgery, Clinical Neuroscience Centre, University Hospital Zurich and University of Zurich, 8091 Zurich, Switzerland; Department of Neurosurgery, Clinical Neuroscience Centre, University Hospital Zurich and University of Zurich, 8091 Zurich, Switzerland

**Keywords:** AVM, haemorrhage, meta-topology, ontogenesis, risk stratification

## Abstract

Brain arteriovenous malformations (AVMs) are potentially life-threatening vascular anomalies that pose significant clinical challenges due to their heterogeneous anatomy and unpredictable natural history. Existing risk stratification models largely rely on isolated imaging markers and fail to account for the dynamic spatial–temporal complexity of AVMs. Building on our prior work, demonstrating how ontogenesis dictates clinical outcomes of neuroepithelial tumours, we hypothesize that AVMs are similarly influenced by developmental processes that define their spatial distribution, vascular architecture and susceptibility to complications. Here, we present the protocol and pilot data of our multicentre, retrospective and prospective observational study, which introduces a multidimensional approach integrating precise anatomical phenotyping with ontogenetic mapping and the analysis of dynamic structural changes over time. By leveraging unsupervised non-negative matrix factorization, we identified six biologically plausible meta-topologies in our single-centre pilot dataset of 416 patients, supporting the feasibility of this approach. We now seek to expand the study into a multicentre effort with both retrospective and prospective enrollment, aiming for a total sample size of ∼1000 patients. This expansion is essential to enhance the granularity, reproducibility and clinical utility of the meta-topologies. Ultimately, the objective of this integrative framework is to facilitate the development of a robust biologically informed risk-stratifying staging system, enhancing personalized treatment strategies and optimizing patient outcomes.

## Teaser

Beyersdorf *et al.* present the protocol and pilot data of a multicentre observational study that integrates precise anatomical phenotyping and dynamic structural features with ontogenetic mapping and the identification of higher-order meta-topologies, to develop a biologically informed staging system for risk stratification and personalized treatment guidance in brain arteriovenous malformations.

## Graphical Abstract

**Graphical Abstract:**
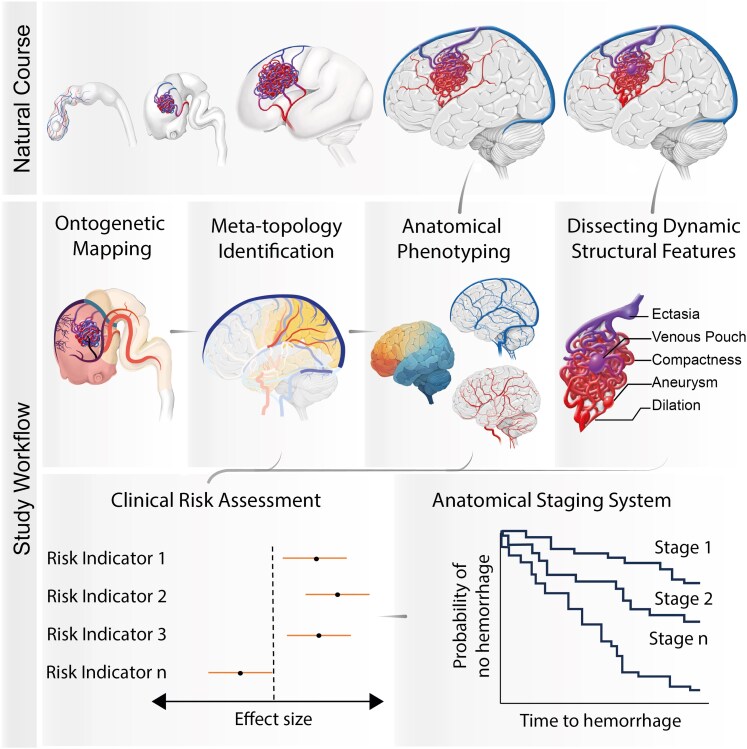

## Introduction

The clinical management of brain arteriovenous malformations (AVMs) remains fundamentally limited by the lack of a comprehensive risk-stratifying framework that integrates their developmental origins, complex spatial architecture and dynamic evolution over time. Defined by abnormal direct connections between arteries and veins,^1,2^ these fragile lesions predispose patients to life-threatening complications including intracranial haemorrhage, seizures and progressive neurological decline, often resulting in long-term disability and a reduced quality of life.^3,4^ The management of AVMs is particularly challenging because treatment options—endovascular embolization, neurosurgical resection or radiosurgery—carry substantial risks of complications, requiring clinicians to balance the natural risk of haemorrhage with the risks of treatment. Although AVMs display substantial biological diversity with variations in their developmental origin, anatomical distribution, nidus compactness, arterial and venous architecture and flow-related adaptations, current clinical approaches reduce this complexity to a narrow set of isolated imaging markers used for haemorrhage risk assessment.^5,6^ While these features have been individually described, the field lacks a unifying framework capable of deciphering how they converge into generalizable anatomical patterns. This fragmented perspective highlights the necessity of an integrated, biologically informed model that synthesizes temporal evolution, spatial anatomy and vascular dynamics to improve the prediction of clinical trajectories and guide personalized treatment strategies.

**Figure 1:**
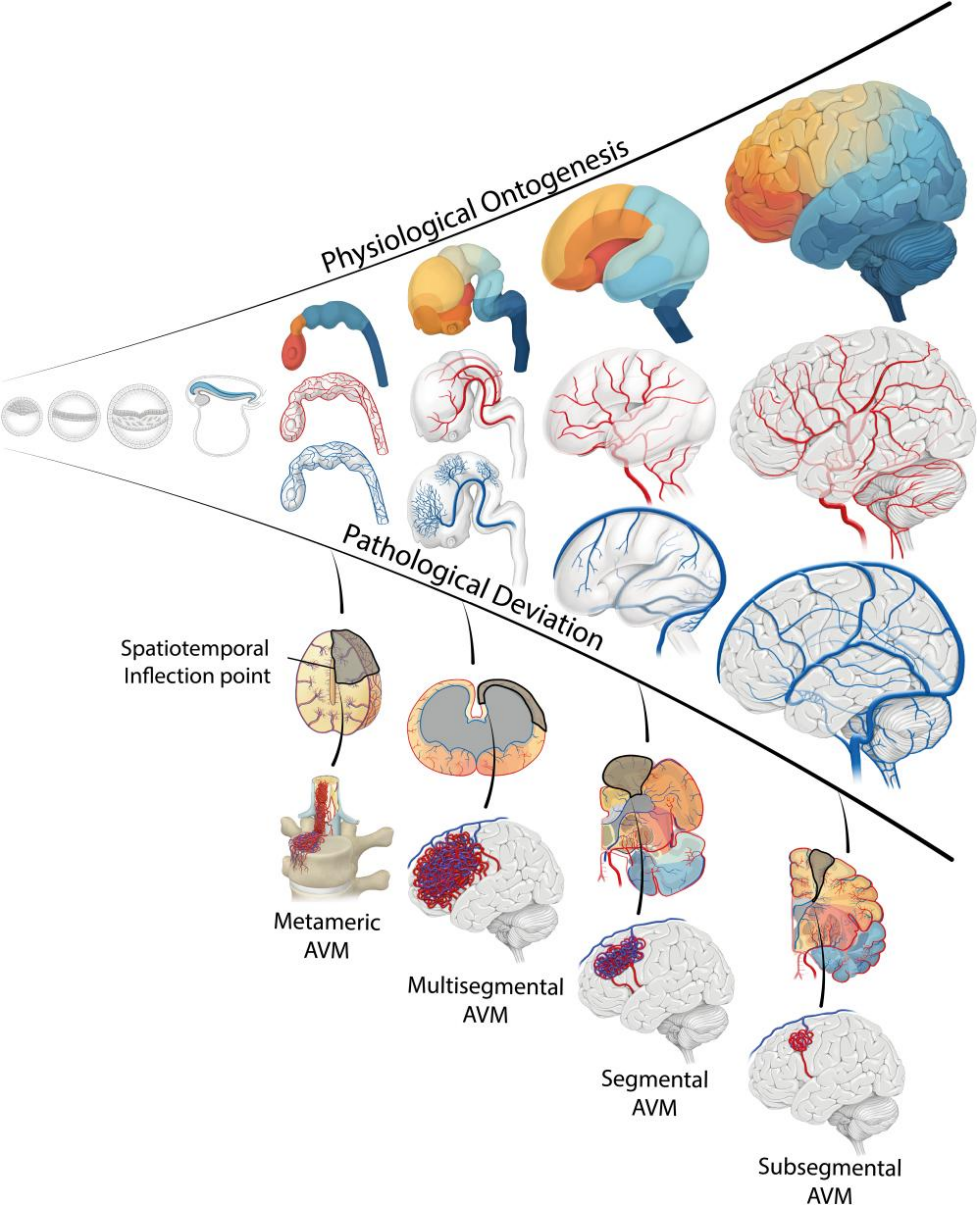
**Spatiotemporal ontogenetic identity in brain arteriovenous malformations.** The upper part of this figure illustrates the physiological ontogenesis from conception to the postnatal brain, depicted for parenchymal, arterial and venous units. It emphasizes different hypothesized ontogenetic inflection points, marking the onset of deviation from normal development, which may influence the AVM’s parenchymal distribution, vascular architecture and clinical behaviour.

Recently, we proposed an ontogenetic approach to the understanding of neuroepithelial tumours by demonstrating how ontogenesis dictates their spatial–temporal organization and clinical outcomes.^7-9^ Building on this concept, we propose a similar developmental framework for AVMs. We hypothesize that AVMs originate at specific ontogenetic inflection points—the critical moment when normal morphogenesis takes a pathological turn and the AVM begins to form (Fig. 1). The spatial and temporal context of this disruption defines a pathologically affected ontogenetic unit, which determines the AVM’s 3D evolution and unique ontogenetic identity. Over time, these lesions may further transition from diffuse, low-flow configurations to compact, high-flow structures through cumulative vessel recruitment and adaptive remodelling. This paradigm recognizes AVMs not as static vascular anomalies but as dynamic, whole-unit pathologies with predictable trajectories—an insight that may transform risk stratification and guide more precise, biologically informed treatment strategies.

**Figure 2:**
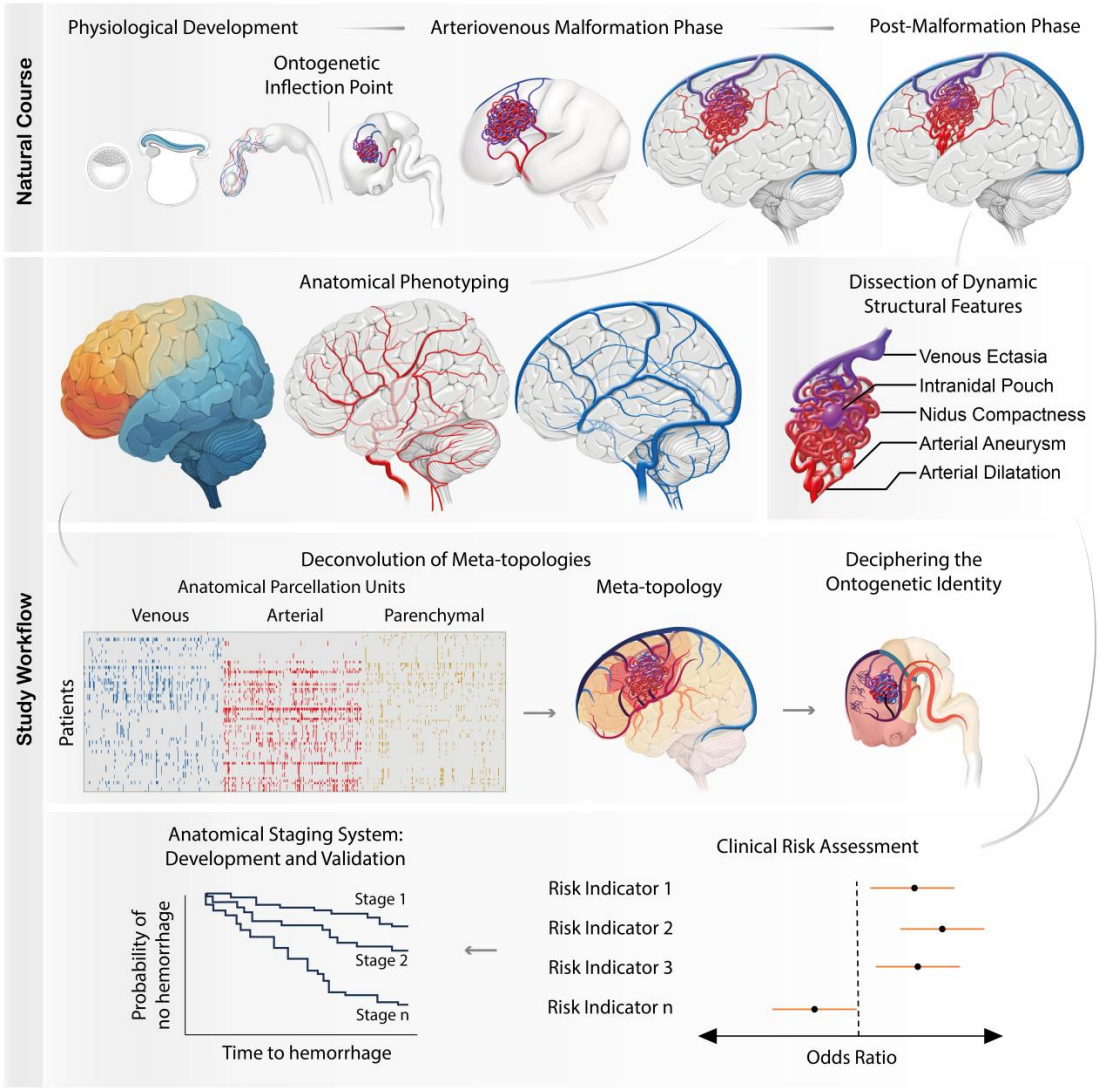
**The multidimensional spatiotemporal approach.** The top row illustrates the natural course of AVM evolution, the lower section outlines the study workflow: 3D anatomical phenotyping of mature AVMs is performed and additional dynamic structural features are dissected. Next, anatomical phenotypes are integrated to identify AVM meta-topologies and decipher their corresponding ontogenetic identity. Clinical risk assessment combines meta-topologies and dynamic structural features to inform clinical risk stratification and support the development of an anatomical staging system.

This multicentre, combined retrospective and prospective observational study aims to develop an integrative anatomical staging system for improved prediction of haemorrhage risk in brain AVMs. We propose a multidimensional framework that follows a sequential analytical approach: first, we perform detailed anatomical phenotyping of AVMs by characterizing their parenchymal, arterial and venous architecture. These anatomical features are then integrated using unsupervised non-negative matrix factorization (NNMF) to identify higher-order AVM meta-topologies that reveal generalizable patterns of AVM topology. In the next step, we apply ontogenetic mapping to trace these patterns back to their embryological origins, uncovering potential ontogenetic vulnerabilities. Finally, we incorporate dynamic structural features such as aneurysms, nidus compactness and venous ectasia, which may influence clinical progression. By combining these elements, this study aims to construct and validate a biologically informed AVM staging system to support personalized risk assessment and guide treatment decisions (Fig. 2).

## Materials and methods

### Hypotheses, objectives and end-points

This study is based on the premise that AVMs are dynamic vascular anomalies, the majority of which arise from disruptions in ontogenesis, with their anatomical architecture and clinical behaviour influenced by both developmental processes and subsequent structural modifications. The overarching goal of this study is to develop a comprehensive AVM staging system that integrates ontogenetic insights, anatomical phenotypes and dynamic structural modifications to reliably stratify risk, predict clinical outcomes and provide a practical framework for personalized treatment planning.

#### Hypotheses

1. Anatomical phenotyping reveals AVM meta-topologies.

AVMs are characterized by a complex parenchymal, arterial and venous anatomy. We hypothesize that there are generalizable anatomical patterns in the inter-structural association within each of these anatomical elements (parenchymal, arterial and venous topology), and their association to each other (AVM meta-topologies).

2. Anatomical AVM phenotypes and meta-topologies have a spatiotemporal ontogenetic identity.

While few AVMs are known to develop *de novo* in adulthood, many are shaped during ontogenesis, with their mature parenchymal, arterial and venous anatomy reflecting pathological processes during development. We hypothesize that AVMs originate at distinct ontogenetic inflection points, when development deviates from its physiological trajectory, imprinting a spatiotemporal ontogenetic identity on AVMs (Fig. 1). The timing and structural context of the inflection point may define the spatial organization of the involved brain parenchyma and the arteriovenous architecture of the malformation. Thus, AVM meta-topologies should mirror the spatial and temporal patterns of ontogeny.

3. An anatomical AVM staging system enhances clinical risk assessment.

Acquired structural changes, such as flow-induced aneurysms and venous ectasia, develop during or after AVM formation and are believed to significantly influence the clinical course of AVMs. We hypothesize that a multidimensional anatomical staging approach, incorporating AVM meta-topology, ontogenetic identity, and dynamic structural features, provide a predictive framework to link AVM anatomy to critical clinical outcomes such as haemorrhage, seizures and neurological decline.

#### Objectives

Because this study is exploratory in nature, the distinction between primary and secondary objectives is not applicable. Instead, the study follows a sequential five-step approach, where each objective builds on the previous steps:

1. Anatomical phenotyping of AVMs.

To assess the topographic prevalence and topographic probability of the parenchymal, arterial and venous anatomy in AVMs.

2. Deconvolution of AVM meta-topologies.

To identify higher-order generalizable patterns in AVMs (meta-topologies) by integrating the topological relationship between parenchymal, arterial and venous anatomical phenotypes.

3. Decipher a spatiotemporal ontogenetic identity in AVMs.

To map anatomical AVM phenotypes and meta-topologies to our current spatiotemporal understanding of parenchymal, arterial and venous ontogeny with the aim to identify distinct inflection points and developmental vulnerabilities.

4. Linking AVM anatomy to clinical outcomes.

To evaluate how AVM meta-topologies, ontogenetic identities and dynamic structural features correlate with critical clinical outcomes such as haemorrhage, seizures and neurological decline.

5. Improve clinical risk stratification through an anatomical staging system.

To develop and validate an anatomical staging system based on the specific association of anatomical features to clinical outcomes, establishing a practical tool for AVM risk stratification and personalized treatment.

#### Primary clinical end-point

- Intracranial haemorrhage

The primary end-point of this study is the occurrence of intracranial haemorrhage in AVM patients, assessed as a binary outcome (presence or absence). Haemorrhagic events often initiate a cascade of further complications, including an increased risk of epilepsy, hydrocephalus, focal neurological deficits (FND) and a decline in overall neurological function.^10^ Thus, haemorrhage was selected as the primary end-point due to its significant impact on morbidity and mortality of patients.

#### Secondary clinical end-points

- Seizure occurrence

The occurrence of seizures will also be recorded as a binary outcome. By specifically focusing on seizures that occur prior to any haemorrhagic event, we aim to evaluate how specific AVM features influence seizure risk independently of haemorrhage.

- Functional outcome

Functional status will be assessed both at initial diagnosis and the last follow-up visit using the modified Rankin Scale (mRS). This end-point aims to elucidate the association of specific AVM characteristics with patient’s neurological function.

#### Exploratory end-points

Additional clinical and radiological parameters, which may provide insights into complication risks and outcomes, will be explored. The collected variables are defined in detail (refer to Supplementary Appendix 1), ensuring transparency and replicability.

### Study population and design

- Target population

All individuals with brain AVMs, representing the broader group for which the study findings aim to be generalized.

- Study population

Patients with confirmed AVM diagnoses who are accessible for inclusion in the study. This group consists of individuals that presented to the participating institutions and who meet the eligibility criteria.

- Pilot sample

The retrospective cohort from the University Hospital Zurich (*n* = 416) represents the pilot sample, used for preliminary data collection and analysis. This cohort is used to assess the feasibility of the study methods, refine data collection protocols and provide early insights into AVM characteristics.

This study follows a staged, multicentre, retrospective and prospective observational design to analyse imaging and clinical data from patients with brain AVMs. This design is particularly suited to the rarity of AVMs, enabling the inclusion of a large, heterogeneous cohort across institutions. The staged approach ensures methodological consistency and scientific rigour through the following phases: (i) Inclusion and analysis of the full retrospective Zurich cohort (*n* = 416) to validate feasibility, refine methodology and estimate effect sizes. (ii) Completion of the registered report to define the detailed analysis plan and guide subsequent data collection. (iii) Multicentre data expansion, combining additional retrospective cases and prospective enrollment, strictly adhering to the protocol defined in this report.

#### Eligibility criteria

Participants fulfilling all of the following ‘inclusion criteria’ are eligible for the study:

- Diagnosis of a brain AVM, as confirmed by MRI or digital subtraction angiography (DSA).
- In retrospective cases: sufficient data available in the hospital records to support clinical characterization. Clinical records should include, at minimum: (i) documentation of the presence or absence of haemorrhage and seizures and (ii) description of the functional status at time of presentation and last follow-up, allowing for the assessment of a mRS score at either timepoint.

Patients will be excluded from the study if they meet any of the following exclusion criteria:

- Lack of MRI data necessary for detailed phenotyping analyses.
- Patients under 18 years of age at time of last follow-up.
- Any documented refusal of the use of health data for research purposes, e.g. rejection of general consent (GC).
- Non-AVM-associated intracranial haemorrhage (e.g. due to other types of vascular malformations or traumatic brain injury) prior to AVM diagnosis.

#### Power analysis

In our pilot analysis of 416 retrospectively included patients from the University Hospital Zurich, we identified an optimal number of six distinct AVM meta-topologies using NNMF, demonstrating both the feasibility and biological plausibility of this approach. However, the resolution of these meta-topologies depends heavily on the number and diversity of cases included. Expanding the sample size is therefore essential to increase the granularity of clustering and to identify more refined subtypes that are not only surgically meaningful (e.g. for defining resection corridors or anticipating operative complexity) but also clinically relevant for risk stratification (e.g. haemorrhage, seizures or functional outcomes).

In one of the most comprehensive works in the literature, Prof Gazi Yaşargil^2^ described a broad spectrum of surgically distinct AVM types, with at least 10–12 anatomical categories based on their location and microsurgical complexity. Based on this, we hypothesize that the identification of 12 meta-topologies may offer sufficient anatomical resolution to support a biologically refined AVM staging system. Given that the pilot data suggest ∼70 patients per meta-topology, a total sample of 840 patients is anticipated. To further enable staging system validation, we plan to allocate an additional 20% of the sample as a dedicated validation cohort, resulting in a final target of 1008 patients. Therefore, the expansion of the cohort to 1008 patients is essential to develop a robust, granular and clinically meaningful AVM staging system.

### Data collection

#### Data sources

Retro- and prospectively collected data will be extracted from the electronic medical records at the University Hospital Zurich and additional external centres. Key data sources include:

- Clinical records: documentation of patient demographics, clinical presentation, treatment details and follow-up outcomes.
- Imaging records: CT, MRI and DSA images in DICOM format.

#### Data collection process

- Eligibility verification: eligible patients will be identified from the electronic medical records, and imaging records will be used to confirm the diagnosis of brain AVMs.
- Data extraction: data will be extracted in a pseudonymized format to maintain patient confidentiality. To ensure transparency and compliance, all data beyond the pilot dataset will be collected and analysed only after the in-principle acceptance of this registered report. Angioarchitectural variables will be assessed via DSA, while parenchymal topography will be characterized through MRI.
- Quality checks: all extracted data will undergo quality checks to ensure completeness and accuracy.
  - First, this includes double-checking imaging interpretations against neuroradiological reports, as neuroradiologists have prospectively characterized AVM parenchymal anatomical location, arterial feeding arteries and venous drainage patterns as part of routine patient care in most cases.
  - Further, two independent raters (B.B. and K.A.) will annotate predefined parenchymal, arterial and venous units as part of the anatomical phenotyping workflow, blinded to clinical outcomes and to each other’s ratings. Disagreements are reviewed by a three-member panel (including at least a board-certified neurosurgeon and board-certified neuroradiologist). As anatomical units are coded in a binary fashion (involved/not involved), inter-rater reliability (IRR) is reported as Cohen’s kappa with 95% confidence intervals (CIs), summarized overall and by compartment (parenchymal/arterial/venous). The phenotyping definitions, de-identified rating datasets and all analysis code used to compute IRR metrics will be made publicly available.
- *Study variables and definitions:* clinical and radiological parameters collected in this study are defined with sufficient detail to ensure the highest standards of transparency and reproducibility (refer to Supplementary Appendix 1). These parameters, including anatomical and morphological features, align with consensus recommendations for AVM research reporting terminology.^11^ Further, parenchymal, arterial and venous units are precisely predefined (refer to Supplementary Appendices 2–4). The delineation of each major artery and their branches as well as each major sinus or vein and their tributaries is based on the anatomical definitions provided by the seminal textbooks ‘Cranial Anatomy and Surgical Approaches’ by Rhoton^12^ and ‘Diagnostic Cerebral Angiography’ by Osborn.^13^ These definitions build the foundation for the accurate association of the AVM vasculature to specific vascular parcellation units. Anatomical classification of the brain parenchyma adheres to the ‘Terminologia Anatomica’^14^ and as previously established by our group.^7,9,15^ At the lobar level, the anatomical classification includes the concept of a central lobe, entailing the precentral gyrus, postcentral gyrus, paracentral lobule and subcentral gyrus, excluding these structures from the frontal and parietal lobes, respectively. For segmentation of white matter sector infiltration, the classification proposed by Prof Gazi Yaşargil^2^ was utilized, which divides the white matter into five anatomical sectors based on a centrifugal principle.

#### Handling of missing data

For critical variables in the primary analysis, such as the presence of haemorrhage, seizures and functional neurological outcome in mRS at diagnosis and last visit, cases with missing data on these variables will be excluded from analyses (refer to inclusion and exclusion criteria). Thus, for primary and secondary outcomes, complete-case analysis will be performed for essential variables, which ensures that primary and secondary outcomes are based on complete and reliable data. Further, we believe that imputation could be misleading because overall missingness is rare (<5%), and most predictors (including anatomical annotations) are binary in nature—where imputation would artificially create potentially incorrect anatomical involvement with risk of misclassification. Instead, we will conduct a systematic assessment and visualization of missing data and evaluate the mechanism of missingness (MCAR, MAR or MNAR). For transparency, we will report rates of missing values per variable and plot missing data alongside non-missing data to allow for visual inspection of the missingness distribution.

### Ethical considerations

#### Ethics approval

This study is conducted in accordance with ethical standards and has received approval from the relevant ethics committee [Kantonale Ethikkommission Zürich (KEK), BASEC No. 2025-00510].

#### Data privacy and confidentiality

All project data will be recorded in electronic format. The project data will be stored on a secure server and all data files will be password-locked to restrict file access to authorized personnel only. Further, project data are only accessible to authorized personnel who require the data to fulfil their duties within the scope of the research project. In the projects database and any other project-specific documents, participants are only identified by a unique participant ID. The participants list, which links participant IDs to identities, will be stored separately and accessed only by authorized personnel.

### Use of large language models

All code and manuscript text were written by the authors. Subsequently, parts of code and text were refined with large language models for clarity and style. The authors verified all content and are responsible for the final work.

### Data analysis plan and statistical methods

#### Statistical software and guidelines

Statistical analyses will be performed in R or Python. The statistical code will be developed by the primary analyst (B.B.) and independently reviewed by a second investigator (K.A.). All analyses beyond pilot analyses will be performed after completion of data collection and strictly adhere to the statistical analysis plan. The study results will be reported following the STROBE (Strengthening the Reporting of Observational Studies in Epidemiology) guidelines, ensuring adherence to established reporting standards for observational research.^16^

#### Baseline characteristics

Baseline analyses will provide a comprehensive summary of patient demographics, AVM characteristics (such as side, eloquence, venous drainage pattern, Spetzler–Martin grade) and clinical outcomes to provide a concise understanding of the study cohort. Categorical variables will be presented as frequencies and percentages. Continuous variables will be summarized using means and standard deviations (SD).

#### Anatomical AVM phenotyping (topographic prevalence and topographic probability)

Topographic prevalence analysis will focus on determining the frequency with which AVMs affect specific anatomical structures, including parenchymal, arterial and venous units. Topographic prevalence represents the raw frequency of AVMs localized to particular anatomical structures without accounting for their relative volumes. This analysis will provide insights into the precise distribution patterns of AVMs across the brain's anatomy, forming the basis for subsequent investigations.

Topographic probability analysis normalizes AVM prevalence data by accounting for the variable sizes of anatomical structures. This approach calculates the probability by dividing the frequency of AVM involvement in a structure by the relative volume of this structure, as previously defined by our group.^15^ This methodology will be applied to gyral structures, which have consistent relative volumes across patients. In contrast, arterial and venous structures show high variability in diameter, length and morphology, influenced by individual anatomical differences and AVM-specific pathophysiological changes.

The results of these analyses will be presented using a combination of tabular and heatmap format. Tabular summaries will provide a concise overview of baseline characteristics and topographic prevalence data, which allows for convenient cross-referencing of raw frequencies and percentages between anatomical units. Artistic anatomical drawings will provide anatomical scaffolds for the depiction of frequencies in heatmap format. These visualizations will enhance the interpretability of complex spatial distribution patterns, highlighting regions with frequent AVM involvement.

#### Deconvolution of AVM meta-topologies

Phenotypic clustering will be performed to identify distinct meta-topologies of AVMs, grouping the structural complexity of fine-grained topographic prevalence data into a finite number of higher-order groupings. To do so, this study employs NNMF as an unsupervised approach to identify distinct meta-topologies. NNMF is particularly suited for this purpose due to its inherent non-negative framework, which allows for an intuitive interpretation of the underlying patterns. Unlike alternative dimensionality reduction methods such as principal component analysis, which may hinder interpretability by combining positive and negative contributions, NNMF offers easily accessible representations of the data.^17^ By integrating fine-grained parenchymal, arterial and venous features, we aim to explore spatial and functional interaction of these components, uncovering generalizable patterns of AVM topology.

NNMF models are fitted across *k* = 2–20 with 50 random starts per *k* and for each *k*, the standard NNMF validation measures are computed. Specifically, to determine the optimal number of meta-topologies (*k*), we will evaluate several quality metrics: the cophenetic correlation coefficient will be used to assess the stability of cluster assignments across repeated runs. The silhouette width will measure how well-separated and unique the resulting clusters are. The explained variance and residual sum of squares will be examined to evaluate how well the model approximates the original data. And finally, dispersion and consensus silhouette scores reflect overall clustering structure. The final value of *k* will be chosen by balancing these metrics together with clinical interpretability of the resulting meta-topologies.

Once meta-topologies are established, each meta-topology will be characterized by its defining parenchymal and vascular features and visualized using heatmaps and medical illustrations that highlight their most prominent traits. Additionally, the results will be summarized in tabular format, detailing the frequencies of key anatomical and morphological features for each meta-topology. Features with no variation across patients and patients with no positive anatomical features will be excluded.

#### Deciphering a spatiotemporal ontogenetic identity in AVMs

AVM meta-topologies are mapped to their corresponding ontogenetic units, uncovering potential developmental patterns of vulnerability and susceptibility. To enhance practicability and reproducibility of the approach, we have implemented a deterministic pipeline that begins with postnatal anatomical phenotyping of each AVM, revealing involvement of predefined parenchymal, arterial and venous units from MRI/DSA. Using the resulting matrix of anatomical prevalences, we apply NNMF to derive higher-order meta-topologies, as described above. Each adult topographic unit and meta-topology is systematically aligned with its ontogenetic counterpart in Phase 1 (neural tube), Phase 2 (vesicle formation) and Phase 3 (late maturation), such as mapping the frontal pole to the dorsal pallium, the middle cerebral artery (MCA) to the lateral striate artery and the cavernous sinus to the primary head vein. Specifically, we established fixed correspondence tables (refer to Supplementary Appendices 5–7), which specify the ontogenetic affiliation for every postnatal unit across Phase 1, Phase 2 and Phase 3, as predefined in the foundational work of Dr Pierre Lasjaunias, Dr Alejandro Berenstein, Dr George Linius Streeter and Dorcas Hager Padget.^18-21^ Finally, we compute ontogenetic topographic prevalences as the frequency of involvement per ontogenetic unit and phase, resulting in phase-specific developmental profiles and susceptibilities. The entire mapping pipeline is implemented in our publicly available R code, which enables user-friendly replication of all analyses. The frequency of AVMs within each ontogenetic unit will be visualized through heatmaps overlaid on artistic anatomical drawings of prenatal brain development as well as tabular formats. These visualizations will highlight temporal and spatial dynamics of AVM formation, offering insights into developmental vulnerabilities or predispositions that may underlie the observed anatomical patterns. Ultimately, ontogenetic mapping will help identify developmental vulnerabilities and phase-specific ontogenetic prevalences, revealing a developmental susceptibility landscape.

#### Linking AVM anatomy to clinical outcomes

Multivariate risk assessment will analyse associations between meta-topologies as well as other morphological features and the primary outcome (haemorrhage) as well as secondary outcomes (seizure occurrence and functional outcome). For categorical variables, Chi-square or Fisher’s exact tests will be applied, while continuous variables will be assessed using independent *t*-tests for normally distributed data or Mann–Whitney U-tests for non-normal distributions. Odds ratios (ORs) and their corresponding 95% CIs will be calculated to provide effect size estimates. Given the number of comparisons, *P*-values will be adjusted for multiple testing. Results will be displayed using forest plots. This analysis will elucidate the influence of meta-topologies and other risk factors on AVM-related complications.

In addition, we will conduct time-to-event analyses to assess the temporal relationship between AVM characteristics and primary and secondary outcomes, including time-to-haemorrhage, time-to-seizure and time-to-neurological decline. Given that AVMs are regarded as developmental malformations, with rare events of *de novo* formation in adults, time-to-event analyses will assume a starting point from birth and assess time until the occurrence of each event. As time-to-event analyses from diagnosis time may also be clinically meaningful (e.g. for communication of risks to patients after diagnosis and for planning of follow-up visits), we will additionally perform survival analyses anchored at diagnosis time, to allow for a direct comparison between lifetime natural-history risk and the risks observed after diagnosis. Cox’s regression models will be used to assess the impact of anatomical and morphological AVM features on the time-to-event, and hazard ratios (HRs) and their 95% CIs will be reported to quantify the strength of associations between predictors and outcomes. Censoring events include neurosurgical AVM resection, endovascular treatment, radiosurgery, a major non-AVM-related event (e.g. severe traumatic brain injury or stroke) and death. As diagnostic and treatment practices evolve over time, we will adjust for time of diagnosis in our primary analyses. Specifically, year of diagnosis will be included as a covariate to estimate era effects on haemorrhage risk. Further, we will present era-stratified and unstratified summaries to show whether our findings are consistent in earlier versus more recent periods. Results will be demonstrated using forest plots to display the estimated HRs and their CIs and Kaplan–Meier curves to illustrate the probability of event-free survival over time. Where relevant, we will translate relative effects into absolute risks over time. These analyses will provide insights into the temporal dynamics of AVM-related complications, contributing to a deeper understanding of their natural history over time.

#### Development of the anatomical staging system

To create a robust anatomical staging system for AVMs, the patient cohort will be randomly divided into two groups: a derivation cohort comprising 80% of the dataset and a validation cohort comprising the remaining 20%. This 80/20 split is chosen to maximize the power of model development while preserving an independent subset for external validation. The derivation cohort will serve as the foundation for all described analyses to this point, including topographic analyses, AVM phenotyping and constructing of the staging system. Sensitivity analyses will be conducted to test the robustness of findings, including the comparison of results across derivation and validation cohorts. AVMs will be stratified into distinct stages based on anatomical and morphological criteria identified through risk assessment. Once established, the staging system will undergo validation using the internal validation cohort, which was not included in initial analyses. This process will assess its predictive accuracy and reliability across key clinical outcomes. Further, we will subsequently perform an independent external validation of the finalized staging system in a separate tertiary care centre that is not part of the developmental phase. This external validation will aid generalizability to other tertiary care centres beyond the study institutions.

## Pilot data

The pilot data analyses are intended to validate data collection protocols, showcase the feasibility of the proposed approach and inform appropriate sample size calculations.

### Patient selection

**Figure 3:**
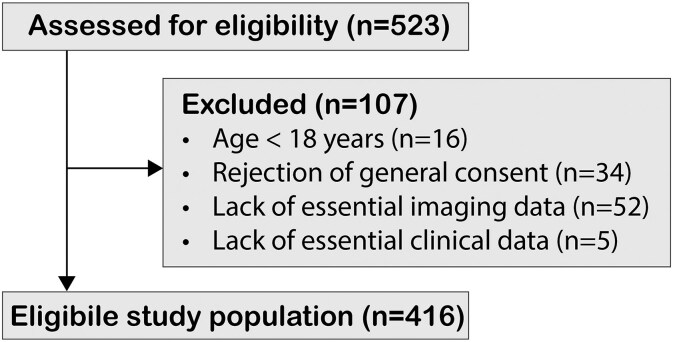
Flow diagram of patient eligibility and inclusion.

Of 523 patients assessed for eligibility at the University Hospital Zurich, 107 were excluded (16 due to age <18 years, 34 due to rejection of GC, 52 due to lack of essential imaging data and 5 due to lack of essential clinical data), leaving 416 patients in the eligible study population (Fig. 3).

### Baseline characteristics

An initial retrospective cohort (University Hospital Zurich) of 416 eligible patients with a confirmed diagnosis of brain AVMs was selected for pilot data collection and analysis. From the selected patients, baseline characteristics are summarized in Table 1. The mean age at diagnosis was 38.1 years (SD 18.59), with 49% of the cohort being female. Haemorrhage was the most common presenting feature, as shown in 36% of the patients, followed by seizures (24%), FND without haemorrhage (3.6%), and other symptoms such as headache or non-specific complaints (13%). Additionally, in 24% of the patients, the diagnosis was an incidental finding. In terms of lateralization, 46% of AVMs were found on the left side, 48% on the right and 6.0% were midline. Most AVMs (85%) were compact, while 15% were diffuse. Eloquent brain regions were involved in 34% of the cases, with the remaining 66% in non-eloquent areas. Venous drainage patterns showed that 39% of AVMs drained superficially, 31% had deep drainage and 30% demonstrated both drainage patterns. Spetzler–Martin grades were distributed across the cohort, highlighting a wide range of AVM complexities. The median follow-up time was 4.8 years (IQR 1.4–12.2). Further details are available in Supplementary Table 1.

**Table 1:** Baseline characteristics of the pilot cohort

Characteristic	*N* = 416^a^
Age	38.13 (18.59)
Female sex	205 (49%)
Clinical presentation	
Haemorrhage	149 (36%)
Seizure	98 (24%)
FND	15 (3.6%)
Other	54 (13%)
Incidental	100 (24%)
Side	
Midline	25 (6.0%)
Left	190 (46%)
Right	201 (48%)
Compactness	64 (15%)
Eloquence	143 (34%)
Venous drainage	
Both	123 (30%)
Deep	131 (31%)
Superficial	162 (39%)
Spetzler–Martin grade	
1	81 (19%)
2	169 (41%)
3	110 (26%)
4	47 (11%)
5	9 (2.2%)

^a^Mean (SD); *n* (%).

### Anatomical AVM phenotyping

#### Topographic prevalence

**Figure 4:**
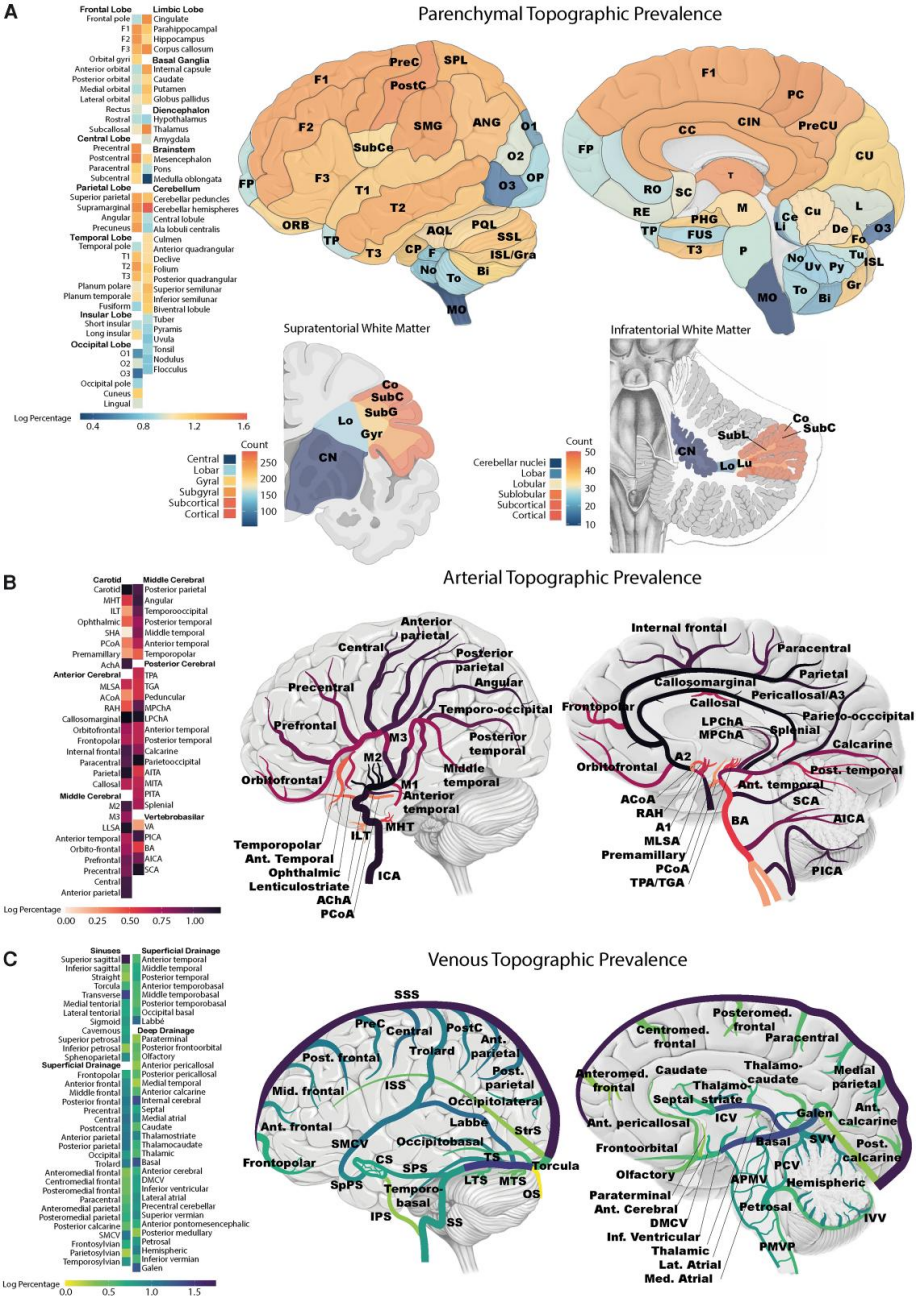
**Topographic prevalence of parenchymal, arterial and venous structures.** Graphical representation of AVM topographic prevalence across (**A**) parenchymal, (**B**) arterial and (**C**) venous structures. Each panel consists of a heatmap (left) depicting the proportion of AVMs affecting each anatomical unit and an anatomical illustration (right) with the heatmap overlaid for spatial reference. Abbreviations are listed in Supplementary Fig. 1.

**Figure 5:**
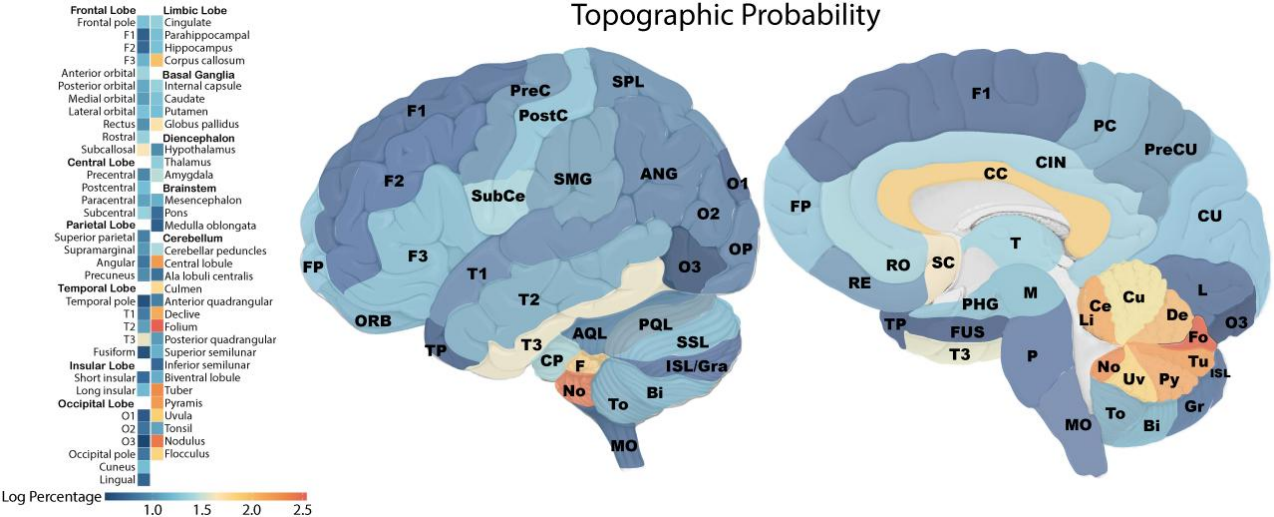
**Topographic probability of brain AVMs.** Graphical representation of AVM topographic probability across parenchymal structures. The heatmap (left) illustrates the probability of AVM occurrence in each anatomical unit after normalization for structure volume, while the anatomical illustration (right) overlays this probability distribution onto lateral and medial brain views. Abbreviations are listed in Supplementary Fig. 1.

The topographic prevalence of AVMs across parenchymal, arterial and venous structures reveals distinct patterns of anatomical susceptibilities. These findings are visually represented in Figs 4 and 5 with the raw frequency data listed in Supplementary Tables 2–4. Supplementary Fig. 1 serves as a structural reference.

Parenchymal topographic prevalence

AVMs demonstrated a distinct predilection for specific brain regions, with considerable variation across lobes and individual gyri. On a lobar level, the frontal lobe showed the highest prevalence (18%), followed by the parietal lobe and cerebellum (15% each). The central, temporal and limbic lobes were similarly affected (14% each). In contrast, the insula (4%) and brainstem (4%) were the least frequently involved.

At the sublobar level, the postcentral gyrus (7.5%) and precentral gyrus (6.5%) were the most commonly affected cortical regions, along with the supramarginal gyrus (6.5%), cingulate gyrus (6.3%) and corpus callosum (6.3%). Within the cerebellum, the superior semilunar lobule (4.1%) and folium (3.4%) were most frequently involved.

White matter involvement was assessed using the segmentation framework proposed by Yaşargil,^2^ which organizes white matter into cortico-ventricular anatomical sectors. In supratentorial AVMs, the cortical white matter sector was most frequently affected (68%), followed by subcortical (66%), subgyral (59%) and gyral white matter (50%). The central white matter was least involved (13%). In infratentorial AVMs, the cortical white matter (12%), subcortical (12%) and sublobular white matter (11%) were the dominant sectors, while central white matter involvement was rare (4%).

Arterial topographic prevalence

Brain AVMs were predominantly supplied by branches of the MCA, accounting for 61% of arterial feeders. The anterior cerebral artery (ACA) and posterior cerebral artery (PCA) each contributed 41%, while carotid artery feeders accounted for 16%. The external carotid artery contributed in 17% of cases and involvement of the vertebrobasilar system was less frequent, totalling 15%.

On a branch-specific level, the M4 cortical branches were the most common feeders, involved in 48% of cases. Other frequently involved MCA branches included the M1 segment (17%) and lateral lenticulostriate arteries (LLSA) (14%). From the ACA territory, the callosomarginal (17%) and pericallosal (17%) were the most frequently involved. The parieto-occipital artery (16%) was the dominant PCA contributor and the anterior choroidal artery (AchA) was the most common feeding branch from the internal carotid artery (11%). From the vertebrobasilar arteries, notable input was seen from the superior cerebellar artery (SCA) (14%) and posterior inferior cerebellar artery (PICA) (10%).

Venous topographic prevalence

Analysis of involved venous structures, the superior sagittal sinus was the most frequently involved (57%), followed by the transverse sinuses (24%). Regarding superficial cortical veins, the vein of Labbé (13%) was the most commonly involved, alongside the Rolandic (central) vein (12%) as well as the anterior and posterior parietal veins (11% each). Within the deep venous system, the internal cerebral vein was involved in 21% of the cases, while the basal vein and vein of Galen were affected in 19 and 17%, respectively.

#### Topographic probability

To account for the influence of anatomical volume on AVM distribution, prevalence data were normalized by structural size to assess the probability of AVM occurrence within each anatomical unit. This approach ensures that regions with a high prevalence of AVMs are not overrepresented due to their larger volume. The topographic probability analysis identified the folium as the structure with the highest AVM probability (83%), followed by the nodulus (61%), tuber (47%), pyramis (37%) and central lobule (38%). Other highly probable regions included the declive (28%) and corpus callosum (21%). These probability distributions are visualized in Fig. 5, where heatmaps illustrate AVM likelihood across parenchymal structures. Additionally, Supplementary Table 5 lists the topographic probability for each anatomical unit in detail.

### Ontogenetic mapping of AVM topographic prevalence

**Figure 6:**
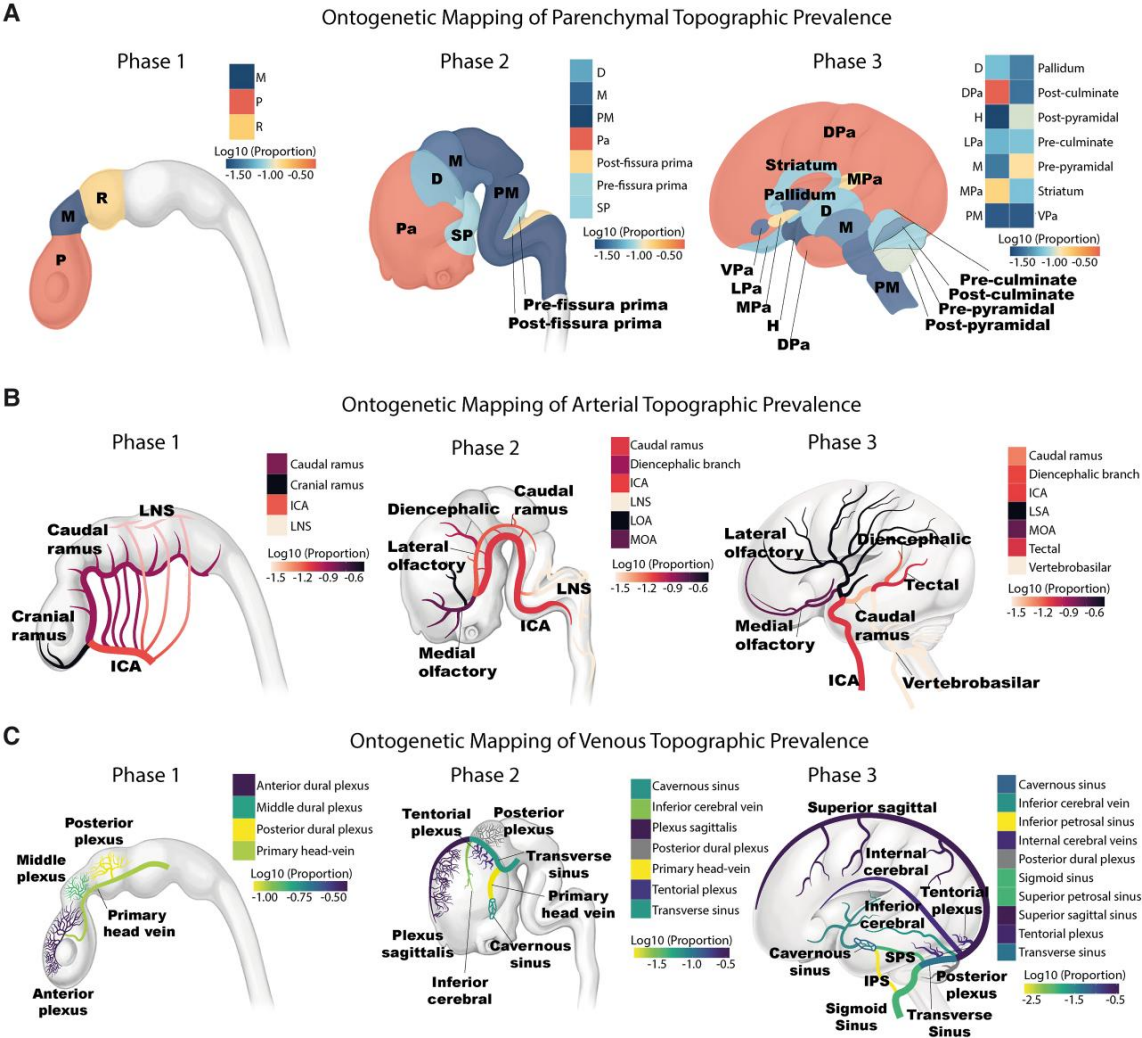
**Ontogenetic mapping of AVM topographic prevalence.** Heatmaps illustrating topographic prevalence data mapped to developmental phases for (**A**) parenchymal, (**B**) arterial and (**C**) venous structures. Each section depicts the evolving central nervous system across three key ontogenetic phases: Phase 1 (neural tube phase), Phase 2 (vesicle formation) and Phase 3 (late maturation phase). Abbreviations are listed in Supplementary Fig. 2.

To explore the developmental origins of AVMs, ontogenetic mapping was performed for parenchymal, arterial and venous structures, linking AVM anatomical phenotypes to anatomical units of distinct developmental phases: Phase 1 (neural tube phase), Phase 2 (vesicle formation) and Phase 3 (late maturation phase). Findings are visualized in Fig. 6, illustrating involvement of ontogenetic units, with a detailed breakdown listed in Supplementary Tables 6–8. Supplementary Fig. 2 serves as a structural reference.

#### Parenchymal ontogenetic mapping

AVM topographic prevalence data were mapped onto three major developmental phases. In Phase 1, the prosencephalon was the predominant site of AVM involvement (77.93%), followed by the mesencephalon (20.66%) and rhombencephalon (1.41%). In Phase 2, AVMs were most frequently associated with the pallium (69.25%), with additional involvement of the post-fissura prima (13.73%), pre-fissura prima (5.99%), subpallium (5.52%), diencephalon (3.17%), mesencephalon (1.41%) and the pontomedullary rhombencephalon (0.94%). By Phase 3, AVMs were predominantly localized to the dorsal pallium (54.23%), followed by the medial pallium (11.27%) and pre-pyramidal fissure (8.92%).

#### Arterial ontogenetic mapping

The developmental arterial supply of AVMs was mapped across three ontogenetic phases, revealing distinct temporal patterns of vascular vulnerability. In Phase 1, the arterial supply was primarily derived from the cranial ramus of the primitive carotid system (63.98%), with additional contributions from the caudal ramus (24.30%), internal carotid artery branches (8.75%) and the longitudinal neural system (2.97%). In Phase 2, AVMs were most frequently associated with the lateral olfactory artery system (42.37%) and the medial olfactory artery system (21.61%), alongside the diencephalic region (14.87%). By Phase 3, AVMs were predominantly supplied by the lateral striate system (42.37%), followed by the medial olfactory system (21.61%), tectal arteries (9.89%) and diencephalic arteries (8.40%), with smaller contributions from the caudal ramus, internal carotid and vertebrobasilar system.

#### Venous ontogenetic mapping

The venous drainage patterns of AVMs also demonstrated phase-specific developmental associations. In Phase 1, drainage was most frequently linked to the anterior dural plexus (51.46%), followed by the middle dural plexus (15.62%), posterior dural plexus (6.25%) and the primary head-vein (8.22%). In Phase 2, AVMs predominantly drained into the plexus sagittalis (35.58%) and tentorial plexus (20.97%), with additional contributions from the transverse sinus (6.25%), cavernous sinus (6.85%) and inferior cerebral vein (2.52%). By Phase 3, the most common drainage sites included the superior sagittal sinus (35.38%), internal cerebral veins (18.65%) and tentorial plexus (20.97%), with further drainage into the transverse sinus (5.09%), cavernous sinus (6.85%) and smaller contributions from other dural sinuses.

### Deconvolution of meta-topologies

**Figure 7:**
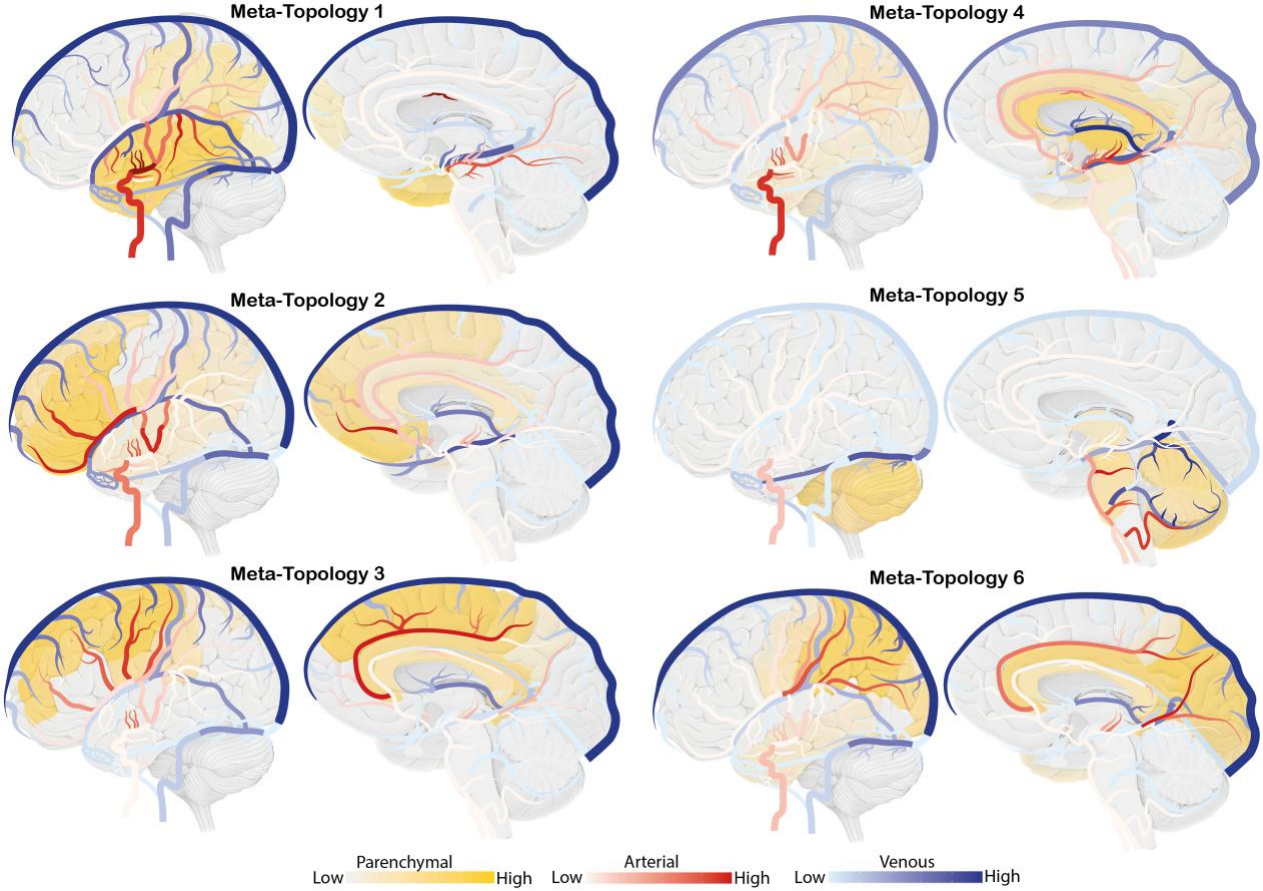
**Deconvolution of meta-topologies.** Graphical representation of six deconvoluted meta-topologies. Each panel consists of a heatmap projected onto a lateral (left) and medial (right) brain scaffold. Involvement across 86 parenchymal, 55 arterial and 57 venous units is colour-coded from frequent to infrequent: parenchymal units from yellow to light yellow, venous units from blue to light blue and arterial units from red to light red. Exact frequencies of involvement for all units are provided in Supplementary Tables 9 and 10. A structural reference map is displayed in Supplementary Fig. 1.

Unsupervised NNMF revealed six distinct meta-topologies based on anatomical feature patterns (Fig. 7). Each meta-topology showed a characteristic spatial signature as described below and detailed in Supplementary Tables 9 and 10.

- Meta-Topology 1 was defined by temporal lobe involvement with parenchymal T2 in 31% and T1 as well as T3 in 23%. Regarding arterial supply, temporal M4 branches were the most common contributors, with the anterior temporal artery in 25%, medial temporal artery in 39% and posterior temporal artery in 28%. Venous drainage was primarily into the superior sagittal (48%) and transverse sinus (38%), through the superficial middle cerebral vein (28%) and the vein of Labbe (27%).
- Meta-Topology 2 was characterized by strong frontal lobe involvement, particularly in orbitofrontal and medial frontal areas, including F3 in 33%, F2 in 24% and the orbital gyrus in 24%. Arterial supply was dominated by ACA-derived branches such as the orbitofrontal artery (37%), frontopolar (28%) and prefrontal M4 branches (31%). Venous drainage primarily involved the superior sagittal sinus (85%) and anterolateral cortical veins including the frontal veins (up to 28%).
- Meta-Topology 3 was defined by predominant affection of the central lobe with precentral and postcentral gyri involved in 32% and the paracentral lobule in 16%. Arterial supply included central (45%), anterior parietal (35%) and precentral branches (28%) of the MCA, with additional contributions from the callosomarginal artery (59%). Venous drainage was primarily via the superior sagittal sinus (98%), through central cortical veins (up to 38%) and less involvement of the internal cerebral veins (26%).
- Meta-Topology 4 demonstrated deep central brain involvement with predominant mapping to the thalamus (42%), the internal capsule (32%) and corpus callosum (28%). Arterial supply included the AChA (38%), LLSA (30%) and the lateral posterior choroidal artery (55%). Venous drainage was primarily via the internal cerebral veins (62%), medial atrial veins (17%) and the vein of Galen (18%).
- Meta-Topology 5 corresponded to an infratentorial cerebellar phenotype with extensive involvement of the vermis (35%) and the superior semilunar lobule (23%). Arterial supply was dominated by the SCA (78%) and PICA (58%). Venous drainage occurred through the petrosal vein (30%), the superior vermian vein (39%) and the vein of Galen (50%).
- Meta-Topology 6 involved parieto-occipital areas, with prominent involvement of the precuneus (25%), superior parietal lobule and supramarginal gyrus (22% each) and the cuneus in 16%. Arterial supply featured posterior M4 branches including the angular (43%) and posterior parietal (34%) arteries, with contributions from the parieto-occipital artery (63%) and calcarine artery (23%). Venous drainage included posterior parietal veins (38%), occipital veins (18%) and the vein of Galen (22%).

### Clinical risk assessment

**Figure 8:**
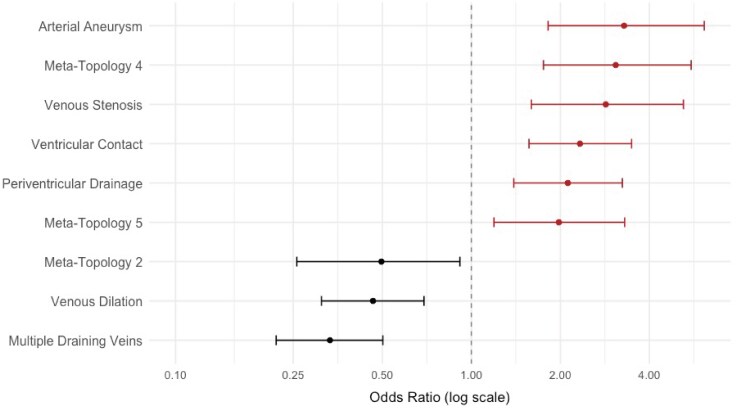
**Haemorrhage risk assessment.** The figure displays adjusted ORs with 95% CIs from a univariate logistic regression model assessing the association between individual anatomical features and haemorrhage risk. The analysis was performed on 416 patients.

Univariate logistic regression analysis identified several significant anatomical predictors of haemorrhage (Fig. 8). The presence of an arterial aneurysm was the strongest risk factor (OR = 3.28; *P* < 0.001), followed by venous stenosis (OR = 2.85; *P* < 0.001), ventricular contact (OR = 2.33; *P* < 0.001) and periventricular venous drainage (OR = 2.12; *P* < 0.001). In contrast, the presence of multiple draining veins (OR = 0.33; *P* < 0.001) and venous dilatation (OR = 0.47; *P* = 0.002) were significantly associated with a reduced risk of haemorrhage. Among the meta-topologies, Meta-Topology 4 (OR = 3.08; *P* < 0.001) and Meta-Topology 5 (OR = 1.98; *P* = 0.008) were positively associated with haemorrhage, whereas Meta-Topology 2 showed a protective association (OR = 0.50; *P* = 0.029).

## Supplementary Material

fcag039_Supplementary_Data

## Data availability

All anonymized study data and analysis code of the pilot analysis were made publicly accessible at zenodo (doi: 10.5281/zenodo.16755262) to ensure reproducibility of the results. Similarly, all code and datasets for the main study will be made publicly accessible upon acceptance for publication of the final manuscript.
