# Supplementary material for: Anatomical phenotyping and staging of brain arteriovenous malformations
Source: Brain Commun. 2026 Feb 8;8(1):fcag039. doi: 10.1093/braincomms/fcag039 (PMC12917541; doi:10.1093/braincomms/fcag039)
Supplement: fcag039_Supplementary_Data [file fcag039_supplementary_data.zip › Appendices.pdf]

APPENDICES

1. Database Legend
2. Definition of Parenchymal Units
3. Definition of Arterial Units
4. Definition of Venous Units
5. Ontogenetic Parcellation of Parenchymal Units
6. Ontogenetic Parcellation of Arterial Units
7. Ontogenetic Parcellation of Venous Units

Appendix 1: AVM Database Definitions
Patient Information

- **birth_year**: Birth year (yyyy)
- **diagnosis_date**: Date of AVM diagnosis (dd.mm.yyyy)
- **censoring_date**: Date of censoring event (dd.mm.yyyy)
- **censoring_event:** Censoring event (operation, embolization, radiosurgery, or last followup)
- **sex**: Sex of the patient (Female: F; Male: M; Other: O)

Clinical Presentation

- **presenting_feature**: Initial clinical presentation:
  - FND: Focal Neurological Deficit (prior to or without hemorrhage)
  - hemorrhage: Hemorrhage
  - seizure: Seizure
  - other: Other symptoms (e.g. headache, tinnitus, dizziness)
  - incidental: Incidental diagnosis
- **hemorrhage**: Presence of hemorrhage at any time at diagnosis or during follow-up until the censoring event (No: 0; Yes: 1)
- **hemorrhage_date**: Date of first hemorrhage (dd.mm.yyyy)
- **seizure**: Seizure history (No: 0; Yes before bleeding: 1; Yes after bleeding: 2)
- **seizure_date**: Date of first seizure (dd.mm.yyyy)
- **semiology**: Seizure semiology based on the ILAE 2017 Classification of Seizure Types. If multiple semiologies are known, the most prevalent is chosen.
  - 0: No seizure
  - 1: Focal aware motor
  - 2: Focal aware nonmotor
  - 3: Focal impaired motor
  - 4: Focal impaired nonmotor
  - 5: Generalized motor
  - 6: Generalized nonmotor
  - 7a: Unknown motor
  - 7b: Unknown nonmotor
  - 8: Focal to bilateral tonic-clonic
- **secondary_generalization**: Occurrence of a generalized seizure at any time point (No: 0; Yes: 1)
- **FND**: Focal neurological deficits (No: 0; Yes before bleeding: 1; Yes after bleeding: 2)
- **FND_date**: Date of first focal neurological deficit (dd.mm.yyyy)

Outcome and Follow-up

- **GCS_admission**: Glasgow Coma Scale (GCS) score at first diagnosis
- **mRS_diagnosis**: Modified Rankin Scale (mRS) score at first diagnosis
- **mRS_last**: Modified Rankin Scale score at last follow-up
- **last_followup_date**: Date of last follow-up visit (dd.mm.yyyy)
- **mortality**: AVM-related mortality (No: 0; Yes: 1)
- **death_date**: Date of death, if related to AVM (dd.mm.yyyy)

Treatment

- **treatment**: Surgical, endovascular, or radiosurgical treatment received (No: 0; Yes: 1)
- **surgery**: Surgical AVM resection performed (No: 0; Yes: 1)
- **embolization**: Endovascular AVM treatment performed (No: 0; Yes: 1)
- **radiosurgery**: Radiosurgical AVM treatment performed (No: 0; Yes: 1)

Hemorrhage

- **IVH**: Presence of intraventricular hemorrhage (No: 0; Yes: 1)
- **ICB**: Presence of intraparenchymal hemorrhage (No: 0; Yes: 1)
- **SAH**: Presence of subarachnoid hemorrhage (No: 0; Yes: 1)
- **SDH**: Presence of subdural hematoma (No: 0; Yes: 1)

Parenchymal Involvement and AVM Phenotype

- **diameter_ml**: Diameter of the AVM nidus on axial MRI sequences (medial to lateral) in mm
- **diameter_ap**: Diameter of the AVM nidus on sagittal MRI sequences (anterior to posterior) in mm
- **diameter_cc**: Diameter of the AVM nidus on coronal MRI sequences (cranial to caudal) in mm
- **avm_volume**: AVM nidus volume calculated as: diameter_ml x diameter_cc x diameter_ap / 2, in cubic centimeters (cc)
- **plexiform**: Type of AVM nidus (Plexiform: 1; Fistulous: 0)
- **compactness**: Compact: 1; Diffuse: 0. Compactness is a qualitative assessment of the AVM nidus based on angiographic appearance. Compact AVMs are recognized as a tight tangle of vessels on angiography, with little brain tissue within the nidus and well-defined margins between brain and AVM.
- **side**: Lateralization of the AVM (Left: 1; Right: 2)

Vascular characteristics

- **superficial_venous_drainage** (No: 0; Yes: 1)
- **deep_venous_drainage** (No: 0; Yes: 1)
    - Superficial drainage is considered present, if all the drainage from the AVM is through the cortical venous system. The venous pattern is considered deep if any or all of the drainage is through deep veins (such as the internal cerebral veins, basal veins, or precentral cerebellar vein). In the posterior fossa, only cerebellar hemispheric veins that drain directly into the straight sinus, torcula, or transverse sinus are considered to be superficial.
- **nidal_aneurysm**: Presence of a nidal aneurysm (No: 0; Yes: 1). Nidal is defined as contiguous with the vascular mass included in the AVM size measurement. The aneurysm may extend past the margin of the actual measured AVM mass.
- **a_aneurysm**: Presence of an arterial aneurysm (No: 0; Yes: 1). Arterial aneurysms are defined as saccular luminal dilatations of any feeding artery proximal to the nidus.
- **v_aneurysm**: Presence of saccular venous aneurysm (No: 0; Yes: 1). Venous aneurysms are defined as saccular luminal dilatations of any draining vein distal to the nidus.
- **a_dilation**: Presence of arterial dilatation. A feeding artery is considered dilated, if its diameter is at minimum two times the size of the same arterial segment of the contralateral side (No: 0; Yes: 1)
- **a_diameter**: The largest arterial diameter directly proximal to the AVM nidus
- **a_number**: Number of feeding arteries that directly feed the nidus
- **v_dilatation**: Presence of venous dilatation (No: 0; Yes: 1). A draining vein is considered dilated, if its diameter is at minimum two times the size of the same venous segment of the contralateral side. Venous aneurysms and focal venous ectasia are not considered in this category.
- **venous_diameter**: If there is nonuniformity of venous caliber, the largest draining vein's diameter at the exit from the nidus is used. Venous aneurysms and focal ectasia are not measured.
- **v_ectasia:** Presence of venous ectasia. Venous ectasia is defined as a >2-fold caliber change in any draining venous channel which is not due to stenosis (No: 0; Yes: 1).
- **ectasia_diameter**: Maximum diameter of the venous ectasia in mm
- **stenosis**: Presence of venous stenosis (No: 0; Yes: 1). Venous stenosis is defined as > 50% stenosis of any primary draining vein.
- **periventricular_drainage:** Presence of periventricular venous drainage, defined as a draining vein in direct contact to the ventricular system (No: 0; Yes: 1)
- **v_number**: Number of draining veins, that originate directly from the AVM nidus.

Appendix 2: Definition of Parenchymal Units

Anatomical Structure (Abbreviation)	Anatomical Boundaries
**Frontal lobe**<br>**(frontal_lobe)**	Rostral: Anterior parolfactory sulcus;<br>Caudal: Precentral sulcus (lateral surface), paracentral sulcus (medial surface);<br>Ventral: -<br>Dorsal: -<br>Medial: Cingulate sulcus;<br>Lateral: Anterior periinsular sulcus (anterior), superior periinsular sulcus (posterior).
**Central lobe**<br>**(central_lobe)**	Rostral: Precentral sulcus (lateral surface), paracentral sulcus (medial surface);<br>Caudal: Postcentral sulcus (lateral surface), marginal sulcus (medial surface);<br>Ventral: -<br>Dorsal: -<br>Medial: Cingulate sulcus;<br>Lateral: Superior periinsular sulcus.
**Parietal lobe**<br>**(parietal_lobe)**	Rostral: Postcentral sulcus (lateral surface), marginal sulcus (medial surface);<br>Caudal: Parieto-occipital sulcus (medial surface), parieto-occipital line (lateral surface: line following the anterior occipital sulcus connecting the superior end of the parieto-occipital sulcus and the preoccipital notch);<br>Ventral: -<br>Dorsal: -<br>Medial: Subparietal sulcus<br>Lateral: Parieto-temporal line (arbitrary line between the posterior ramus of the Sylvian fissure and the preoccipital notch).
**Occipital lobe**<br>**(occipital_lobe)**	Rostral: Parieto-occipital sulcus (medial surface), parieto-occipital line (lateral surface: line following the anterior occipital sulcus connecting the superior end of the parieto-occipital sulcus and the preoccipital notch); Occipitotemporal line (inferior surface: arbitrary

	line connecting the inferior end of the parieto-occipital sulcus and the preoccipital notch);<br>Caudal: -<br>Ventral: -<br>Dorsal: -<br>Medial: -<br>Lateral: -
**Temporal lobe**<br>**(temporal_lobe)**	Rostral: -<br>Caudal: Parieto-temporal line (lateral surface: arbitrary line between the posterior ramus of the Sylvian fissure and the preoccipital notch), occipitotemporal line (inferior surface: arbitrary line connecting the inferior end of the parieto-occipital sulcus and the preoccipital notch);<br>Ventral: -<br>Dorsal: Inferior periinsular sulcus;<br>Medial: Collateral sulcus (posterior), rhinal sulcus (anterior);<br>Lateral: -
**Insular lobe**<br>**(insular_lobe)**	Rostral: Anterior periinsular sulcus;<br>Caudal: Posterior insular point;<br>Ventral: Posterior periinsular sulcus;<br>Dorsal: Superior periinsular sulcus;<br>Medial: Limen insulae;<br>Lateral: -
**Limbic lobe**<br>**(limbic_lobe)**	Rostral: Anterior parolfactory sulcus (anterior), lamina terminalis (posterior);<br>Caudal: Subparietal sulcus;<br>Ventral: Collateral sulcus (posterior), rhinal sulcus (anterior);<br>Dorsal: Cingulate sulcus;<br>Medial: Pericallosal sulcus;<br>Lateral: Limen insulae.

CEREBRAL GYRAL SEGMENTS	
**Frontal pole**<br>**(frontal_pole)**	Anterior most aspect of the medial, lateral and inferior surface of the frontal lobe containing the superior, middle and inferior frontopolar gyri;<br>Caudal: Superior frontal gyrus, medial frontal gyrus, rostral gyrus, gyrus rectus and anterior orbital gyrus.
**Superior frontal gyrus**<br>**(F1)**	Rostral: Frontal pole, rostral gyrus;<br>Caudal: Precentral sulcus (lateral surface), paracentral sulcus (medial surface);<br>Ventral: -<br>Dorsal: -<br>Medial: Cingulate sulcus;<br>Lateral: Superior frontal sulcus.
**Middle frontal gyrus**<br>**(F2)**	Rostral: Frontal pole;<br>Caudal: Precentral sulcus;<br>Ventral: -<br>Dorsal: -<br>Medial: Superior frontal sulcus;<br>Lateral: Inferior frontal sulcus.
**Inferior frontal gyrus, orbital part**<br>**(F3orbital)**	Rostral: Frontoorbital sulcus;<br>Caudal: Horizonal ramus of the Sylvian fissure;<br>Ventral: Anterior periinsular sulcus;<br>Dorsal: Inferior frontal sulcus;<br>Medial: -<br>Lateral: -
**Inferior frontal gyrus, triangular part**<br>**(F3triangular)**	Rostral: Horizonal ramus of the Sylvian fissure;<br>Caudal: Ascending ramus of the Sylvian fissure;<br>Ventral: Anterior insular point;<br>Dorsal: Inferior frontal sulcus;<br>Medial: -<br>Lateral: -

**Inferior frontal gyrus, opercular part**<br>**(F3opercular)**	Rostral: Ascending ramus of the Sylvian fissure;<br>Caudal: Precentral sulcus;<br>Ventral: Superior periinsular sulcus;<br>Dorsal: Inferior frontal sulcus;<br>Medial: -<br>Lateral: -
**Anterior orbital gyrus**<br>**(ORBant)**	Rostral: Frontal pole;<br>Caudal: Transverse orbital sulcus;<br>Ventral: -<br>Dorsal: -<br>Medial: Medial orbital sulcus (rostral part)<br>Lateral: Lateral orbital sulcus (caudal part)
**Medial orbital gyrus**<br>**(ORBmed)**	Rostral: Frontal pole;<br>Caudal: Transverse insular gyrus, anterior periinsular sulcus;<br>Ventral: -<br>Dorsal: -<br>Medial: Olfactory sulcus;<br>Lateral: Medial orbital sulcus.
**Lateral orbital gyrus**<br>**(ORBlat)**	Rostral: Frontal pole;<br>Caudal: Anterior periinsular sulcus;<br>Ventral: -<br>Dorsal: -<br>Medial: Lateral orbital sulcus;<br>Lateral: Frontoorbital sulcus.
**Posterior orbital gyrus**<br>**(ORBpost)**	Rostral: Transverse orbital sulcus;<br>Caudal: Transverse insular gyrus, anterior periinsular sulcus;<br>Ventral: -<br>Dorsal: -<br>Medial: Olfactory sulcus;

	Lateral: Frontoorbital sulcus.
**Gyrus rectus (rectus)**	Rostral: Frontal pole;<br>Caudal: Anterior parolfactory sulcus;<br>Ventral: -<br>Dorsal: Inferior rostral sulcus;<br>Medial: -<br>Lateral: Medial orbital sulcus.
**Rostral gyrus (rostral)**	Rostral: Frontal pole;<br>Caudal: Anterior parolfactory sulcus;<br>Ventral: Inferior rostral sulcus;<br>Dorsal: Cingulate sulcus;<br>Medial: -<br>Lateral: -
**Subcallosal area (subcallosal)**	*Comprising the parolfactory gyrus/gyri and the paraterminal gyrus;*<br>Rostral: Anterior parolfactory sulcus;<br>Caudal: Lamina terminalis;<br>Ventral: Diagonal band of Broca;<br>Dorsal: Rostrum of corpus callosum (pericallosal sulcus);<br>Medial: -<br>Lateral: -
**Precentral gyrus (precentral)**	Rostral: Precentral sulcus;<br>Caudal: Central (Rolandic) sulcus;<br>Ventral: -<br>Dorsal: -<br>Medial: Interhemispheric fissure;<br>Lateral: Subcentral gyrus.
**Postcentral gyrus (postcentral)**	Rostral: Central (Rolandic) sulcus;<br>Caudal: Postcentral sulcus;<br>Ventral: -

	Dorsal: -<br>Medial: Interhemispheric fissure;<br>Lateral: Subcentral gyrus.
**Paracentral lobule (paracentral)**	Rostral: Paracentral sulcus;<br>Caudal: Marginal sulcus;<br>Ventral: Cingulate sulcus;<br>Dorsal: Interhemispheric fissure;<br>Medial: -<br>Lateral: -
**Subcentral gyrus (subcentral)**	Rostral: Anterior subcentral sulcus;<br>Caudal: Posterior subcentral sulcus;<br>Ventral: -<br>Dorsal: Precentral gyrus (anterior), postcentral sulcus (posterior);<br>Medial: Superior periinsular sulcus;<br>Lateral: -
**Superior parietal lobule (SPL)**	Rostral: Postcentral sulcus;<br>Caudal: Parieto-occipital line (line following the anterior occipital sulcus connecting the superior end of the parieto-occipital sulcus and the preoccipital notch);<br>Ventral: -<br>Dorsal: -<br>Medial: Interhemispheric fissure;<br>Lateral: Intraparietal sulcus.
**Supramarginal gyrus (SMG)**	Rostral: Postcentral sulcus (superior), posterior subcentral sulcus (inferior);<br>Caudal: Intermediate sulcus of Jensen;<br>Ventral: Superior periinsular sulcus, posterior insular point, inferior periinsular sulcus;<br>Dorsal: -<br>Medial: Intraparietal sulcus;

	Lateral: Parieto-temporal line (arbitrary line between the posterior ramus of the Sylvian fissure and the preoccipital notch).
**Angular gyrus (ANG)**	Rostral: Intermediate sulcus of Jensen;<br>Caudal: Parieto-occipital line (line following the anterior occipital sulcus connecting the superior end of the parieto-occipital sulcus and the preoccipital notch);<br>Ventral: -<br>Dorsal: -<br>Medial: Intraparietal sulcus;<br>Lateral: Parieto-temporal line (arbitrary line between the posterior ramus of the Sylvian fissure and the preoccipital notch).
**Precuneus (precuneus)**	Rostral: Marginal sulcus;<br>Caudal: Parieto-occipital sulcus;<br>Ventral: Subparietal sulcus;<br>Dorsal: Interhemispheric fissure;<br>Medial: -<br>Lateral: -
**Cuneus (cuneus)**	Rostral: Parieto-occipital sulcus;<br>Caudal: Interhemispheric fissure;<br>Ventral: Calcarine sulcus;<br>Dorsal: Interhemispheric fissure;<br>Medial: -<br>Lateral: Interhemispheric fissure.
**Superior occipital gyrus (O1)**	Rostral: Parieto-occipital line (line following the anterior occipital sulcus connecting the superior end of the parieto-occipital sulcus and the preoccipital notch);<br>Caudal: Occipital pole;<br>Ventral: -<br>Dorsal: -<br>Medial: Interhemispheric fissure;

	Lateral: Intraoccipital sulcus.
**Middle occipital gyrus (O2)**	Rostral: Parieto-occipital line (line following the anterior occipital sulcus connecting the superior end of the parieto-occipital sulcus and the preoccipital notch);<br>Caudal: Occipital pole;<br>Ventral: -<br>Dorsal: -<br>Medial: Intraoccipital sulcus;<br>Lateral: Inferior occipital sulcus.
**Inferior occipital gyrus (O3)**	Rostral: Parieto-occipital line (line following the anterior occipital sulcus connecting the superior end of the parieto-occipital sulcus and the preoccipital notch);<br>Caudal: Occipital pole;<br>Ventral: Retrolingual sulcus;<br>Dorsal: -<br>Medial: Inferior occipital sulcus;<br>Lateral: -
**Occipital pole (occipital_pole)**	Posterior most aspect of the medial, lateral and inferior surface of the occipital lobe;<br>Rostral: Superior occipital gyrus, medial occipital gyrus, posterior occipital gyrus, cuneus and lingual gyrus.
**Lingual gyrus (lingual)**	Rostral: Truncus fissurae parietooccipitalis et calcarinae;<br>Caudal: Occipital pole;<br>Ventral: -<br>Dorsal: Calcarine sulcus;<br>Medial: -<br>Lateral: Collateral sulcus.
**Fusiform gyrus (fusiform)**	Rostral: Temporal pole;<br>Caudal: Junction between the collateral sulcus (medially) and the (lateral) occipitotemporal sulcus (laterally);

	Ventral: -<br>Dorsal: -<br>Medial: Collaterals sulcus;<br>Lateral: (Lateral) occipitotemporal sulcus.
**Superior temporal gyrus**<br>**(T1total)**	Rostral: Temporal pole;<br>Caudal: Parieto-temporal line (arbitrary line between the posterior ramus of the Sylvian fissure and the preoccipital notch);<br>Ventral: Superior temporal sulcus;<br>Dorsal: Sylvian fissure/Transverse temporal gyri/sulci (of Heschl and Schwalbe);<br>Medial: -<br>Lateral: -
**Middle temporal gyrus**<br>**(T2total)**	Rostral: Temporal pole;<br>Caudal: Parieto-temporal line (arbitrary line between the posterior ramus of the Sylvian fissure and the preoccipital notch);<br>Ventral: Inferior temporal sulcus;<br>Dorsal: Superior temporal sulcus;<br>Medial: -<br>Lateral: -
**Inferior temporal gyrus**<br>**(T3total)**	Rostral: Temporal pole;<br>Caudal: Parieto-temporal line (arbitrary line between the posterior ramus of the Sylvian fissure and the preoccipital notch);<br>Ventral: -<br>Dorsal: Inferior temporal sulcus<br>Medial: (Lateral) occipitotemporal sulcus;<br>Lateral: -
**Planum temporale**<br>**(planum_emporale)**	*Comprising the posterior transverse temporal gyri of Heschl;*<br>Rostral: Transverse temporal gyri/sulci of Schwalbe;

	Caudal: Terminal (ascending and descending) limbs of the Sylvian fissure;<br>Ventral: -<br>Dorsal: Posterior insular point;<br>Medial: Inferior periinsular sulcus;<br>Lateral: Sylvian fissure/superior temporal gyrus.
**Planum polare**<br>**(planum_polare)**	*Comprising the anterior transverse temporal gyri of Schwalbe;*<br>Rostral: Temporal pole;<br>Caudal: Transverse temporal gyri/sulci of Heschl;<br>Ventral: -<br>Dorsal: Inferior periinsular sulcus;<br>Medial: Limen insulae;<br>Lateral: Sylvian fissure/superior temporal gyrus.
**Temporal pole**<br>**(temporal_pole)**	Anterior most aspect of the medial, lateral, superior and inferior surface of the temporal lobe; Caudal: Superior temporal gyrus, middle temporal gyrus, inferior temporal gyrus, fusiform gyrus, parahippocampal gyurs and planum polare.
**Short insular gyri**<br>**(INSshort)**	Rostral: Anterior periinsular sulcus;<br>Caudal: Central insular sulcus;<br>Ventral: -<br>Dorsal: Superior periinsular sulcus;<br>Medial: Limen insulae;<br>Lateral: Anterior insular point.
**Long insular gyri**<br>**(INSlong)**	Rostral: Central insular sulcus;<br>Caudal: Posterior insular point;<br>Ventral: -<br>Dorsal: Superior periinsular sulcus;<br>Medial: Limen insulae;<br>Lateral: Inferior periinsular sulcus.

**Parahippocampal gyrus**<br>**(PHG)**	Rostral: Rhinal sulcus, temporal incisure;<br>Caudal: Truncus fissurae parietooccipitalis et calcarinae;<br>Ventral: -<br>Dorsal: Parasubiculum;<br>Medial: -<br>Lateral: Collateral sulcus, rhinal sulcus.
**Cingulate gyrus**<br>**(cingulate)**	*Divided into an anterior (= ascending), middle (=horizontal) and posterior (= descending) part.*<br>Rostral: Anterior parolfactory sulcus;<br>Caudal: Truncus fissurae parietooccipitalis et calcarinae;<br>Ventral: Pericallosal sulcus;<br>Dorsal: Cingulate sulcus, subparietal sulcus;<br>Medial: -<br>Lateral: -
**CENTRAL PROSENCEPHALON**	
**Corpus callosum**<br>**(corpus_callosum)**	*Composed of the rostrum, genu, trunk/body and splenium.*<br>Rostral: Lamina terminalis;<br>Caudal: -<br>Ventral: Lateral ventricle;<br>Dorsal: Pericallosal sulcus;<br>Medial: -<br>Lateral: -
**Putamen**<br>**(putamen)**	Rostral: Internal capsule;<br>Caudal: Internal capsule;<br>Ventral: Internal capsule;<br>Dorsal: Internal capsule;<br>Medial: Globus pallidus;<br>Lateral: External capsule.

**Caudate nucleus (caudate)**	*Composed of a head, body and tail.*<br>Bordered by the lateral ventricle and the internal capsule.
**Globus pallidus (globus_pallidus)**	*Composed of an internal and external part.*<br>Rostral: Internal capsule;<br>Caudal: Internal caplsue;<br>Ventral: Internal capsule;<br>Dorsal: Internal capsule;<br>Medial: Internal capsule;<br>Lateral: Putamen.
**Internal capsule (internal_capsule)**	Rostral: Caudate nucleus<br>Caudal: Thalamus;<br>Ventral: Mesencephalon;<br>Dorsal: Caudate nucleus;<br>Medial: Caudate nucleus (anterior), thalamus (posterior),<br>Lateral: Globus pallidus, putamen.
**Hypothalamus (hypothalamus)**	Rostral: Lamina terminalis;<br>Caudal: Mesencephalon;<br>Ventral: Floor of third ventricle;<br>Dorsal: Hypothalamic sulcus;<br>Medial: Third ventricle;<br>Lateral: Innominate substance, anterior commissure, inferior thalamic peduncle.
**Thalamus (thalamus)**	Rostral: Foramen of Monro;<br>Caudal: Velum interpositum cistern;<br>Ventral: Mesencephalon;<br>Dorsal: Lateral ventricle;<br>Medial: Third ventricle;<br>Lateral: Internal capsule.

**Hippocampus**<br>**(hippocampus)**	*Comprising the parasubiculum, presubiculum, subiculum, dentate gyrus, CA1-CA3 field of Ammon's horn.*<br>Rostral: Amygdala;<br>Caudal: Fornix;<br>Ventral: Hippocampal sulcus;<br>Dorsal: temporal horn of lateral ventricle, choroid fissure;<br>Medial: Crural cistern:<br>Lateral: Temporal horn of lateral ventricle.
**Amygdala**<br>**(amygdala)**	Rostral: Limen insulae;<br>Caudal: Temporal horn of lateral ventricle, hippocampus;<br>Ventral: Temporal horn of lateral ventricle, hippocampus;<br>Dorsal: Crural cistern, innominate substance;<br>Medial: Crural cistern, innominate substance;<br>Lateral: Limen insulae, temporal horn of lateral ventricle.
**BRAINSTEM**	
**Mesencephalon**<br>**(mesencephalon)**	Rostral: Mamillary bodies; optic tract, lateral geniculate body, pulvinar thalami, posterior commissure;<br>Caudal: Pontomesencephalic sulcus.
**Pons**<br>**(pons)**	Rostral: Pontomesencephalic sulcus;<br>Caudal: Pontomedullary sulcus.
**Medulla oblongata**<br>**(medulla)**	Rostral: Pontomedullary sulcus;<br>Caudal: Lower border of pyramidal decussation.
**CEREBELLUM**	
**Cerebellar lobes**	
**Anterior**<br>**(anterior_cerebellar_lobe)**	Comprising the vinculum and lingula (not segmented in this study due to the small size and indistinct identifiability on the MRI), central lobule and ala lobuli centralis, culmen and anterior quadrangular lobule *(definitions below)*.

	Separated from the medial cerebellar lobe by the primary cerebellar fissure.
**Medial (medial_cerebellar_lobe)**	Comprising the declive and posterior quadrangular lobule, folium and superior semilunar lobule *(definitions below)*. Separated from the posterior cerebellar lobe by the horizontal cerebellar fissure .
**Posterior (posterior__cerebellar_lobe)**	Comprising the tuber and inferior semilunar/gracile lobule; pyramis and biventer lobule, uvula and tonsil *(definitions below)*. Separated from the flocculonodular cerebellar lobe by the posterolateral cerebellar fissure.
**Flocculonodular (flocculonodular)**	Comprising the nodulus and flocculus *(definitions below)*.
**Vermian lobules (vermis)**	Bilateral: Paravermal fissure.
**Central (central_lobule)**	Rostral: Precentral cerebellar fissure;<br>Caudal: Preculminate cerebellar fissure.
**Culmen (culmen)**	Rostral: Preculminate cerebellar fissure;<br>Caudal: Primary cerebellar fissure.
**Declive (declive)**	Rostral: Primary cerebellar fissure;<br>Caudal: Postclival (posterior superior) cerebellar fissure.
**Folium (folium)**	Rostral: Postclival (posterior superior) cerebellar fissure;<br>Caudal: Horizontal cerebellar fissure.
**Tuber (tuber)**	Rostral: Horizontal cerebellar fissure;<br>Caudal: Prepyramidal (prebiventer) cerebellar fissure.
**Pyramid (pyramid)**	Rostral: Prepyramidal (prebiventer) cerebellar fissure;<br>Caudal: Secondary (postpyramidal) cerebellar fissure.
**Uvula (uvula)**	Rostral: Secondary (postpyramidal) cerebellar fissure;<br>Caudal: Posterolateral fissure.
**Nodule**	Rostral: Posterolateral fissure;

**(nodule)**	Caudal: Inferior medullary velum.
**Cerebellar hemispheric lobules (cerebellar_hemispheres)**	Bimedial: Paravermal fissure
**Ala lobuli centralis (ala_lobuli_centralis)**	Rostral: Precentral cerebellar fissure;<br>Caudal: Preculminate cerebellar fissure.
**Anterior quadrangular lobule (anterior_quadrangular)**	Rostral: Preculminate cerebellar fissure;<br>Caudal: Primary cerebellar fissure.
**Posterior quadrangular lobule (posterior_quadrangular)**	Rostral: Primary cerebellar fissure;<br>Caudal: Postclival (posterior superior) cerebellar fissure.
**Superior semilunar lobule (superior_semilunar)**	Rostral: Postclival (posterior superior) cerebellar fissure;<br>Caudal: Horizontal cerebellar fissure.
**Inferior semilunar / gracile lobule (inferior_semilunar)**	Rostral: Horizontal cerebellar fissure;<br>Caudal: Prepyramidal (prebiventer) cerebellar fissure.
**Biventer lobule (biventral_lobule)**	Rostral: Prepyramidal (prebiventer) cerebellar fissure;<br>Caudal: Secondary (postpyramidal) cerebellar fissure.
**Tonsilla (tonsil)**	Rostral: Secondary (postpyramidal) cerebellar fissure;<br>Caudal: Posterolateral fissure.
**Flocculus (flocculus)**	Rostral: Posterolateral fissure;<br>Caudal: Inferior medullary velum.
**VENTRICULAR SYSTEM**	
**Lateral ventricles (LVc_total)**	Ventricular system proximal to the foramina of Monroe.
**Frontal horn (LVc_frontal_horn)**	Caudal: Coronal plane through the foramina of Monroe;<br>Ventral: Foramina of Monroe.
**Body (LVc_body)**	Rostral: Coronal plane through the foramina of Monroe;

	Caudal: Coronal plane through the caudal pole of the thalamus.
**Atrium (LVc_atrium)**	Rostral/ventral: Coronal plane through the caudal pole of the thalamus;<br>Caudal: Coronal plane through the rostral tip of the calcar avis;
**Occipital horn (LVc_occipital_horn)**	Rostral: Coronal plane through the rostral tip of the calcar avis.
**Temporal horn (LVc_temporal_horn)**	Caudal: Coronal plane through the caudal pole of the thalamus.
**Third ventricle (third_ventricle_total)**	Ventricular system distal to the foramina of Monro and proximal to the cerebral aqueduct.
**Fourth ventricle (fourth_ventricle_total)**	Ventricular system distal to the cerebral aqueduct and proximal to the foramina of Magendie/Luschkae.
**Apex (fourth_ventricle_apex)**	Caudal: Axial plane through the rostral tip of the limiting sulcus.
**Lateral recess (fourth_ventricle_lateral_recess)**	Medial: Sagittal plane through the limiting sulcus.
**Obex (fourth_ventricle_obex)**	Rostral: Axial plane through the caudal tip of the limiting sulcus.
**Fastigium (fourth_ventricle_fastigium)**	Basal: Coronal plane through the rostral tip of the cerebellar nodule.

Appendix 3: Definition of Arterial Units

Abbreviation	Vessel Name	Definition
**carotid**	Internal Carotid Artery	The paired internal carotid arteries (ICA) originate at the common carotid bifurcation, course upward through the carotid canal into the cranial cavity and can be classified into seven segments based on Bouthillier's classification.
**C1**	Internal Carotid Artery – Cervical Segment (C1)	The C1, also known as the cervical segment of the ICA, originates at the carotid bifurcation, courses upward in the carotid sheath and terminates at the external orifice of the carotid canal. The cervical ICA segment has no angiographically identifiable normal branches.
**C2**	Internal Carotid Artery (C2)	The C2, also known as the petrous segment of the ICA, originates at the external orifice of the carotid canal. It shows a vertical segment, a genu and a horizontal segment before its termination at the petrous apex above the foramen lacerum.
**caroticotympanic**	Caroticotympanic Artery	The caroticotympanic artery usually arises from the petrous segment of the ICA near its genu. It passes superiorly through the stapes to supply the middle ear cavity and oftentimes anastomoses with the external carotid artery.
**vidian**	Vidian Artery	The vidian artery usually arises from the external carotid artery but may originate from the horizontal part of the petrous ICA. When originating from the ICA, the vidian artery passes through the foramen lacerum and oftentimes anastomoses with the external carotid artery.
**C3**	Internal Carotid Artery – Laceral Segment (C3)	The C3, also known as the laceral segment of the ICA, originates where the ICA exits the petrous carotid canal. The C3 courses above the foramen lacerum and terminates at the petrolingual ligament. The laceral ICA segment has no angiographically identifiable normal branches.
**C4**	Internal Carotid Artery – Cavernous Segment (C4)	The C4, also known as the cavernous segment of the ICA, originates at the superior margin of the petrolingual ligament and terminates at the proximal dural ring. It has a posterior ascending portion, a longer horizontal segment and a short anterior vertical portion as it courses through the cavernous sinus.
**MHT**	Meningohypophyseal Trunk	The meningohypophyseal trunk, also known as the posterior trunk, arises from the superior aspect of the posterior genu of the cavernous ICA segment. It gives off multiple branches, named the inferior hypophyseal artery, the marginal tentorial artery and clival branches.

**ILT**	Inferolateral Trunk	The inferolateral trunk, sometimes also called the lateral trunk, originates from the horizontal cavernous ICA segment, providing arterial branches to the intracavernous cranial nerves and dura of the cavernous sinus.
**capsular**	Capsular Arteries	Capsular arteries are small and inconsistently found branches that originate from the cavernous ICA segment, supplying the pituitary gland.
**C5**	Internal Carotid Artery – Clinoid Segment (C5)	The wedge-shaped C5, also known as the clinoid segment of the ICA, begins at the proximal dural ring and ends at the distal dural ring, where the ICA enters the subarachnoid space. The clinoid segment has no angiographically identifiable normal branches, although the opthalmic artery may arise from this segment in rare instances.
**C6**	Internal Carotid Artery – Ophthalmic Segment (C6)	The C6, also known as the ophthalmic segment of the ICA, begins at the distal dural ring, courses posteriorly and slightly superiorly, terminating just proximal to the origin of the posterior communicating artery.
**ophthalmic**	Ophthalmic Artery	The ophthalmic artery arises medial to the anterior clinoid processes as the ICA exits the cavernous sinus. It delves anteriorly to enter the optic canal, giving off ocular, orbital and extraorbital branches.
**SHA**	Superior Hypophyseal Arteries	One or more superior hypophyseal arteries arises from the posteromedial aspect of the C6 segment of the ICA, supplying the anterior pituitary lobe, pituitary stalk, optic nerve and chiasm.
**C7**	Internal Carotid Artery – Communicating Segment (C7)	The C7, also known as the communicating segment of the ICA, begins just proximal to the origin of the posterior communicating artery and ends at the ICA bifurcation.
**PCoA**	Posterior Communicating Artery	The posterior communicating artery arises from the posterior aspect of the communicating ICA segment, courses posteriorly and anastomoses with the posterior cerebral artery.
**premamillary**	Premamillary Arteries	As the posterior communicating artery courses posteriorly, it gives off several small branches, called the anterior thalamoperforating arteries or premamillary arteries. These arteries pass through the posterior perforated substance to supply part of the thalamus and walls of the third ventricle.
**AChA**	Anterior Choroidal Artery	The anterior choroidal artery arises from the posteromedial aspect of the supraclinoid ICA, shortly after the posterior communicating artery origin. From ist origin, the AChA courses posteriorly below the optic tract, superiomedial to the uncus, turning laterally through the crural cistern and finally entering the temporal horn through the choroidal fissure.

**ACA**	Anterior Cerebral Artery	The anterior cerebral artery originates at the ICA bifurcation, and gives rise to segments A1-A3 during its course.
**A1**	Anterior Cerebral Artery – Precommunicating Segment (A1)	The A1 segment of the ACA, also called the precommunicating segment, emerges at the ACA origin and extends horizontally to its junction with the anterior communicating artery.
**MLSA**	Medial Lenticulostriate Arteries	Medial lenticulostriate arteries include perforating branches that originate from the precommunicating ACA segment, coursing posterosuperiorly through the anterior perforated substance to supply important parts of the anterobasal brain.
**ACoA**	Anterior Communicating Artery	The anterior communicating artery arises from the anterior cerebral artery and acts as an anastomosis between the left and right anterior cerebral circulation. Additionally, it demarcates the junction between the A1 and A2 segments of the anterior cerebral artery.
**A2**	Anterior Cerebral Artery – Infracallosal Segment (A2)	The A2 segment of the ACA, also called the infracallosal segment, originates at the ACoA, curves upward within the interhemispheric fissure and terminates at the genu of the corpus callosum.
**A3**	Anterior Cerebral Artery – Supracallosal Segment (A3)	The A3 segment of the ACA, also called the supracallosal segment, extends around the genu of the corpus callosum passing posteriorly and includes the distal ACA and its cortical branches.
**RAH**	Recurrent Artery of Heubner	The recurrent artery of Heubner is the largest perforating branch of the ACA, arising from the A1, proximal A2 or the AcoA. It runs parallel to its parent artery, terminating dorsally and slightly laterally to the carotid bifurcation.
**pericallosal**	Pericallosal Artery	The pericallosal artery represents the continuation of the main ACA trunk. It courses posteriorly above the corpus callosum at a variable distance. Splenial branches of the pericallosal artery anastomose with corresponding branches from the posterior cerebral artery.
**callosomarginal**	Callosomarginal Artery	The callosomarginal artery is the largest branch of the pericallosal artery and is present in 50% of cases. It courses posteriorly and superiorly above the cingulate gyrus in or near the cingulate sulcus.
**orbitofrontal**	Orbitofrontal Artery	Orbital branches, the most prominent of which is referred to as the orbitofrontal artery, usually arise from the proximal A2 segment, supplying the orbital surface of the frontal lobe.
**frontopolar**	Frontopolar Artery	Frontal branches, the most prominent of which is referred to as the frontopolar artery, usually arise from the A2 segment of the ACA below the genu of

		the corpus callosum, extending anteriorly to the frontal pole.
**IFA**	Internal Frontal Arteries	Internal frontal arteries, usually divided in anterior, middle and posterior internal frontal arteries, are cortical branches of the ACA that supply the medial and lateral surfaces of the superior frontal gyrus as far posteriorly as the paracentral lobule.
**paracentral**	Paracentral Artery	The paracentral arteries are branches of the ACA that usually arises midway between the genu and splenium of the corpus callosum. It courses toward the paracentral lobule, where it supplies a portion of the premotor, motor, and somatosensory areas.
**parietal**	Parietal Arteries	Parietal arteries, usually divided in superior and inferior parietal arteries, are branches of the ACA that supply the medial and superior aspect of the parietal lobes, including the precuneus and the adjacent cuneus.
**callosal**	Callosal Arteries	Callosal arteries are direct branches of the ACA, providing blood to the corpus callosum.
**MCA**	Middle Cerebral Artery	The middle cerebral artery originates at the internal carotid artery bifurcation and gives rise to M1-M4 segments during its course as described in detail in the respective sections.
**M1**	Middle Cerebral Artery – M1 Segment	The M1 segment of the MCA, also called the horizontal segment, begins at the internal carotid artery bifurcation and terminates at the limen insulae, including both pre- and postbifurcation segments.
**M2**	Middle Cerebral Artery – M2 Segment	The M2 segment of the MCA begins at the genu where the MCA turns posterosuperiorly over the insula and terminates at the circular sulcus.
**M3**	Middle Cerebral Artery – M3 Segment	The M3 segment of the MCA begins at the circular sulcus, extends laterally through the sylvian fissure and divides into a certain number of major stem arteries. The M3 segment terminates at the lateral end of the sylvian fissure.
**M4**	Middle Cerebral Artery – M4 Segment	The M4 segment of the MCA begins at the cortical surface and includes all cortical branches as they curve over the frontal, temporal and parietal operculae to their distal tributaries.
**LLSA**	Lateral Lenticulostriate Arteries	The lateral lenticulostriate arteries are central perforating arteries that arise from the M1 segment and course superiorly through the anterior perforated substance to supply deep structures of the brain.
**ant_temporal**	Anterior Temporal Artery	The anterior temporal artery usually arises from the M1 segment, coursing anteriorly and inferiorly over the temporal tip, supplying blood to part of the anterior temporal lobe.

**M4_orbitofrontal**	Orbitofrontal Artery	The orbitofrontal artery is a cortical branch of the MCA, which courses anteriorly to supply the inferior surface of the frontal lobe.
**M4_prefrontal**	Prefrontal Artery	The prefrontal artery is a cortical branch of the MCA, which courses around the frontal operculum supplying much of the lateral aspect of the frontal lobe.
**M4_precentral**	Precentral Artery	The precentral artery, also called the prerolandic artery, is a cortical branch of the MCA, which courses superiorly across the posterior part of the frontal operculum, supplying the anterolateral part of the central lobe.
**M4_central**	Central Artery	The central artery, also called the rolandic artery, is a cortical branch of the MCA, which courses between the pre- and postcentral gyri, supplying blood to much of the the lateral surface of the central lobe.
**M4_ant_parietal**	Anterior Parietal Artery	The anterior parietal artery, also called postcentral artery, is a cortical branch of the MCA that follows the postcentral sulcus and usually dives into the intraparietal sulcus more distally.
**M4_post_parietal**	Posterior Parietal Artery	The posterior parietal artery is a cortical branch of the MCA that crosses onto the convexity at the posterior end of the lateral sulcus, supplying blood to much of the lateral side of the parietal lobe.
**M4_angular**	Angular Artery	The angular artery is a cortical branch of the MCA that leaves the lateral sulcus in its most posterosuperior portion and crosses Heschl`s gyrus to provide terminal branches to the lateral side of the parietal lobe.
**M4_temporooccipital**	Temporooccipital Artery	The temporooccipital artery is a cortical branch of the MCA that usually courses posteriorly in the superior temporal sulcus to supply the superior temporal gyrus and part of the lateral surface of the occipital lobe.
**M4_post_temporal**	Posterior Temporal Artery	The posterior temporal artery is a cortical branch of the MCA that usually traverses the superior and middle temporal gyri to follow the inferior temporal sulcus and supply part of the posterolateral part of the temporal lobe.
**M4_med_temporal**	Medial Temporal Artery	The medial temporal artery is a cortical branch of the MCA that crosses the middle part of the temporal lobe to supply this territory.
**M4_ant_temporal**	Anterior Temporal Artery	The anterior temporal artery is a cortical branch of the MCA that crosses the anterior part of the temporal lobe to supply part of the anteroinferior temporal lobe.

**M4_temporopolar**	Temporopolar Artery	The temporopolar artery is a cortical branch of the MCA that traverses the temporal pole to supply this territory.
**PCA**	Posterior Cerebral Artery	The posterior cerebral arteries arise from the terminal basilar bifurcation ventral to the midbrain and give rise to P1-P4 segments during its course as described in detail in the respective sections.
**P1**	Posterior Cerebral Artery – Precommunicating Segment (P1)	The P1 segment of the PCA, also called the precommunicating segment, originates at the basilar bifurcation, courses posterolaterally through the interpeduncular cistern and terminates at the junction with the PCoA.
**TPA**	Thalamoperforating Arteries	Posterior thalamoperforating arteries arise from the posterior or superior aspect of the P1 segment of the PCA. They pass through the interpeduncular fossa and the posterior perforated substance, entering the brain behind the mamillary bodies.
**TGA**	Thalamogeniculate Arteries	Thalamogeniculate arteries usually arise from the P2 or less commonly from the P3 segment of the PCA and oftentimes build anastomoses with the medial posterior choroidal arteries.
**peduncular**	Peduncular Arteries	Peduncular perforating arteries are small and inconsistently found branches that arise from the P2 segment of the PCA, passing directly into the cerebral peduncles.
**MPChA**	Medial Posterior Choroidal Artery	The medial posterior choroidal artery usually arises from the P2 or less commonly from the P1 segment of the PCA, coursing around the brainstem and finally entering the roof of the third ventricle. It further courses through the foramen of Monro to enter the choroid plexus of the lateral ventricle.
**LPChA**	Lateral Posterior Choroidal Artery	The lateral posterior choroidal artery usually arises from the P2 segment of the PCA or from any cortical branch of the PCA, passing laterally through the choroidal fissure to enter the choroid plexus of the temporal horn and atrium.
**P2**	Posterior Cerebral Artery – Ambient Segment (P2)	The P2 segment of the PCA, also called the ambient segment, originates at the PCA-PCoA junction and extends to the posterior aspect of the midbrain, coursing posteriorly within the ambient cistern.
**P3**	Posterior Cerebral Artery – Quadrigeminal Segment (P3)	The P3 segment of the PCA, also called the quadrigeminal segment, begins from the level of the quadrigeminal plate of the midbrain, courses through the quadrigeminal cistern and extends to the calcarine fissure.
**P4**	Posterior Cerebral Artery – Calcarine Segment (P4)	The P4 segment of the PCA, also called the calcarine segment, begins at the anteriormost aspect of the calcarine fissure and extends until the PCA termination.

**ant_temporal**	Anterior Temporal Artery	The anterior temporal artery is a cortical branch of the PCA that usually arises from the P2 segment and supplies much of the anterior inferior surface of the temporal lobe.
**post_temporal**	Posterior Temporal Artery	The posterior temporal artery is a cortical branch of the PCA that usually arises from the P2 segment and supplies much of the posterior inferior surface of the temporal lobe and adjacent occipital lobe.
**calcarine**	Calcarine Artery	The calcarine artery is a cortical branch of the PCA, that runs in the calcarine fissure supplying part of the medial occipital lobe.
**parietooccippital**	Parietooccipital Artery	The parietooccipital artery is one of the terminal cortical branches of the PCA that runs posteriorly in the parietooccipital sulcus along the medial surface of the occipital lobe.
**AITA**	Anterior Inferior Temporal Artery	The anterior inferior temporal artery is a cortical branch of the PCA, supplying the anterior and inferior portion of the temporal lobe.
**MITA**	Middle Inferior Temporal Artery	The middle inferior temporal artery is a cortical branch of the PCA, supplying the middle and inferior portion of the temporal lobe.
**PITA**	Posterior Inferior Temporal Artery	The posterior inferior temporal artery is a cortical branch of the PCA, supplying the posterior and inferior portion of the temporal lobe.
**splenial**	Splenial Artery	The splenial branches of the PCA usually originate from the P4 segment, course upward and supply part of the corpus callosum, anastomosing with their counterparts from the anterior cerebral artery.
**VA**	Vertebral Artery	The paired vertebral arteries typically arise from the subclavian arteries and course superiorly, merging intracranially with the contralateral VA to form the basilar artery.
**V1**	Vertebral Artery – Extraosseous Segment (V1)	The V1 segment of the vertebral artery, also called extraosseous segment, typically arises from the subclavian artery, coursing posterosuperiorly and terminates at the transverse foramen of the sixth cervical vertebra.
**V2**	Vertebral Artery – Foraminal Segment (V2)	The V2 segment of the vertebral artery, also called foraminal segment, originates as the artery enters the sixth cervical vertebra. From here, it ascends through the foramina of the C3-6 transverse processes, following an inverted L shaped course through C2, before running superiorly again through the C1 transverse foramina.
**V3**	Vertebral Artery – Extraspinal Segment (V3)	The V3 segment of the vertebral artery, also called extraspinal segment, begins as the vertebral artery exits from C1 and ends where it penetrates the dura.

**V4**	Vertebral Artery – Intradural Segment (V4)	The V4 segment of the vertebral artery, also called intradural segment, courses through the foramen magnum and terminates as both vertebral arteries join to form the basilar artery near the pontomedullary junction.
**meningeal_branches**	Meningeal Branches	Anterior and posterior meningeal branches arise from the distal extracranial vertebral artery (V2 and V3 segments) to supply part of the posterior fossa dura.
**post_spinal**	Posterior Spinal Arteries	The posterior spinal arteries arises from the distal vertebral arteries or posterior inferior cerebellar arteries, descending along the dorsal surface of the medulla and spinal cord.
**Ant_spinal**	Anterior Spinal Artery	The anterior spinal artery arises from the distal vertebral artery, giving off a number of small perforating branches that supply the anterior surface of the medulla, descending along the dorsal surface of the medulla and spinal cord.
**PICA**	Posterior Inferior Cerebellar Artery	The posterior inferior cerebellar artery arises from variable sites of the vertebral artery at the anterolateral aspect of the brainstem. It is the largest cerebellar artery and gives off multiple small perforating, choroidal and cortical branches.
**BA**	Basilar Artery	The basilar artery is formed near the pontomedullary junction by the confluence of the two vertebral arteries, extending superiorly to its terminal bifurcation where it divides into the two posterior cerebral arteries.
**AICA**	Anterior Inferior Cerebellar Artery	The anterior inferior cerebellar artery arises from the basilar artery, typically at the level of the pontomedullary junction, supplying a variable amount of the anterior and inferior surface of the cerebellum.
**SCA**	Superior Cerebellar Artery	The superior cerebellar artery arises from the terminal third of the basilar artery, passing laterally around the brainstem to supply part of the superior cerebellum.
**ECA**	External Carotid Artery	The external carotid artery originates from the common carotid artery at the mid-cervical level, giving off multiple branches to the head and neck as it ascends in the carotid sheath.
**superior_thyroid**	Superior Thyroid Artery	The superior thyroid artery is usually the first anterior branch of the ECA. It arised from the anterior wall of the ECA, coursing anteroinferiorly and slightly medially toward the apex of the thyroid gland.
**APA**	Ascending Pharyngeal Artery	The ascending pharyngeal artery is a small, posterior branch of the ECA, usually originating from the common carotid bifurcation or proximal ECA. It ascends between the internal carotid artery and the

		internal jugular vein, giving off pharyngeal, inferior tympanic, muscular and neuromeningeal branches during ist ascending course.
**LA**	Lingual Artery	The lingual artery arises from the anterior surface of the ECA, coursing superiorly and medial to the pharyngeal muscles before turning toward the oral cavity.
**FA**	Facial Artery	The facial artery is a major anterior branch of the ECA, originating just above the lingual artery. It courses superiorly over the mandible and cheek, giving off multiple branches to facial structures. It terminates as the angular artery near the medial canthus of the eye.
**OccA**	Occipital Artery	The occipital artery is a posterior branch of the ECA, coursing posterosuperiorly between the occipital bone and the first cervical vertebra. Ist branches supply the musculocutaneous structures of the posterior neck and scalp. Additionally the occpital artery provides meningeal rami to the posterior fossa.
**PAA**	Posterior Auricular Artery	The posterior auricular artery is a small branch that arises from the posterior aspect of the ECA just above the occipital artery origin. It courses posteropsuperiorly to supply the scalp, pinna, chorda tympani and external auditory canal.
**STA**	Superficial Temporal Artery	The ECA divides into the maxillary and superficial temporal arteries just inferior to the condyle. The STA runs behind the condyle, passing upward, supplying the anterior two thirds iof the scalp, part of the ear, face and the parotid gland.
**IMA**	Internal Maxillary Artery	The internal maxillary artery is one of the terminal branches of the ECA. It arises behind the neck of the mandible and passes obliquely forward and medially in the infratemporal fossa. It gives rise to numerous branches, including the middle meningeal artery, as it divides in the pterygopalatine fossa.
**MMA**	Middle Meningeal Artery	The middle meningeal artery is the largest and most proximal major branch of the internal maxillary artery. It courses superiorly to the foramen spinosum, where it enters the skull to supply the meninges and cavaria.
**AMA**	Accessory Meningeal Artery	The accessory meningeal artery is another small but important branch, which arises from the proximal IMA, coursing through the foramen ovale to supply meningeal and other intracranial structures.

Appendix 4: Definition of Venous Units

Abbreviation	Vessel Name	Definition
Sinuses		
**SSS**	Superior Sagittal Sinus	The superior sagittal sinus originates near the crista galli, arcing posteriorly at the junction of the falx cerebri and terminates by joining with the straight sinus to form the torcular Herophili at the internal occipital protuberance.
**ISS**	Inferior Sagittal Sinus	The inferior sagittal sinus begins at the junction of the anterior and middle thirds of the falx cerebri and courses posteriorly in the inferior free edge of the falx. It ends at the falcotentorial apex by joining with the vein of Galen to form the straight sinus.
**StrS**	Straight Sinus	The straight sinus is formed by the confluence of the inferior sagittal sinus and the vein of Galen. It runs posteroinferiorly and terminates at the internal occipital protuberance by becoming the transverse sinus.
**torcular**	Torcular	The torcular Herophili, also called the confluence of sinuses, is formed at the internal occipital protuberance by the union of the superior sagittal sinus, the straight sinus and transverse sinuses.
**TS**	Transverse Sinus	The transverse sinuses, also known as the lateral sinuses, begin at the internal occipital protuberance and course anterolaterally to the petrous temporal bones, where they become the sigmoid sinuses.
**MTS**	Medial Tentorial Sinus	The medial tentorial sinuses represent variable venous channels that arise by the convergence of draining veins along the tentorium, the basal surface of the temporal and occipital lobes as well as the cerebellum. The medial tentorial sinuses drain medially into the transverse sinus.
**LTS**	Lateral Tentorial Sinus	The lateral tentorial sinuses represent variable venous channels that arise by the convergence of draining veins along the tentorium, the basal surface of the temporal and occipital lobes as well as the cerebellum. The lateral tentorial sinuses drain laterally into the transverse sinus.
**SS**	Sigmoid Sinus	The sigmoid sinuses begin where the transverse sinuses leave the tentorial margin, continue anteroinferiorly and drain into the jugular bulbs.
**CS**	Cavernous Sinus	The cavernous sinuses lie on either side of the sphenoid body and are formed by multiple venous channels, extending from the superior orbital fissure anteriorly to the petrous apex posteriorly, emptying into the superior and inferior petrosal sinuses.
**SPS**	Superior Petrosal Sinus	The superior petrosal sinuses extend from the cavernous sinus to the transverse sinuses, running

		along the attachment of the tentorium cerebelli to the dorsal ridge of the petrous bones.
**IPS**	Inferior Petrosal Sinus	The inferior petrosal sinuses lie in a groove between the petrous apices and the clivus, usually terminating by draining into the jugular bulbs.
**SpPS**	Sphenoparietal Sinus	The sphenoparietal sinuses are the anteroinferior continuation of the superficial middle cerebral veins. They follow the curve of the lesser sphenoid ala and usually drain into the cavernous sinuses.
**OS**	Occipital Sinus	The occipital sinus begins at the posterior margin of the foramen magnum and passes superiorly, draining into the torcular Herophili.
Superficial venous system		
Superior Sagittal Group		
**frontopolar**	Frontopolar Vein	The frontopolar veins are cortical veins, draining the frontopolar area of the frontal lobes, emptying into the superior sagittal sinus.
**ant_frontal**	Anterior Frontal Vein	The anterior frontal veins are cortical veins, draining the anterior portions of the frontal lobes' lateral surfaces, ultimately emptying into the superior sagittal sinus.
**mid_frontal**	Middle Frontal Vein	The middle frontal veins are cortical veins that drain the middle portions of the frontal lobes' lateral surfaces and ultimately empty into the superior sagittal sinus.
**post_frontal**	Posterior Frontal Vein	The posterior frontal veins are cortical veins that drain the posterior portions of the frontal lobes' lateral surfaces and ultimately empty into the superior sagittal sinus.
**precentral**	Precentral Vein	The precentral veins are cortical veins that drain the precentral portions of the central lobes' lateral surfaces and ultimately empty into the superior sagittal sinus.
**central**	Central Vein	The central veins are cortical veins that drain the central portions of the central lobes' lateral surfaces and ultimately empty into the superior sagittal sinus.
**postcentral**	Postcentral Vein	The postcentral veins are cortical veins that drain the postcentral portions of the central lobes' lateral surfaces and ultimately empty into the superior sagittal sinus.
**ant_parietal**	Anterior Parietal Vein	The anterior parietal veins are cortical veins that drain the anterior portions of the parietal lobes' lateral surfaces and ultimately empty into the superior sagittal sinus.
**post_parietal**	Posterior Parietal Vein	The posterior parietal veins are cortical veins that drain the posterior portions of the parietal lobes' lateral surfaces and ultimately empty into the superior sagittal sinus.

**occipital**	Occipital Vein	The occipital veins are cortical veins that drain the occipital lobes' lateral surfaces and ultimately empty into the superior sagittal sinus.
**trolard**	Vein of Trolard	The superior anastomotic veins, also called the veins of Trolard, course posterosuperiorly from the sylvian fissure over the mid-hemispheric convexity, connecting the superficial sylvian veins to the superior sagittal sinus.
**anteromedial_frontal**	Anteromedial Frontal Vein	The anteromedial frontal veins are cortical veins that drain the anterior portions of the frontal lobes' medial surfaces and ultimately empty into the superior sagittal sinus.
**centromedial_frontal**	Centromedial Frontal Vein	The centromedial frontal veins are cortical veins that drain the middle portions of the frontal lobes' medial surfaces and ultimately empty into the superior sagittal sinus.
**posteromedial_frontal**	Posteromedial Frontal Vein	The posteromedial frontal veins are cortical veins that drain the posterior portions of the frontal lobes' medial surfaces and ultimately empty into the superior sagittal sinus.
**paracentral**	Paracentral Vein	The paracentral veins are cortical veins of the central lobes, draining the central lobes' medial surfaces and ultimately emptying into the superior sagittal sinus.
**anteromedial_parietal**	Anteromedial Parietal Vein	The anteromedial parietal veins are cortical that drain the anterior portions of the parietal lobes' medial surfaces and ultimately empty into the superior sagittal sinus.
**posteromedial_parietal**	Posteromedial Parietal Vein	The posteromedial parietal veins are cortical veins that drain the posterior portions of the pariteal lobes' medial surfaces and ultimately empty into the superior sagittal sinus.
**post_calcarine**	Posterior Calcarine Vein	The posterior calcarine veins are cortical veins that drain the occipital lobes' medial surfaces and ultimately empty into the superior sagittal sinus.
Sphenoidal group		
**SMCV**	Superficial Middle Cerebral Vein	The superficial middle cerebral veins, also called the superficial sylvian veins, collect a number of small tributaries that drain the opercular areas and lateral sulcus. The SMCVs then curve around the temporal tip before passing medially into the cavernous or sphenoparietal sinuses.
**frontosylvian**	Frontosylvian Veins	The frontosylvian veins represent the frontal group of superficial middle cerebral veins, collecting tributaries from the lateral and opercular frontal lobes.
**parietosylvian**	Parietosylvian Veins	The parietosylvian veins represent the parietal group of superficial middle cerebral veins, collecting tributaries from the lateral and opercular parietal lobes.

**temporosylvian**	Temporosylvian Veins	The temporosylvian veins represent the temporal group of superficial middle cerebral veins, collecting tributaries from the lateral and opercular temporal lobes.
Tentorial group		
**anterior_temporal**	Anterior Temporal Vein	The anterior temporal veins collect tributaries from the anterior portion of the lateral surface of the temporal lobes and empty into the tentorial sinuses.
**middle_temporal**	Middle Temporal Vein	The middle temporal veins collect tributaries from the middle portion of the lateral surface of the temporal lobes and empty into the tentorial sinuses.
**posterior_temporal**	Posterior Temporal Vein	The posterior temporal veins collect tributaries from the posterior portion of the lateral surface of the temporal lobes and empty into the tentorial sinuses.
**anterior_temporobasal**	Anterior Temporobasal Vein	The anterior temporobasal veins collect tributaries from the anterior portion of the basal surface of the temporal lobes and empty into the tentorial sinuses.
**middle_temporobasal**	Middle Temporobasal Vein	The middle temporobasal veins collect tributaries from the middle portion of the basal surface of the temporal lobes and empty into the tentorial sinuses.
**posterior_temporobasal**	Posterior Temporobasal Vein	The posterior temporobasal veins collect tributaries from the posterior portion of the basal surface of the temporal lobes and empty into the tentorial sinuses.
**occipital_basal**	Occipital Basal Vein	The occipital basal veins collect tributaries from the basal surface of the occipital lobes and empty into the tentorial sinuses.
**labbe**	Vein of Labbé	The inferior anastomotic vein, also called the vein of Labbé, courses over the temporal lobe along the occipitotemporal sulcus, connecting the superficial sylvian vein with the transverse sinuses.
Deep venous system		
**paraterminal**	Paraterminal Vein	The paraterminal vein drains the area adjacent to the lamina terminalis and drains posteriorly toward the anterior cerebral vein, which empties into the anterior end of the basal vein.
**post_frontoorbital**	Posterior Frontoorbital Vein	The posterior frontoorbital veins drain the posterior frontoorbital area and drain posteriorly toward the anterior cerebral vein, which empties into the anterior end of the basal vein.
**olfactory**	Olfactory Vein	The olfactory veins drain the area adjacent to the olfactory gyrus and drain posteriorly toward the anterior cerebral vein, which empties into the anterior end of the basal vein.
**ant_pericallosal**	Anterior Pericallosal Vein	The anterior parts of the cingulate gyrus and corpus callosum are drained by the anterior pericallosal veins, which may join the inferior sagittal sinus or the anterior cerebral vein.

**post_pericallosal**	Posterior Pericallosal Vein	The posterior part of the cingulate gyrus is drained by the posterior pericallosal vein, which drains into the great or internal cerebral veins in the quadrigeminal cistern.
**medial_temporal**	Medial Temporal Veins	The medial part of the parahippocampal gyrus and uncus are drained by the uncal, anterior hippocampal, and medial temporal veins, which pass medially to empty into the basal vein in the crural and ambient cisterns.
**ant_calcarine**	Anterior Calcarine Vein	The area adjoining the isthmus of the cingulate gyrus and the area surrounding the anterior part of the calcarine fissure is drained by anterior calcarine veins, which cross the quadrigeminal cistern to reach the great vein or its tributaries.
Internal cerebral vein		
**internal_cerebral**	Internal Cerebral Vein	The paired internal cerebral veins originate just behind the foramen of Monro and course posteriorly within the velum interpositum. The union of the paired veins to form the great vein may be located above or posterior to the pineal body and inferior or posterior to the splenium.
**septal**	Septal Vein	The anterior and posterior septal veins originate at the lateral aspect of the frontal horns, pass medially under the corpus callosum, turning backward along the septum pellucidum. Finally, the septal veins enter the internal cerebral veins behind the foramen of Monro.
**medial_atrial**	Medial Atrial Vein	The medial atrial veins are ventricular veins that drain the medial portion of the atrium, emptying into the internal cerebral veins.
**caudate**	Caudate Vein	The anterior and posterior caudate veins are two small and variable veins that drain the caudate nucleus and empty into the thalamostriate or internal cerebral veins or its tributaries.
**thalamostriate**	Thalamostriate Vein	The thalamostriate veins arise from tributaries that converge between the caudate nucleus and thalamus. The thalamostriate veins are directed medially, forming the internal cerebral veins by joining with the septal veins.
**thalamocaudate**	Thalamocaudate Vein	The thalamocaudate veins course medially across the caudate nucleus and thalamus behind the posterior extension of the thalamostriate vein and terminate in the internal cerebral vein
**thalamic**	Thalamic Vein	Thalamic veins, including the anterior thalamic vein, superior thalamic vein, anterior superficial thalamic vein, and superior superficial thalamic vein drain the thalamic area and empty into the internal cerebral veins.
Basal vein		

**basal**	Basal Vein	The basal vein, also called the basal vein of Rosenthal, is formed near the limen insulae by the juncture of the deep middle cerebral veins and the anterior cerebral veins. It courses posteriorly through the perimesencephalic cisterns, receiving multiple small tributaries, and empties into the great vein of Galen.
**ant_cerebral**	Anterior Cerebral Vein	The anterior cerebral veins run in parallel to the anterior cerebral artery in the interhemispheric fissure. On its posteriorly directed course, it collects small tributaries and empties into the basal vein of Rosenthal.
**DMCV**	Deep Middle Cerebral Vein	The deep middle cerebral veins, also called the deep sylvian veins, drain the cortical surfaces adjacent to the deeper portion of the sylvian fissures and usually empty into the basal vein. Its tributaries drain the inferior frontal and temporal lobes such as the insula and parahippocampal gyri.
**inf_ventricular**	Inferior Ventricular Vein	The hippocampal and inferior ventricular veins, namely the anterior hippocampal veins, anterior longitudinal hippocampal veins, transverse longitudinal hippocampal veins, posterior longitudinal hippocampal veins, inferior ventricular veins, amygdala veins and inferior choroidal veins drain the area adjacent to the hippocampus and the inferior surface of the temporal horn of the lateral ventricles, emptying into the basal vein.
**lat_atrial**	Lateral Atrial Vein	Lateral atrial veins are ventricular veins that drain the lateral portion of the atrium, emptying into the basal veins.
Posterior fossa veins		
**PCV**	Precentral Cerebellar Vein	The precentral cerebellar vein is a single midline vein that originates in the fissure between the vermian lingula and the central lobule. It courses superiorly and terminates behind the inferior colliculi by draining into the vein of Galen.
**SVV**	Superior Vermian Vein	The superior vermian vein originates near the vermian declive, curves upward and forward, terminating by entering the vein of Galen just anterior to the precentral cerebellar vein.
**APMV**	Anterior Pontomesencephalic Vein	The anterior pontomesencephalic vein, also called the anterior pontomesencephalic venous plexus, consists of many small veins that are closely applied to the pons and mesencephalon. It drains blood from the small pontine and mesencephalic veins, usually curves into the interpeduncular fossa and drains into the basal vein of Rosenthal. The lateral mesencephalic and pontine veins are also included in this group.
**petrosal**	Petrosal Vein	The petrosal vein is a short trunk that forms near the cerebellopontine angle, collecting numerous tributaries

		from the cerebellum, pons and medulla. It courses anterolaterally to enter the superior petrosal sinus.
**IVV**	Inferior Vermian Vein	The inferior vermian veins are paired paramedian vessels that curve posterosuperiorly along the inferior vermis, receiving blood from hemispheric veins and usually terminate within the tentorial sinus.
**PMVP**	Perimedullary Venous Plexus	The perimedullary venous plexus entails the anterior and lateral medullary veins. These veins are usually continuous with the lateral pontine veins or anterior pontomesencephalic veins, draining superiorly into the basal vein of Rosenthal.
**hemispheric**	Hemispheric Veins	The hemispheric cerebellar veins run along the cerebellar surface, receiving blood from the cerebellar hemispheric parenchyma and usually drain in the petrosal and vermian veins.
**galen**	Vein of Galen	The great cerebral vein, also called the vein of Galen, is formed by the confluence of the paired internal cerebral veins, the basal veins, the precentral cerebellar vein and the superior vermian veins. The great cerebral vein empties into the straight sinus.

Appendix 5: Ontogenetic Mapping of Parenchymal Units

The individual topographic parcellation units are listed with their ontogenetic affiliation. *Abbreviations: D = diencephalon; DPa = dorsal pallium; H = hypothalamus; LPa = lateral pallium; M = mesencephalon; MPa = medial pallium; P = prosencephalon; Pa = pallium; PM = Pontomedullary rhombecephalon; R = rhombencephalon; SPa = Subpallium; VPa = ventral pallium.*

Abbreviation	Gyrus Name	Phase 1	Phase 2	Phase 3
	**Cerebral gyral segments**			
frontal_pole	Frontal pole	P	Pa	DPa
F1total	Superior frontal gyrus	P	Pa	DPa
F2total	Middle frontal gyrus	P	Pa	DPa
F3orbital	Inferior frontal gyrus, orbital part	P	Pa	DPa
F3triangular	Inferior frontal gyrus, triangular part	P	Pa	DPa
F3opercular	Inferior frontal gyrus, opercular part	P	Pa	DPa
ORBant	Anterior orbital gyrus	P	Pa	DPa
ORBmed	Medial orbital gyrus	P	Pa	DPa
ORBlat	Lateral orbital gyrus	P	Pa	DPa
ORBpost	Posterior orbital gyrus	P	Pa	LPa
rectus	Gyrus rectus	P	Pa	DPa
rostral	Rostral gyrus	P	Pa	DPa
subcallosal	Subcallosal area	P	Pa	DPa
precentral	Precentral gyrus	P	Pa	DPa
postcentral	Postcentral gyrus	P	Pa	DPa
paracentral	Paracentral lobule	P	Pa	DPa
subcentral	Subcentral gyrus	P	Pa	DPa
SPL	Superior parietal lobule	P	Pa	DPa
SMG	Supramarginal gyrus	P	Pa	DPa
ANG	Angular gyrus	P	Pa	DPa
precuneus	Precuneus	P	Pa	DPa
cuneus	Cuneus	P	Pa	DPa
O1	Superior occipital gyrus	P	Pa	DPa
O2	Middle occipital gyrus	P	Pa	DPa
O3	Inferior occipital gyrus	P	Pa	DPa
occipital_pole	Occipital pole	P	Pa	DPa
lingual	Lingual gyrus	P	Pa	DPa
fusiform	Fusiform gyrus	P	Pa	DPa
T1	Superior temporal gyrus	P	Pa	DPa

T2	Middle temporal gyrus	P	Pa	DPa
T3	Inferior temporal gyrus	P	Pa	DPa
planum_temporale	Planum temporale	P	Pa	DPa
planum_polare	Planum polare	P	Pa	LPa
temporal_pole	Temporal pole	P	Pa	DPa
INSshort	Short insular gyri	P	Pa	LPa
INSlong	Long insular gyri	P	Pa	DPa
PHG	Parahippocampal gyrus	P	Pa	MPa
amygdala	Amygdala	P	Pa	VPa
Hippocampus	Hippocampus	P	Pa	MPa
CINant	Cingulate anterior	P	Pa	DPa
CINmid	Cingulate middle	P	Pa	DPa
CINpost	Cingulate posterior	P	Pa	DPa
	**Central supratentorial structures**			
caudate	Caudate nucleus	P	SP	Striatum
putamen	Putamen	P	SP	Striatum
globus_pallidus	Globus pallidum	P	SP	Pallidum
hypothalamus	Hypothalamus	P	SP	H
thalamus	Thalamus	P	D	D
	**Brainstem**			
mesencephalon	Mesencephalon	M	M	M
pons	Pons	R	PM	PM
medulla_oblongata	Medulla oblongata	R	PM	PM
	**Vermian lobules**			
central_lobule	Central	R	Pre-Fissura prima	Pre-preculminate fissure
culmen	Culmen	R	Pre-Fissura prima	Post-preculminate fissure
declive	Declive	R	Post-Fissura prima	Pre-prepyramidal fissure
folium	Folium	R	Post-Fissura prima	Pre-prepyramidal fissure

tuber	Tuber	R	Post-Fissura prima	Pre-prepyramidal fissure
pyramid	Pyramid	R	Post-Fissura prima	Post-prepyramidal fissure
uvula	Uvula	R	Post-Fissura prima	Post-prepyramidal fissure
nodule	Nodule		Post-Fissura prima	Post-prepyramidal fissure
	**Cerebellar hemispheric lobules**			
ala_lobuli_centralis	Ala lobuli centralis	R	Pre-Fissura prima	Pre-preculminate fissure
anterior_quadrangular	Anterior quadrangular lobule	R	Pre-Fissura prima	Post-prepyramidal fissure
post_quadrangular	Posterior quadrangular lobule	R	Post-Fissura prima	Pre-prepyramidal fissure
superior_semilunar	Superior semilunar lobule	R	Post-Fissura prima	Pre-prepyramidal fissure
inferior_semilunar	Inferior semilunar / gracile lobule	R	Post-Fissura prima	Pre-prepyramidal fissure
biventral_lobule	Biventer lobule	R	Post-Fissura prima	Post-prepyramidal fissure
tonsil	Tonsilla	R	Post-Fissura prima	Post-prepyramidal fissure
flocculus	Flocculus	R	Post-Fissura prima	Post-prepyramidal fissure

Appendix 6: Ontogenetic Mapping of Arterial Units

Abbreviations: ICA: Internal Carotid Artery; LNS: Longitudinal Neural System; LOA: Lateral Olfactory Artery; LSA: Lateral Striate Artery; MOA: Medial Olfactory Artery.

Abbreviation	Vessel Name	Phase 1	Phase 2	Phase 3
**carotid**	Internal Carotid Artery	ICA	ICA	ICA
**C1**	Internal Carotid Artery – Cervical Segment (C1)	ICA	ICA	ICA
**C2**	Internal Carotid Artery (C2)	ICA	ICA	ICA
**caroticotympanic**	Caroticotympanic Artery	ICA	ICA	ICA
**vidian**	Vidian Artery	ICA	ICA	ICA
**C3**	Internal Carotid Artery – Laceral Segment (C3)	ICA	ICA	ICA
**C4**	Internal Carotid Artery – Cavernous Segment (C4)	ICA	ICA	ICA
**MHT**	Meningohypophyseal Trunk	ICA	ICA	ICA
**ILT**	Inferolateral Trunk	ICA	ICA	ICA
**capsular**	Capsular Arteries	ICA	ICA	ICA
**C5**	Internal Carotid Artery – Clinoid Segment (C5)	ICA	ICA	ICA
**C6**	Internal Carotid Artery – Ophthalmic Segment (C6)	ICA	ICA	ICA
**ophthalmic**	Ophthalmic Artery	ICA	ICA	ICA
**SHA**	Superior Hypophyseal Arteries	ICA	ICA	ICA
**C7**	Internal Carotid Artery – Communicating Segment (C7)	ICA	ICA	ICA
**PCoA**	Posterior Communicating Artery	Caudal Ramus	Caudal Ramus	Caudal Ramus
**premamillary**	Premamillary Arteries	Caudal Ramus	Caudal Ramus	Caudal Ramus
**AchA**	Anterior Choroidal Artery	Cranial Ramus	LOA	LSA
**ACA**	Anterior Cerebral Artery	Cranial Ramus	MOA	MOA
**A1**	Anterior Cerebral Artery – Precommunicating Segment (A1)	Cranial Ramus	MOA	MOA
**mLSA**	Medial Lenticulostriate Arteries	Cranial Ramus	MOA	LSA

**ACoA**	Anterior Communicating Artery	Cranial Ramus	MOA	MOA
**A2**	Anterior Cerebral Artery – Infracallosal Segment (A2)	Cranial Ramus	MOA	MOA
**A3**	Anterior Cerebral Artery – Supracallosal Segment (A3)	Cranial Ramus	MOA	MOA
**RAH**	Recurrent Artery of Heubner	Cranial Ramus	LOA	LSA
**callosomarginal**	Callosomarginal Artery	Cranial Ramus	MOA	MOA
**orbitofrontal**	Orbitofrontal Artery	Cranial Ramus	MOA	MOA
**ACA_frontopolar**	Frontopolar Artery	Cranial Ramus	MOA	MOA
**IFA**	Internal Frontal Arteries	Cranial Ramus	MOA	MOA
**ACA_paracentral**	Paracentral Artery	Cranial Ramus	MOA	MOA
**parietal**	Parietal Arteries	Cranial Ramus	MOA	MOA
**callosal**	Callosal Arteries	Cranial Ramus	MOA	MOA
**MCA**	Middle Cerebral Artery	Cranial Ramus	LOA	LSA
**M1**	Middle Cerebral Artery – M1 Segment	Cranial Ramus	LOA	LSA
**M2**	Middle Cerebral Artery – M2 Segment	Cranial Ramus	LOA	LSA
**M3**	Middle Cerebral Artery – M3 Segment	Cranial Ramus	LOA	LSA
**M4**	Middle Cerebral Artery – M4 Segment	Cranial Ramus	LOA	LSA
**LLSA**	Lateral Lenticulostriate Arteries	Cranial Ramus	LOA	LSA
**MCA_ant_temporal**	Anterior Temporal Artery	Cranial Ramus	LOA	LSA
**M4_orbitofrontal**	Orbitofrontal Artery	Cranial Ramus	LOA	LSA
**M4_prefrontal**	Prefrontal Artery	Cranial Ramus	LOA	LSA
**M4_precentral**	Precentral Artery	Cranial Ramus	LOA	LSA

**M4_central**	Central Artery	Cranial Ramus	LOA	LSA
**M4_ant_parietal**	Anterior Parietal Artery	Cranial Ramus	LOA	LSA
**M4_post_parietal**	Posterior Parietal Artery	Cranial Ramus	LOA	LSA
**M4_angular**	Angular Artery	Cranial Ramus	LOA	LSA
**M4_temporooccipital**	Temporooccipital Artery	Cranial Ramus	LOA	LSA
**M4_post_temporal**	Posterior Temporal Artery	Cranial Ramus	LOA	LSA
**M4_med_temporal**	Medial Temporal Artery	Cranial Ramus	LOA	LSA
**M4_ant_temporal**	Anterior Temporal Artery	Cranial Ramus	LOA	LSA
**M4_temporopolar**	Temporopolar Artery	Cranial Ramus	LOA	LSA
**PCA**	Posterior Cerebral Artery	Caudal Ramus	Caudal Ramus	Tectal Branch
**P1**	Posterior Cerebral Artery – Precommunicating Segment (P1)	Caudal Ramus	Caudal Ramus	Tectal Branch
**TPA**	Thalamoperforating Arteries	Caudal Ramus	Caudal Ramus	Tectal Branch
**TGA**	Thalamogeniculate Arteries	Caudal Ramus	Caudal Ramus	Tectal Branch
**PCA_peduncular**	Peduncular Arteries	Caudal Ramus	Caudal Ramus	Tectal Branch
**MPChA**	Medial Posterior Choroidal Artery	Caudal Ramus	Diencephalic Branch	Diencephalic Branch
**LPChA**	Lateral Posterior Choroidal Artery	Caudal Ramus	Diencephalic Branch	Diencephalic Branch
**P2**	Posterior Cerebral Artery – Ambient Segment (P2)	Caudal Ramus	Caudal Ramus	Tectal Branch
**P3**	Posterior Cerebral Artery – Quadrigeminal Segment (P3)	Caudal Ramus	Caudal Ramus	Tectal Branch
**P4**	Posterior Cerebral Artery – Calcarine Segment (P4)	Caudal Ramus	Caudal Ramus	Tectal Branch
**PCA_ant_temporal**	Anterior Temporal Artery	Caudal Ramus	Caudal Ramus	Tectal Branch
**PCA_post_temporal**	Posterior Temporal Artery	Caudal Ramus	Caudal Ramus	Tectal Branch

**calcarine**	Calcarine Artery	Caudal Ramus	Caudal Ramus	Tectal Branch
**parietooccippital**	Parietooccipital Artery	Caudal Ramus	Caudal Ramus	Tectal Branch
**AITA**	Anterior Inferior Temporal Artery	Caudal Ramus	Caudal Ramus	Tectal Branch
**MITA**	Middle Inferior Temporal Artery	Caudal Ramus	Caudal Ramus	Tectal Branch
**PITA**	Posterior Inferior Temporal Artery	Caudal Ramus	Caudal Ramus	Tectal Branch
**splenial**	Splenial Artery	Caudal Ramus	Caudal Ramus	Tectal Branch
**VA**	Vertebral Artery	LNS	LNS	Vertebrobasilar
**V1**	Vertebral Artery – Extraosseous Segment (V1)	LNS	LNS	Vertebrobasilar
**V2**	Vertebral Artery – Foraminal Segment (V2)	LNS	LNS	Vertebrobasilar
**V3**	Vertebral Artery – Extraspinal Segment (V3)	LNS	LNS	Vertebrobasilar
**V4**	Vertebral Artery – Intradural Segment (V4)	LNS	LNS	Vertebrobasilar
**meningeal_branches**	Meningeal Branches	LNS	LNS	Vertebrobasilar
**post_spinal**	Posterior Spinal Arteries	LNS	LNS	Vertebrobasilar
**ant_spinal**	Anterior Spinal Artery	LNS	LNS	Vertebrobasilar
**PICA**	Posterior Inferior Cerebellar Artery	LNS	LNS	Vertebrobasilar
**BA**	Basilar Artery	Caudal Ramus	Caudal Ramus	Caudal Ramus
**AICA**	Anterior Inferior Cerebellar Artery	Caudal Ramus	Caudal Ramus	Caudal Ramus
**SCA**	Superior Cerebellar Artery	Caudal Ramus	Caudal Ramus	Caudal Ramus

Appendix 7: Ontogenetic Mapping of Venous Units

Abbreviation	Vessel Name	Phase 1	Phase 2	Phase 3
Sinuses				
**SSS**	Superior Sagittal Sinus	Anterior dural plexus	Plexus sagittalis	Superior Sagittal Sinus
**ISS**	Inferior Sagittal Sinus	Anterior dural plexus	Plexus sagittalis	Superior Sagittal Sinus
**StrS**	Straight Sinus	Anterior dural plexus	Plexus sagittalis	Straight sinus
**torcula**	Torcular	Anterior/Middle dural plexus	Tentorial plexus	Tentorial plexus
**TS**	Transverse Sinus	Middle/Posterior dural plexus	Transverse sinus	Transverse sinus
**SS**	Sigmoid Sinus	Middle/Posterior dural plexus	Transverse sinus	Sigmoid sinus
**MTS**	Medial Tentorial Sinus	Anterior/Middle dural plexus	Tentorial plexus	Tentorial plexus
**LTS**	Lateral Tentorial Sinus	Anterior/Middle dural plexus	Tentorial plexus	Tentorial plexus
**CS**	Cavernous Sinus	Primary head-vein	Cavernous sinus	Cavernous sinus
**SPS**	Superior Petrosal Sinus	Primary head-vein	Primary head-vein	Superior petrosal sinus
**IPS**	Inferior Petrosal Sinus	Primary head-vein	Primary head-vein	Inferior petrosal sinus
**SpPS**	Sphenoparietal Sinus	Primary head-vein	Cavernous sinus	Cavernous sinus
**OS**	Occipital Sinus	Posterior dural plexus	Posterior dural plexus	Posterior dural plexus
Superficial venous system				
Superior sagittal group				
**v_frontopolar**	Frontopolar Vein	Anterior dural plexus	Plexus sagittalis	Superior Sagittal Sinus

**ant_frontal**	Anterior Frontal Vein	Anterior dural plexus	Plexus sagittalis	Superior Sagittal Sinus
**mid_frontal**	Middle Frontal Vein	Anterior dural plexus	Plexus sagittalis	Superior Sagittal Sinus
**post_frontal**	Posterior Frontal Vein	Anterior dural plexus	Plexus sagittalis	Superior Sagittal Sinus
**v_precentral**	Precentral Vein	Anterior dural plexus	Plexus sagittalis	Superior Sagittal Sinus
**central**	Central Vein	Anterior dural plexus	Plexus sagittalis	Superior Sagittal Sinus
**v_postcentral**	Postcentral Vein	Anterior dural plexus	Plexus sagittalis	Superior Sagittal Sinus
**ant_parietal**	Anterior Parietal Vein	Anterior dural plexus	Plexus sagittalis	Superior Sagittal Sinus
**post_parietal**	Posterior Parietal Vein	Anterior dural plexus	Plexus sagittalis	Superior Sagittal Sinus
**occipital**	Occipital Vein	Anterior dural plexus	Plexus sagittalis	Superior Sagittal Sinus
**trolard**	Vein of Trolard	Anterior dural plexus	Plexus sagittalis	Superior Sagittal Sinus
**anteromedial_frontal**	Anteromedial Frontal Vein	Anterior dural plexus	Plexus sagittalis	Superior Sagittal Sinus
**centromedial_frontal**	Centromedial Frontal Vein	Anterior dural plexus	Plexus sagittalis	Superior Sagittal Sinus
**posteromedial_frontal**	Posteromedial Frontal Vein	Anterior dural plexus	Plexus sagittalis	Superior Sagittal Sinus
**v_paracentral**	Paracentral Vein	Anterior dural plexus	Plexus sagittalis	Superior Sagittal Sinus
**anteromedial_parietal**	Anteromedial Parietal Vein	Anterior dural plexus	Plexus sagittalis	Superior Sagittal Sinus

**posteromedial_parietal**	Posteromedial Parietal Vein	Anterior dural plexus	Plexus sagittalis	Superior Sagittal Sinus
**post_calcarine**	Posterior Calcarine Vein	Anterior dural plexus	Plexus sagittalis	Superior Sagittal Sinus
Sphenoidal group				
**SMCV**	Superficial Middle Cerebral Vein	Primary head-vein	Cavernous sinus	Cavernous sinus
**frontosylvian**	Frontosylvian Veins	Primary head-vein	Cavernous sinus	Cavernous sinus
**parietosylvian**	Parietosylvian Veins	Primary head-vein	Cavernous sinus	Cavernous sinus
**temporosylvian**	Temporosylvian Veins	Primary head-vein	Cavernous sinus	Cavernous sinus
Tentorial group				
**ant_temporal**	Anterior Temporal Vein	Anterior dural plexus	Tentorial plexus	Tentorial plexus
**mid_temporal**	Middle Temporal Vein	Anterior dural plexus	Tentorial plexus	Tentorial plexus
**post_temporal**	Posterior Temporal Vein	Anterior dural plexus	Tentorial plexus	Tentorial plexus
**ant_temporobasal**	Anterior Temporobasal Vein	Anterior dural plexus	Tentorial plexus	Tentorial plexus
**mid_temporobasal**	Middle Temporobasal Vein	Anterior dural plexus	Tentorial plexus	Tentorial plexus
**post_temporobasal**	Posterior Temporobasal Vein	Anterior dural plexus	Tentorial plexus	Tentorial plexus
**occipital_basal**	Occipital Basal Vein	Anterior dural plexus	Tentorial plexus	Tentorial plexus
**labbe**	Vein of Labbé	Anterior dural plexus	Tentorial plexus	Tentorial plexus
Deep venous system				
Falcine group				
**paraterminal**	Paraterminal Vein	Anterior dural plexus	Inferior cerebral vein	Inferior cerebral vein
**post_frontoorbital**	Posterior Frontoorbital Vein	Anterior dural plexus	Inferior cerebral vein	Inferior cerebral vein
**olfactory**	Olfactory Vein	Anterior dural plexus	Inferior cerebral vein	Inferior cerebral vein
**ant_pericallosal**	Anterior Pericallosal Vein	Anterior dural plexus	Inferior cerebral vein	Inferior cerebral vein

**post_pericallosal**	Posterior Pericallosal Vein	Anterior dural plexus	Inferior cerebral vein	Inferior cerebral vein
**medial_temporal**	Medial Temporal Veins	Anterior dural plexus	Inferior cerebral vein	Inferior cerebral vein
**ant_calcarine**	Anterior Calcarine Vein	Anterior dural plexus	Inferior cerebral vein	Inferior cerebral vein
Internal cerebral vein				
**internal_cerebral**	Internal Cerebral Vein	NA	NA	Internal cerebral veins
**septal**	Septal Vein	NA	NA	Internal cerebral veins
**medial_atrial**	Medial Atrial Vein	NA	NA	Internal cerebral veins
**v_caudate**	Caudate Vein	NA	NA	Internal cerebral veins
**thalamostriate**	Thalamostriate Vein	NA	NA	Internal cerebral veins
**thalamocaudate**	Thalamocaudate Vein	NA	NA	Internal cerebral veins
**thalamic**	Thalamic Vein	NA	NA	Internal cerebral veins
Basal vein		NA	NA	Internal cerebral veins
**basal**	Basal Vein	NA	NA	Internal cerebral veins
**ant_cerebral**	Anterior Cerebral Vein	NA	NA	Internal cerebral veins
**DMCV**	Deep Middle Cerebral Vein	NA	NA	Internal cerebral veins
**inf_ventricular**	Inferior Ventricular Vein	NA	NA	Internal cerebral veins
**lat_atrial**	Lateral Atrial Vein	NA	NA	Internal cerebral veins

Posterior fossa veins				
**PCV**	Precentral Cerebellar Vein	Middle dural plexus	Tentorial plexus	Tentorial plexus
**SVV**	Superior Vermian Vein	Middle dural plexus	Tentorial plexus	Tentorial plexus
**APMV**	Anterior Pontomesencephalic Vein	Middle dural plexus	Tentorial plexus	Tentorial plexus
**petrosal**	Petrosal Vein	Middle dural plexus	Tentorial plexus	Tentorial plexus
**IVV**	Inferior Vermian Veins	Middle dural plexus	Tentorial plexus	Tentorial plexus
**hemispheric**	Hemispheric Cerebellar Veins	Middle dural plexus	Tentorial plexus	Tentorial plexus
**PMVP**	Perimedullary Venous Plexus	Middle dural plexus	Tentorial plexus	Tentorial plexus
**galen**	Vein of Galen	Anterior dural plexus	Tentorial plexus	Tentorial plexus
