# Supplementary material for: Anatomical phenotyping and staging of brain arteriovenous malformations
Source: Brain Commun. 2026 Feb 8;8(1):fcag039. doi: 10.1093/braincomms/fcag039 (PMC12917541; doi:10.1093/braincomms/fcag039)
Supplement: fcag039_Supplementary_Data [file fcag039_supplementary_data.zip › Supplementary Materials.pdf]

Supplementary Materials

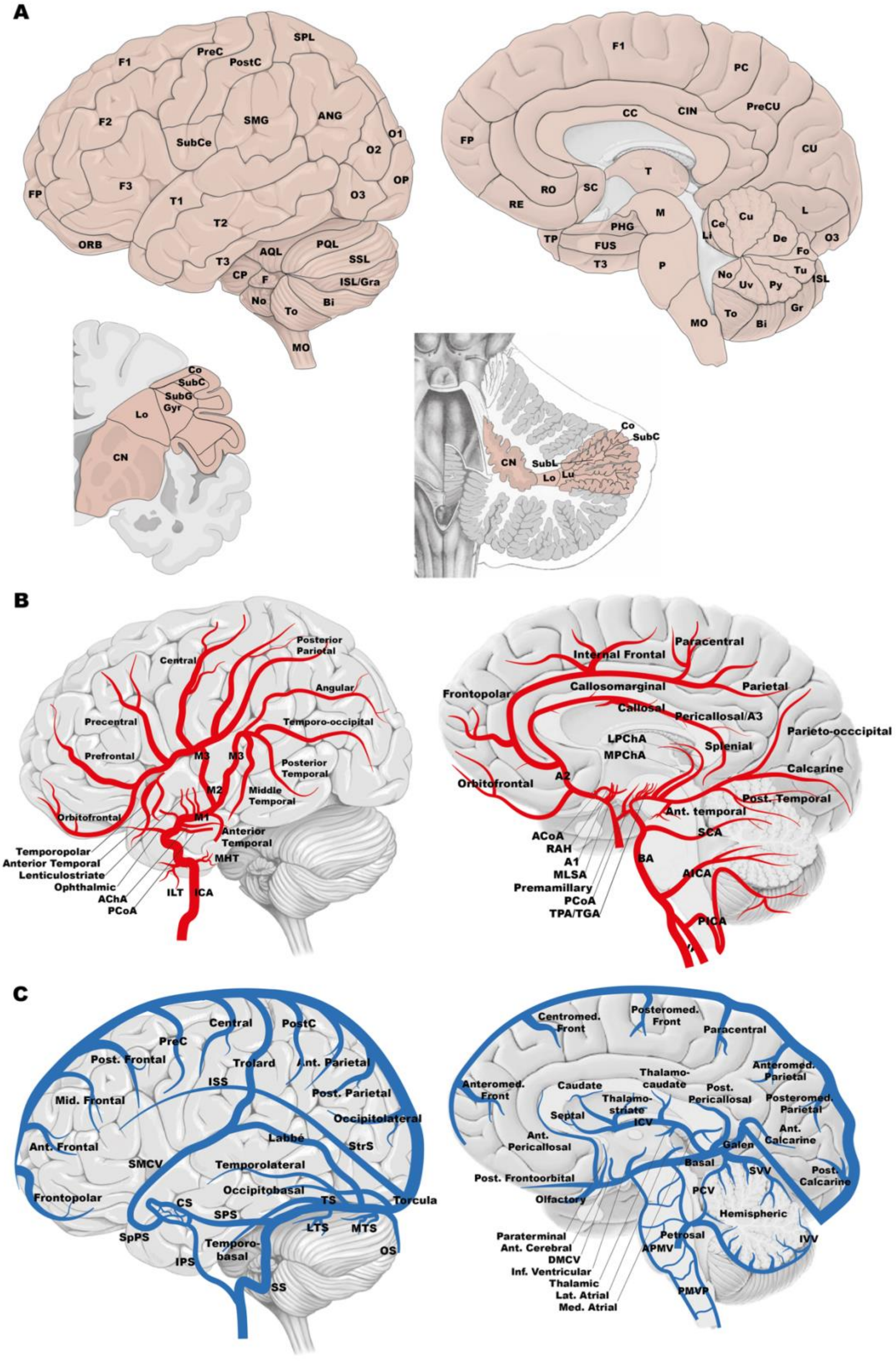

Supplementary Figure 1: Anatomical Topographic Reference Map. This figure provides a detailed anatomical reference for (A) gyral, (B) arterial and (C) venous structures. Abbreviations: A1-A3: A1-A3 segment of the anterior cerebral artery; ACoA: anterior communicating artery; AChA: anterior choroidal artery; AICA: anterior inferior cerebellar artery; ANG: angular gyrus; APMV: anterior pontomesencephalic venous plexus; AQL: anterior quadrangular lobule; BA: basilar artery; Bi: biventer/biventral lobule; CC: corpus callosum; Ce: central lobule; CIN: cingulate gyrus; CN: Cerebellar Nuclei; Co: Cortex; CP: cerebellar peduncle; CS: cavernous sinus; CU: cuneus; Cu: culmen; De: declive DMCV: deep middle cerebral vein; F1: superior frontal gyrus; F2: middle frontal gyrus; F3: inferior frontal gyrus; F: Flocculus; Fo: folium; FP: frontal pole; FUS: fusiform gyrus; Gr: Gracile lobule; Gyr: Gyral; ICA: internal carotid artery; ICV: internal cerebral vein; ILT: inferolateral trunk; IPS: inferior petrosal sinus; ISL: inferior semilunar lobule; ISS: inferior sagittal sinus; IVV: inferior vermian vein; L: lingual gyrus; Li: Lingula; LPChA: lateral posterior choroidal artery; Lo: Lobar; LTS: lateral tentorial sinus; Lu: Lobular; M1-M3: M1-M3 segment of the middle cerebral artery; MHT: meningohypophyseal trunk; MLSA: medial lenticulostriate arteries; MPChA: medial posterior choroidal artery; MTS: medial tentorial sinus; No: nodule; O1: superior occipital gyrus; O2: middle occipital gyrus; O3: inferior occipital gyrus; OP: occipital pole; ORB: orbital gyri; OS: occipital sinus; PC: paracentral lobule; PCV: precentral cerebellar vein; PCoA: posterior communicating artery; PHG: parahippocampal gyrus; PICA: posterior inferior cerebellar artery; PMVP: pontomedullary venous plexus; PostC: postcentral gyrus; PreC: precentral gyrus; PreCU: precuneus; PQL: posterior quadrangular lobule; Py: pyramid; RAH: recurrent artery of Heubner; RE: gyrus rectus; RO: rostral gyrus; SC: subcallosal area; SCA: superior cerebellar artery; SMCV: superior middle cerebral vein; SMG: supramarginal gyrus; SPL: superior parietal lobule; SpPS: sphenoparietal sinus; SPS: superior petrosal sinus; SS: sigmoid sinus; SSL: superior semilunar lobule; SSS: superior sagittal sinus; StrS: straight sinus; SubC: Subcortical; SubCe: subcentral gyrus; SubG: Subgyral; SubL: Sublobular; SVV: superior vermian vein; T1: superior temporal gyrus; T2: middle temporal gyrus; T3: inferior temporal gyrus; T: thalamus; To: tonsil; TP: temporal pole; TPA/TGA: thalamoperforating and thalamogeniculate arteries; TS: transverse sinus; Tu: tuber; Uv: uvula; VA: vertebral artery.

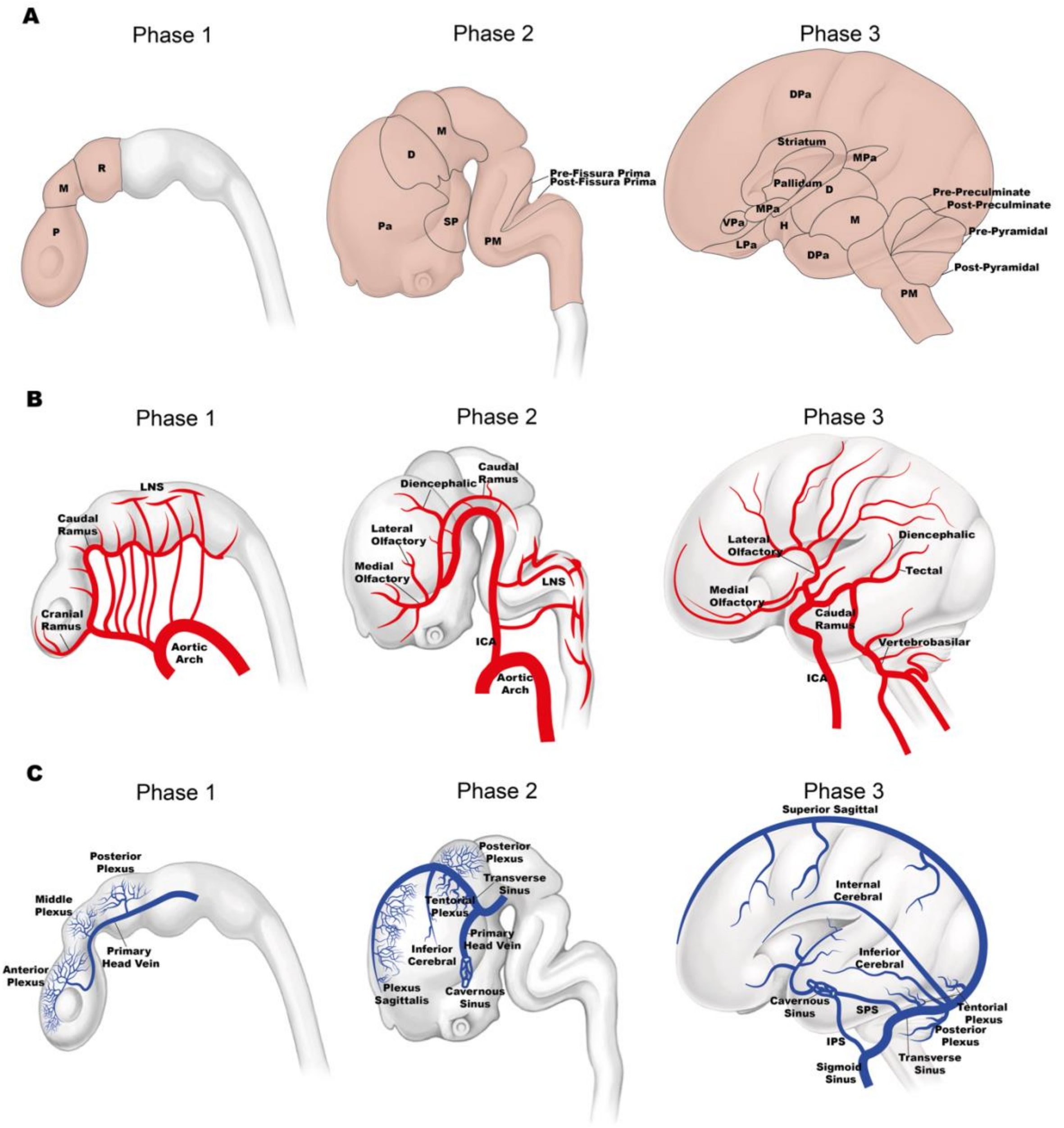

Supplementary Figure 2: Reference Map for Ontogenetic Units. Anatomical reference for ontogenetic mapping, illustrating (A) parenchymal, (B) arterial and (C) venous structures across developmental Phase 1 (Neural Tube Phase), Phase 2 (Vesicle Formation), and Phase 3 (Late Maturation Phase). Abbreviations: D = Diencephalon; DPa = Dorsal Pallium; H = Hypothalamus; ICA: Internal Carotid Artery; IPS: Inferior Petrosal Sinus; LNS: Longitudinal Neural System; LPa = Lateral Pallium; M = Mesencephalon; MPa = Medial Pallium; P = Prosencephalon; Pa = Pallium; PM = Pontomedullary Rhombecephalon; R = Rhombencephalon; SP = Subpallium; SPS: Superior Petrosal Sinus; VPa = Ventral Pallium.

Supplementary Table 1. Baseline Characteristics of the Pilot Cohort

Characteristic	N = 416[1]
**Age**	38.13 (18.59)
**Female Sex**	205 (49%)
**Clinical Presentation**	
Hemorrhage	149 (36%)
Seizure	98 (24%)
Other	54 (13%)
FND	15 (3.6%)
Incidental	100 (24%)
**Hemorrhage**	173 (41.6%)
SAH	22 (5.3%)
ICB	138 (333.2%)
SDH	4 (1.0%)
IVH	87 (20.9%)
unknown	10 (2.4%)
**Seizure**	126 (30.3%)
**Semiology**	
No seizure	289 (69.5%)
Focal to bilateral tonic-clonic	47 (11.3%)
Focal aware motor	29 (7.0%)
Focal aware nonmotor	26 (6.2%)
Unknown motor	1 (0.2%)
Unknown nonmotor	24 (5.8%)
**FND**	134 (32.2%)
**mRS at Diagnosis**	
0	145 (34.9%)
1	123 (29.6%)
2	53 (12.7%)
3	16 (3.8%)
4	35 (8.4%)
5	33 (7.9%)
unknown	11 (2.6%)
**mRS at Last Follow-Up**	
0	198 (47.6%)
1	97 (23.3%)
2	49 (11.8%)
3	34 (8.2%)
4	10 (2.4%)
6	22 (5.3%)
unknown	6 (1.4%)

Characteristic	N = 416[1]
**Mortality**	23 (5.5%)
**Nidus Volume**	12,035.71 (20,876.02)
**Aneurysms**	
Arterial Aneurysm	54 (13.0%)
Nidal aneurysm	60 (14.4%)
Venous Aneurysm	18 (4.3%)
**Arterial Dilation**	131 (31.5%)
**Venous Dilation**	240 (57.7%)
**Venous Ectasia**	86 (20.7%)
**Venous Stenosis**	55 (13.2%)
**Periventricular Drainage**	129 (31.0%)
**Spetzler-Martin Grade**	
1	81 (19.5%)
2	169 (40.6%)
3	110 (26%)
4	47 (11.3%)
5	9 (2.2%)
**Supplementary AVM Grade**	
1	31 (7.5%)
2	141 (33.9%)
3	143 (33.9%)
4	93 (22.4%)
5	8 (1.9%)
**Treatment**	
Conservative	73 (17.5%)
Treated	343 (82.5%)
**Surgery**	110 (26.4%)
**Embolization**	280 (67.3%)
**Radiosurgery**	11 (2.6%)

[1] Mean (SD); n (%)

Supplementary Table 2: Topographic Prevalence of Parenchymal Units

Parenchymal Unit	Count	Percentage (%)
**Frontal_Lobe**	76	18.27
Frontal Pole	7	1.68
F1	25	6.01
F2	25	6.01
F3	18	4.33
F3_Orbital	6	1.44
F3_Triangular	9	2.16
F3_Opercular	9	2.16
Orbital_Gyrus	13	3.12
Anterior_Orbital	7	1.68
Posterior_Orbital	8	1.92
Medial_Orbital	7	1.68
Lateral_Orbital	9	2.16
Rectus	8	1.92
Rostral	7	1.68
Subcallosal	9	2.16
**Central_Lobe**	57	13.70
Precentral	27	6.49
Postcentral	31	7.45
Paracentral	14	3.37
Subcentral	12	2.88
**Parietal_Lobe**	62	14.90
Superior_Parietal	21	5.05
Supramarginal	27	6.49
Angular	18	4.33
Precuneus	23	5.53
**Temporal_Lobe**	58	13.94

Parenchymal Unit	Count	Percentage (%)
T1	16	3.85
T2	23	5.53
T3	17	4.09
Planum_Polare	9	2.16
Planum_Temporale	11	2.64
Fusiform	6	1.44
**Insula**	18	4.33
Short_Insular	7	1.68
Long_Insular	12	2.88
**Occipital_Lobe**	28	6.73
O1	3	0.72
O2	8	1.92
O3	2	0.48
Occipital_Pole	6	1.44
Cuneus	14	3.37
Lingual	8	1.92
**Limbic_Lobe**	59	14.18
Cingulate	26	6.25
Anterior_Cingulate	7	1.68
Middle_Cingulate	7	1.68
Posterior_Cingulate	14	3.37
Parahippocampal	15	3.61
Hippocampus	12	2.88
Corpus_Callosum	26	6.25
**Basal_Ganglia**	37	8.89
Internal_Capsule	21	5.05
Caudate	12	2.88
Putamen	16	3.85

Parenchymal Unit	Count	Percentage (%)
Globus_Pallidus	13	3.12
**Diencephalon**	37	8.89
Hypothalamus	6	1.44
Thalamus	27	6.49
Amygdala	8	1.92
**Brainstem**	17	4.09
Mesencephalon	12	2.88
Pons	7	1.68
Medulla_Oblongata	1	0.24
**Cerebellum_Total**	61	14.66
Cerebellar_Peduncles	16	3.85
Central_Lobule	7	1.68
Ala_Lobuli_Centralis	6	1.44
Culmen	11	2.64
Anterior_Quadrangular	11	2.64
Declive	11	2.64
Folium	14	3.37
Post_Quadrangular	12	2.88
Superior_Semilunar	17	4.09
Inferior_Semilunar	15	3.61
Biventral_Lobule	14	3.37
Tuber	7	1.68
Pyramis	6	1.44
Uvula	5	1.20
Tonsil	6	1.44
Nodulus	5	1.20
Flocculus	5	1.20
Vermis	26	6.25

Parenchymal Unit	Count	Percentage (%)
Cerebellar_Nuclei	9	2.16
**Cerebellar White Matter**		
Cerebellar_Lobar_WM	17	4.09
Cerebellar_Lobular_WM	31	7.45
Cerebellar_Sublobular_WM	47	11.30
Cerebellar_Subcortical_WM	49	11.78
Cerebellar_Cortical_WM	50	12.02
**Cerebral White Matter**		
Cerebral_Central_WM	53	12.74
Cerebral_Lobar_WM	146	35.10
Cerebral_Gyral_WM	206	49.52
Cerebral_Subgyral_WM	244	58.65
Cerebral_Subcortical_WM	274	65.87
Cerebral_Cortical_WM	281	67.55

Supplementary Table 3: Topographic Prevalence of Arterial Units

Arterial Unit	Count	Percentage (%)
**Carotid**	66	15.87
C4	14	3.37
MHT	11	2.64
ILT	3	0.72
C6	7	1.68
Ophthalmic	7	1.68
C7	45	10.82
PCoA	5	1.20
Premamillary	3	0.72
AchA	44	10.58
**ACA**	172	41.35
A1	26	6.25
MLSA	14	3.37
ACoA	4	0.96
A2	34	8.17
A3	134	32.21
RAH	8	1.92
Pericallosal	71	17.07
Callosomarginal	72	17.31
Orbitofrontal	20	4.81
Frontopolar	17	4.09
IFA	41	9.86
Paracentral	36	8.65
Parietal	58	13.94
Callosal	16	3.85
**MCA**	252	60.58
M1	71	17.07

Arterial Unit	Count	Percentage (%)
M2	41	9.86
M3	22	5.29
M4	199	47.84
LLSA	57	13.70
MCA_ant_temporal	17	4.09
M4_orbitofrontal	20	4.81
M4_prefrontal	30	7.21
M4_precentral	27	6.49
M4_central	42	10.10
M4_ant_parietal	43	10.34
M4_post_parietal	38	9.13
M4_angular	44	10.58
M4_temporooccipital	32	7.69
M4_post_temporal	20	4.81
M4_med_temporal	25	6.01
M4_ant_temporal	16	3.85
M4_temporopolar	7	1.68
**PCA**	172	41.35
TPA	14	3.37
TGA	12	2.88
Peduncular	12	2.88
P2	61	14.66
P3	61	14.66
P4	78	18.75
MPChA	33	7.93
LPChA	61	14.66
Anterior_temporal	15	3.61
Posterior_temporal	15	3.61

Arterial Unit	Count	Percentage (%)
Calcarine	23	5.53
Parietooccipital	65	15.62
AITA	11	2.64
MITA	15	3.61
PITA	16	3.85
Splenial	18	4.33
**VA**	3	0.72
V3	0	0.00
V4	3	0.72
PICA	43	10.34
**BA**	10	2.40
AICA	28	6.73
SCA	59	14.18
**ECA**	71	17.07

Supplementary Table 4: Topographic Prevalence of Venous Units

Venous Unit	Count	Percentage (%)
**Sinuses**		
Superior Sagittal Sinus	238	57.21
Inferior Sagittal Sinus	8	1.92
Straight Sinus	4	0.96
Torcula	13	3.12
Transverse Sinus	101	24.28
Medial Tentorial Sinus	18	4.33
Lateral Tentorial Sinus	17	4.09
Sigmoid Sinus	23	5.53
Cavernous Sinus	18	4.33
Superior Petrosal Sinus	23	5.53
Inferior Petrosal Sinus	4	0.96
Sphenoparietal Sinus	29	6.97
**Superficial Drainage**		
Frontopolar	16	3.85
Anterior Frontal	28	6.73
Middle Frontal	31	7.45
Posterior Frontal	43	10.34
Precentral	35	8.41
Central	50	12.02
Postcentral	40	9.62
Anterior Parietal	47	11.30
Posterior Parietal	46	11.06
Occipital	23	5.53
Trolard	38	9.13
Anteromedial Frontal	5	1.20
Centromedial Frontal	7	1.68

Venous Unit	Count	Percentage (%)
Posteromedial Frontal	7	1.68
Paracentral	10	2.40
Anteromedial Parietal	13	3.12
Posteromedial Parietal	15	3.61
Posterior Calcarine	9	2.16
Superficial Middle Cerebral	45	10.82
Frontosylvian	16	3.85
Parietosylvian	3	0.72
Temporosylvian	25	6.01
Lateral Temporal	31	7.45
Anterior Temporal	11	2.64
Middle Temporal	16	3.85
Posterior Temporal	18	4.33
Temporobasal	25	6.01
Anterior Temporobasal	12	2.88
Middle Temporobasal	11	2.64
Posterior Temporobasal	10	2.40
Occipital Basal	13	3.12
Labbé	54	12.98
**Deep Drainage**		
Paraterminal	4	0.96
Posterior Frontoorbital	10	2.40
Olfactory	8	1.92
Anterior Pericallosal	4	0.96
Posterior Pericallosal	10	2.40
Medial Temporal	3	0.72
Anterior Calcarine	11	2.64
Internal Cerebral	89	21.39

Venous Unit	Count	Percentage (%)
Septal	17	4.09
Medial Atrial	32	7.69
Caudate	10	2.40
Thalamostriate	34	8.17
Thalamocaudate	14	3.37
Thalamic	11	2.64
Basal	80	19.23
Anterior Cerebral	14	3.37
Deep Middle Cerebral Vein	20	4.81
Inferior Ventricular	18	4.33
Lateral Atrial	27	6.49
Precentral Cerebellar	23	5.53
Superior Vermian	29	6.97
Anterior Pontomesencephalic	15	3.61
Pontomesencephalic	4	0.96
Petrosal	23	5.53
Hemispheric	36	8.65
Inferior Vermian	12	2.88
Galen	72	17.31

Supplementary Table 5: Topographic Probability of Parenchymal Units

Parenchymal Unit	Count	Relative Volume	Adjusted Count	Adjusted_Proportion
**Frontal Lobe**				
Frontal_Pole	7	0.43	16.25	0.04
F1_Total	25	6.28	3.98	0.01
F2_Total	25	5.34	4.68	0.01
F3_Orbital	6	0.36	16.76	0.04
F3_Triangular	9	1.07	8.40	0.02
F3_Opercular	9	1.28	7.04	0.02
ORB_Ant	7	0.31	22.89	0.06
ORB_Post	8	0.68	11.81	0.03
ORB_Med	7	0.69	10.13	0.02
ORB_Lat	9	0.55	16.26	0.04
Rectus	8	0.91	8.76	0.02
Rostral	7	0.33	21.25	0.05
Subcallosal	9	0.22	41.46	0.10
**Central Lobe**				
Precentral	27	3.59	7.53	0.02
Postcentral	31	2.04	15.21	0.04
Paracentral	14	1.29	10.88	0.03
Subcentral	12	0.55	21.68	0.05
**Parietal Lobe**				
SPL	21	3.05	6.88	0.02
SMG	27	2.81	9.62	0.02
ANG	18	3.14	5.73	0.01
Precuneus	23	2.82	8.16	0.02
**Temporal Lobe**				
T1_Total	16	2.49	6.43	0.02
T2_Total	23	2.17	10.59	0.03

Parenchymal Unit	Count	Relative Volume	Adjusted Count	Adjusted_Proportion
T3_Total	17	0.46	37.17	0.09
Planum_Polare	9	0.91	9.88	0.02
Planum_Temporale	11	0.28	39.21	0.09
Fusiform	6	2.03	2.95	0.01
**Insular Lobe**				
Insula_Short	7	1.41	4.95	0.01
Insula_Long	12	0.83	14.42	0.03
**Occipital Lobe**				
O1	3	0.84	3.59	0.01
O2	8	1.44	5.55	0.01
O3	2	0.85	2.36	0.01
Occipital_Pole	6	1.04	5.77	0.01
Cuneus	14	0.92	15.19	0.04
Lingual	8	1.86	4.30	0.01
**Limbic Lobe**				
Cin_Ant	7	1.32	5.31	0.01
Cin_Mid	7	1.56	4.48	0.01
Cin_Post	14	1.84	7.60	0.02
PHG	15	0.96	15.69	0.04
Hippocampus	12	0.74	16.11	0.04
Corpus_Callosum	26	0.30	86.66	0.21
**Basal Ganglia**				
Internal_Capsule	21	0.97	21.65	0.05
Caudate	12	0.71	16.81	0.04
Putamen	16	1.03	15.47	0.04
Globus_Pallidus	13	0.28	46.34	0.11
**Diencephalon**				

Parenchymal Unit	Count	Relative Volume	Adjusted Count	Adjusted_Proportion
Hypothalamus	6	0.73	8.23	0.02
Thalamus	27	1.34	20.15	0.05
Amygdala	8	0.29	27.93	0.07
**Brainstem**				
Mesencephalon	12	0.92	13.03	0.03
Pons	7	1.40	5.00	0.01
Medulla_Oblongata	1	0.25	3.96	0.01
**Cerebellum**				
Cerebellar_Peduncles	16	0.62	25.81	0.06
Central_Lobule	7	0.04	157.31	0.38
Ala_Lobuli_Centralis	6	1.10	5.43	0.01
Culmen	11	0.20	54.82	0.13
Ant_Quadrangular	11	1.62	6.78	0.02
Declive	11	0.09	116.33	0.28
Folium	14	0.04	345.53	0.83
Post_Quadrangular	12	1.20	9.98	0.02
Superior_Semilunar	17	1.30	13.03	0.03
Inferior_Semilunar	15	3.22	4.66	0.01
Biventral_Lobule	14	1.11	12.65	0.03
Tuber	7	0.04	196.99	0.47
Pyramis	6	0.04	153.90	0.37
Uvula	5	0.07	69.23	0.17
Tonsil	6	0.55	10.98	0.03
Nodulus	5	0.02	254.79	0.61
Flocculus	5	0.08	63.54	0.15

Supplementary Table 6: Ontogenetic Mapping of Parenchymal Structures

Ontogenetic Unit	N (%)
**Phase 1**	
Prosencephalon	664(77.93%)
Mesencephalon	176(20.66%)
Rhombencephalon	12(1.41%)
**Phase 2**	
Pallium	590(69.25%)
Pre-Fissura Prima	51(5.99%)
Post-Fissura Prima	117(13.73%)
Subpallium	47(5.52%)
Diencephalon	27(3.17%)
Mesencephalon	12(1.41%)
Pontomedullary Rhombecephalon	8(0.94%)
**Phase 3**	
Dorsal Pallium	462(54.23%)
Medial Pallium	96(11.27%)
Pre-prepyramidal Fissure	76(8.92%)
Lateral Pallium	24(2.82%)
Post-prepyramidal Fissure	52(6.10%)
Pre-preculminate Fissure	29(3.40%)
Striatum	28(3.29%)
Diencephalon	27(3.17%)
Mesencephalon	12(1.41%)
Post-preculminate Fissure	11(1.29%)
Pallidum	13(1.53%)
Hypothalamus	6(0.70%)
Pontomedullary Rhombecephalon	8(0.94%)
Ventral Pallium	8(0.94%)

Supplementary Table 7: Ontogenetic Mapping of Arterial Structures

Ontogenetic Unit	N (%)
**Phase 1**	
Cranial Ramus	1119(63.98%)
Caudal Ramus	425(24.30%)
Internal Carotid	153(8.75%)
Longitudinal Neural System	52(2.97%)
**Phase 2**	
Lateral Olfactory	741(42.37%)
Medial Olfactory	378(21.61%)
Diencephalic	260(14.87%)
Internal Carotid	153(8.75%)
Caudal Ramus	165(9.43%)
Longitudinal Neural System	52(2.97%)
**Phase 3**	
Lateral Striate	741(42.37%)
Medial Olfactory	378(21.61%)
Tectal	173(9.89%)
Internal Carotid	153(8.75%)
Diencephalic	147(8.40%)
Caudal Ramus	105(6.00%)
Vertebrobasilar	52(2.97%)

Supplementary Table 8: Ontogenetic Mapping of Venous Structures

Ontogenetic Unit	N (%)
**Phase 1**	
Anterior Dural Plexus	1021(51.46%)
Middle Dural Plexus	310(15.62%)
Posterior Dural Plexus	124(6.25%)
Primary Head-Vein	163(8.22%)
**Phase 2**	
Plexus Sagittalis	706(35.58%)
Tentorial Plexus	416(20.97%)
Transverse Sinus	124(6.25%)
Cavernous Sinus	136(6.85%)
Inferior Cerebral Vein	50(2.52%)
Primary Head-Vein	27(1.36%)
Posterior Dural Plexus	0(0.00%)
**Phase 3**	
Superior Sagittal Sinus	702(35.38%)
Internal Cerebral Veins	370(18.65%)
Tentorial Plexus	416(20.97%)
Transverse Sinus	101(5.09%)
Cavernous Sinus	136(6.85%)
Inferior Cerebral Vein	50(2.52%)
Sigmoid Sinus	23(1.16%)
Superior Petrosal Sinus	23(1.16%)
Inferior Petrosal Sinus	4(0.20%)
Posterior Dural Plexus	0(0.00%)

Supplementary Table 9: Meta-topology (1-3) Characterization

Feature	Meta Topology 1	Meta Topology 2	Meta Topology 3
**PARENCHYMA**			
**Frontal Lobe**			
frontal_pole	1 (1.56%)	6 (11.11%)	–
F1total	–	3 (5.56%)	22 (26.83%)
F2total	–	13 (24.07%)	12 (14.63%)
F3total	–	18 (33.33%)	–
F3orbital	–	6 (11.11%)	–
F3triangular	–	9 (16.67%)	–
F3opercular	–	9 (16.67%)	–
orbital_gyrus	–	13 (24.07%)	–
ORBant	–	7 (12.96%)	–
ORBpost	–	8 (14.81%)	–
ORBmed	–	7 (12.96%)	–
ORBlat	–	9 (16.67%)	–
rectus	–	8 (14.81%)	–
rostral	–	7 (12.96%)	–
subcallosal	–	8 (14.81%)	–
**Central Lobe**			
precentral	–	–	26 (31.71%)
postcentral	2 (3.12%)	–	26 (31.71%)
paracentral	–	–	13 (15.85%)
subcentral	1 (1.56%)	3 (5.56%)	6 (7.32%)
**Parietal Lobe**			
SPL	–	1 (1.85%)	1 (1.22%)
SMG	3 (4.69%)	2 (3.7%)	3 (3.66%)
ANG	2 (3.12%)	1 (1.85%)	–
precuneus	–	–	1 (1.22%)

Feature	Meta Topology 1	Meta Topology 2	Meta Topology 3
**Temporal Lobe**			
temporal pole	6 (9.38%)	–	–
T1total	15 (23.44%)	1 (1.85%)	–
T2total	20 (31.25%)	1 (1.85%)	–
T3total	15 (23.44%)	–	–
planum polare	7 (10.94%)	1 (1.85%)	–
planum temporale	8 (12.5%)	1 (1.85%)	–
fusiform	6 (9.38%)	–	–
**Insular Lobe**	(3.12%)	(24.07%)	(1.22%)
INSshort	–	6 (11.11%)	–
INSlong	2 (3.12%)	8 (14.81%)	1 (1.22%)
**Occipital Lobe**			
O1	–	–	–
O2	–	–	–
O3	1 (1.56%)	–	–
occipital pole	–	–	–
cuneus	–	–	–
lingual	–	–	–
**Limbic Lobe**			
cingulate	–	1 (1.85%)	9 (10.98%)
CINant	–	1 (1.85%)	6 (7.32%)
CINmid	–	–	3 (3.66%)
CINpost	–	–	1 (1.22%)
PHG	9 (14.06%)	1 (1.85%)	–
Hippocampus	7 (10.94%)	1 (1.85%)	–
corpus callosum	–	2 (3.7%)	4 (4.88%)
**Basal Ganglia**			
internal capsule	–	2 (3.7%)	–

Feature	Meta Topology 1	Meta Topology 2	Meta Topology 3
caudate	–	3 (5.56%)	–
putamen	–	2 (3.7%)	–
globus pallidus	–	2 (3.7%)	–
**Diencephalon**			
hypothalamus	–	3 (5.56%)	–
thalamus	–	–	–
amygdala	5 (7.81%)	1 (1.85%)	–
**Brainstem**			
mesencephalon	–	–	–
pons	–	–	–
medulla oblongata	–	–	–
**Cerebellum**			
cerebellar peduncles	–	–	–
cerebellar hemisphere	–	–	–
central lobule	–	–	–
ala lobuli centralis	–	–	–
culmen	–	–	–
anterior quadrangular	–	–	–
declive	–	–	–
folium	–	–	–
post quadrangular	–	–	–
superior semilunar	–	–	–
inferior semilunar	–	–	–
biventral lobule	–	–	–
tuber	–	–	–
pyramis	–	–	–
uvula	–	–	–
tonsil	–	–	–

Feature	Meta Topology 1	Meta Topology 2	Meta Topology 3
nodulus	–	–	–
flocculus	–	–	–
vermis	–	–	–
cerebellar nuclei	–	–	–
**White Matter**			
cerebellar cortex	–	–	1 (1.22%)
cerebellar lobar WM sector	–	–	–
cerebellar lobular WM sector	–	–	–
cerebellar sublobular WM sector	–	–	1 (1.22%)
cerebellar subcortical WM sector	–	–	1 (1.22%)
cerebral cortex	63 (98.44%)	52 (96.3%)	78 (95.12%)
cerebral central WM sector	1 (1.56%)	8 (14.81%)	3 (3.66%)
cerebral lobar WM sector	18 (28.12%)	28 (51.85%)	38 (46.34%)
cerebral gyral WM sector	43 (67.19%)	40 (74.07%)	54 (65.85%)
cerebral subgyral WM sector	54 (84.38%)	46 (85.19%)	64 (78.05%)
cerebral subcortical WM sector	61 (95.31%)	50 (92.59%)	75 (91.46%)
**ARTERIES**			
**carotid**	21 (32.81%)	8 (14.81%)	2 (2.44%)
MHT	3 (4.69%)	–	1 (1.22%)
ILT	2 (3.12%)	–	–
ophthalmic	1 (1.56%)	5 (9.26%)	1 (1.22%)
SHA	–	–	–
PCoA	–	–	–
premamillary	–	–	–
AchA	16 (25%)	2 (3.7%)	1 (1.22%)
**ACA**			
MLSA	–	3 (5.56%)	3 (3.66%)
ACoA	–	3 (5.56%)	–

Feature	Meta Topology 1	Meta Topology 2	Meta Topology 3
RAH	–	3 (5.56%)	1 (1.22%)
callosomarginal	–	2 (3.7%)	48 (58.54%)
orbitofrontal	–	20 (37.04%)	–
ACA frontopolar	–	15 (27.78%)	2 (2.44%)
IFA	–	4 (7.41%)	36 (43.9%)
ACA paracentral	–	1 (1.85%)	31 (37.8%)
parietal	–	1 (1.85%)	18 (21.95%)
callosal	–	1 (1.85%)	3 (3.66%)
**MCA**			
M2	11 (17.19%)	18 (33.33%)	3 (3.66%)
M3	11 (17.19%)	8 (14.81%)	2 (2.44%)
LLSA	3 (4.69%)	12 (22.22%)	20 (24.39%)
MCA ant temporal	16 (25%)	1 (1.85%)	–
M4 orbitofrontal	–	19 (35.19%)	1 (1.22%)
M4 prefrontal	–	17 (31.48%)	11 (13.41%)
M4 precentral	1 (1.56%)	2 (3.7%)	23 (28.05%)
M4 central	2 (3.12%)	2 (3.7%)	37 (45.12%)
M4 ant parietal	1 (1.56%)	3 (5.56%)	29 (35.37%)
M4 post parietal	2 (3.12%)	2 (3.7%)	4 (4.88%)
M4 angular	3 (4.69%)	1 (1.85%)	1 (1.22%)
M4 temporooccipital	4 (6.25%)	–	–
M4 post temporal	18 (28.12%)	–	–
M4 med temporal	25 (39.06%)	–	–
M4 ant temporal	16 (25%)	–	–
M4 temporopolar	7 (10.94%)	–	–
**PCA**			
TPA	–	–	–
TGA	–	–	–

Feature	Meta Topology 1	Meta Topology 2	Meta Topology 3
PCA peduncular	–	–	–
MPChA	3 (4.69%)	3 (5.56%)	2 (2.44%)
LPChA	4 (6.25%)	4 (7.41%)	5 (6.1%)
PCA ant temporal	15 (23.44%)	–	–
PCA post temporal	13 (20.31%)	–	–
calcarine	1 (1.56%)	–	1 (1.22%)
parietooccipital	4 (6.25%)	1 (1.85%)	5 (6.1%)
AITA	11 (17.19%)	–	–
MITA	15 (23.44%)	–	–
PITA	14 (21.88%)	–	–
splenial	1 (1.56%)	1 (1.85%)	2 (2.44%)
**Vertebrobasilar**			
VA	–	–	–
PICA	–	–	–
BA	–	–	–
AICA	–	–	–
SCA	–	–	–
**ECA**	21 (32.81%)	6 (11.11%)	16 (19.51%)
**VEINS**			
**Sinuses**			
SSS	31 (48.44%)	46 (85.19%)	80 (97.56%)
ISS	–	–	2 (2.44%)
StrS	–	–	–
torcula	–	–	–
TS	24 (37.5%)	20 (37.04%)	12 (14.63%)
MTS	2 (3.12%)	–	–
LTS	8 (12.5%)	2 (3.7%)	–
SS	12 (18.75%)	3 (5.56%)	3 (3.66%)

Feature	Meta Topology 1	Meta Topology 2	Meta Topology 3
CS	5 (7.81%)	8 (14.81%)	1 (1.22%)
SPS	4 (6.25%)	4 (7.41%)	–
IPS	2 (3.12%)	–	–
SpPS	15 (23.44%)	9 (16.67%)	1 (1.22%)
OS	–	–	–
**Superficial Drainage**			
v frontopolar	2 (3.12%)	12 (22.22%)	–
ant frontal	9 (14.06%)	15 (27.78%)	3 (3.66%)
mid frontal	3 (4.69%)	15 (27.78%)	8 (9.76%)
post frontal	10 (15.62%)	7 (12.96%)	23 (28.05%)
v precentral	7 (10.94%)	5 (9.26%)	21 (25.61%)
central	6 (9.38%)	7 (12.96%)	31 (37.8%)
v postcentral	4 (6.25%)	5 (9.26%)	23 (28.05%)
ant parietal	9 (14.06%)	5 (9.26%)	12 (14.63%)
post parietal	7 (10.94%)	1 (1.85%)	5 (6.1%)
occipital	6 (9.38%)	–	2 (2.44%)
trolard	12 (18.75%)	10 (18.52%)	10 (12.2%)
anteromedial frontal	–	4 (7.41%)	1 (1.22%)
centromedial frontal	–	–	6 (7.32%)
posteromedial front	–	–	5 (6.1%)
v paracentral	–	–	5 (6.1%)
anteromedial parietal	–	–	3 (3.66%)
posteromedial parietal	–	–	–
post calcarine	–	–	–
SMCV	18 (28.12%)	21 (38.89%)	2 (2.44%)
frontosylvian	–	15 (27.78%)	1 (1.22%)
parietosylvian	–	2 (3.7%)	–
temporosylvian	18 (28.12%)	3 (5.56%)	1 (1.22%)

Feature	Meta Topology 1	Meta Topology 2	Meta Topology 3
ant temporal	5 (7.81%)	3 (5.56%)	3 (3.66%)
mid temporal	13 (20.31%)	1 (1.85%)	1 (1.22%)
post temporal	12 (18.75%)	–	–
ant temporobasal	7 (10.94%)	5 (9.26%)	–
mid temporobasal	9 (14.06%)	1 (1.85%)	–
post temporobasal	8 (12.5%)	–	–
occipital basal	1 (1.56%)	–	–
labbe	17 (26.56%)	17 (31.48%)	13 (15.85%)
**Deep Drainage**			
paraterminal	–	4 (7.41%)	–
post frontoorbital	–	10 (18.52%)	–
olfactory	–	8 (14.81%)	–
ant pericallosal	–	2 (3.7%)	–
post pericallosal	–	–	1 (1.22%)
medial temporal	2 (3.12%)	–	–
ant calcarine	–	–	–
internal cerebral	1 (1.56%)	14 (25.93%)	21 (25.61%)
septal	–	5 (9.26%)	6 (7.32%)
medial atrial	1 (1.56%)	1 (1.85%)	2 (2.44%)
v caudate	1 (1.56%)	–	6 (7.32%)
thalamostriate	1 (1.56%)	6 (11.11%)	9 (10.98%)
thalamocaudate	1 (1.56%)	1 (1.85%)	3 (3.66%)
thalamic	–	–	–
basal	17 (26.56%)	27 (50%)	1 (1.22%)
ant cerebral	–	12 (22.22%)	–
DMCV	6 (9.38%)	9 (16.67%)	–
inf ventricular	11 (17.19%)	–	–
lat atrial	5 (7.81%)	1 (1.85%)	1 (1.22%)

Feature	Meta Topology 1	Meta Topology 2	Meta Topology 3
PCV	–	–	1 (1.22%)
SVV	–	–	–
APMV	1 (1.56%)	–	–
PMVP	1 (1.56%)	–	–
petrosal	1 (1.56%)	–	–
hemispheric	–	–	–
IVV	–	–	–
galen	2 (3.12%)	–	4 (4.88%)

Supplementary Table 10: Meta-topology (4-6) Characterization

Feature	Meta Topology 4	Meta Topology 5	Meta Topology 6
**PARENCHYMA**			
**Frontal Lobe**			
frontal_pole	–	–	–
F1total	–	–	–
F2total	–	–	–
F3total	–	–	–
F3orbital	–	–	–
F3triangular	–	–	–
F3opercular	–	–	–
orbital_gyrus	–	–	–
ORBant	–	–	–
ORBpost	–	–	–
ORBmed	–	–	–
ORBlat	–	–	–
rectus	–	–	–
rostral	–	–	–
subcallosal	1 (1.67%)	–	–
**Central Lobe**			
precentral	–	–	1 (1.22%)
postcentral	–	–	3 (3.66%)
paracentral	–	–	1 (1.22%)
subcentral	–	–	2 (2.44%)
**Parietal Lobe**			
SPL	1 (1.67%)	–	18 (21.95%)
SMG	1 (1.67%)	–	18 (21.95%)
ANG	2 (3.33%)	–	13 (15.85%)
precuneus	1 (1.67%)	–	21 (25.61%)

Feature	Meta Topology 4	Meta Topology 5	Meta Topology 6
**Temporal Lobe**			
temporal pole	–	–	1 (1.22%)
T1total	–	–	–
T2total	1 (1.67%)	–	1 (1.22%)
T3total	1 (1.67%)	–	1 (1.22%)
planum polare	1 (1.67%)	–	–
planum temporale	1 (1.67%)	–	1 (1.22%)
fusiform	–	–	–
**Insular Lobe**	(3.33%)		
INSshort	1 (1.67%)	–	–
INSlong	1 (1.67%)	–	–
**Occipital Lobe**			
O1	1 (1.67%)	–	2 (2.44%)
O2	1 (1.67%)	–	7 (8.54%)
O3	1 (1.67%)	–	–
occipital pole	1 (1.67%)	–	5 (6.1%)
cuneus	1 (1.67%)	–	13 (15.85%)
lingual	–	–	8 (9.76%)
**Limbic Lobe**			
cingulate	4 (6.67%)	–	12 (14.63%)
CINant	–	–	–
CINmid	3 (5%)	–	1 (1.22%)
CINpost	1 (1.67%)	–	12 (14.63%)
PHG	3 (5%)	–	2 (2.44%)
Hippocampus	3 (5%)	–	1 (1.22%)
corpus callosum	17 (28.33%)	–	3 (3.66%)
**Basal Ganglia**			
internal capsule	19 (31.67%)	–	–

Feature	Meta Topology 4	Meta Topology 5	Meta Topology 6
caudate	9 (15%)	–	–
putamen	14 (23.33%)	–	–
globus pallidus	11 (18.33%)	–	–
**Diencephalon**			
hypothalamus	3 (5%)	–	–
thalamus	25 (41.67%)	2 (2.7%)	–
amygdala	2 (3.33%)	–	–
**Brainstem**			
mesencephalon	2 (3.33%)	10 (13.51%)	–
pons	1 (1.67%)	6 (8.11%)	–
medulla oblongata	–	1 (1.35%)	–
**Cerebellum**			
cerebellar peduncles	1 (1.67%)	15 (20.27%)	–
cerebellar hemisphere	–	40 (54.05%)	–
central lobule	–	7 (9.46%)	–
ala lobuli centralis	–	6 (8.11%)	–
culmen	–	11 (14.86%)	–
anterior quadrangular	–	11 (14.86%)	–
declive	–	11 (14.86%)	–
folium	–	14 (18.92%)	–
post quadrangular	–	12 (16.22%)	–
superior semilunar	–	17 (22.97%)	–
inferior semilunar	–	15 (20.27%)	–
biventral lobule	–	14 (18.92%)	–
tuber	–	7 (9.46%)	–
pyramis	–	6 (8.11%)	–
uvula	–	5 (6.76%)	–
tonsil	–	6 (8.11%)	–

Feature	Meta Topology 4	Meta Topology 5	Meta Topology 6
nodulus	–	5 (6.76%)	–
flocculus	–	5 (6.76%)	–
vermis	–	26 (35.14%)	–
cerebellar nuclei	–	9 (12.16%)	–
**White Matter**			
cerebellar cortex	–	49 (66.22%)	–
cerebellar lobar WM sector	1 (1.67%)	16 (21.62%)	–
cerebellar lobular WM sector	1 (1.67%)	30 (40.54%)	–
cerebellar sublobular WM sector	1 (1.67%)	45 (60.81%)	–
cerebellar subcortical WM sector	1 (1.67%)	47 (63.51%)	–
cerebral cortex	8 (13.33%)	1 (1.35%)	79 (96.34%)
cerebral central WM sector	38 (63.33%)	–	3 (3.66%)
cerebral lobar WM sector	20 (33.33%)	–	42 (51.22%)
cerebral gyral WM sector	7 (11.67%)	1 (1.35%)	61 (74.39%)
cerebral subgyral WM sector	7 (11.67%)	1 (1.35%)	72 (87.8%)
cerebral subcortical WM sector	8 (13.33%)	1 (1.35%)	79 (96.34%)
**ARTERIES**			
**carotid**	27 (45%)	4 (5.41%)	4 (4.88%)
MHT	2 (3.33%)	3 (4.05%)	2 (2.44%)
ILT	–	1 (1.35%)	–
ophthalmic	–	–	–
SHA	–	–	–
PCoA	5 (8.33%)	–	–
premamillary	3 (5%)	–	–
AchA	23 (38.33%)	–	2 (2.44%)
**ACA**			
MLSA	8 (13.33%)	–	–
ACoA	1 (1.67%)	–	–

Feature	Meta Topology 4	Meta Topology 5	Meta Topology 6
RAH	4 (6.67%)	–	–
callosomarginal	4 (6.67%)	–	18 (21.95%)
orbitofrontal	–	–	–
ACA frontopolar	–	–	–
IFA	1 (1.67%)	–	–
ACA paracentral	1 (1.67%)	–	3 (3.66%)
parietal	1 (1.67%)	–	38 (46.34%)
callosal	12 (20%)	–	–
**MCA**			
M2	7 (11.67%)	–	2 (2.44%)
M3	1 (1.67%)	–	–
LLSA	18 (30%)	–	4 (4.88%)
MCA ant temporal	–	–	–
M4 orbitofrontal	–	–	–
M4 prefrontal	2 (3.33%)	–	–
M4 precentral	1 (1.67%)	–	–
M4 central	–	–	1 (1.22%)
M4 ant parietal	1 (1.67%)	–	9 (10.98%)
M4 post parietal	2 (3.33%)	–	28 (34.15%)
M4 angular	4 (6.67%)	–	35 (42.68%)
M4 temporooccipital	3 (5%)	–	25 (30.49%)
M4 post temporal	1 (1.67%)	–	1 (1.22%)
M4 med temporal	–	–	–
M4 ant temporal	–	–	–
M4 temporopolar	–	–	–
**PCA**			
TPA	13 (21.67%)	1 (1.35%)	–
TGA	11 (18.33%)	1 (1.35%)	–

Feature	Meta Topology 4	Meta Topology 5	Meta Topology 6
PCA peduncular	2 (3.33%)	10 (13.51%)	–
MPChA	18 (30%)	–	7 (8.54%)
LPChA	33 (55%)	–	15 (18.29%)
PCA ant temporal	–	–	–
PCA post temporal	1 (1.67%)	–	1 (1.22%)
calcarine	2 (3.33%)	–	19 (23.17%)
parietooccipital	3 (5%)	–	52 (63.41%)
AITA	–	–	–
MITA	–	–	–
PITA	1 (1.67%)	–	1 (1.22%)
splenial	9 (15%)	–	5 (6.1%)
**Vertebrobasilar**			
VA	1 (1.67%)	2 (2.7%)	–
PICA	–	43 (58.11%)	–
BA	1 (1.67%)	9 (12.16%)	–
AICA	–	28 (37.84%)	–
SCA	1 (1.67%)	58 (78.38%)	–
**ECA**	2 (3.33%)	6 (8.11%)	20 (24.39%)
**VEINS**			
**Sinuses**			
SSS	11 (18.33%)	1 (1.35%)	69 (84.15%)
ISS	5 (8.33%)	–	1 (1.22%)
StrS	–	3 (4.05%)	1 (1.22%)
torcula	–	13 (17.57%)	–
TS	2 (3.33%)	22 (29.73%)	21 (25.61%)
MTS	2 (3.33%)	10 (13.51%)	4 (4.88%)
LTS	–	3 (4.05%)	4 (4.88%)
SS	2 (3.33%)	1 (1.35%)	2 (2.44%)

Feature	Meta Topology 4	Meta Topology 5	Meta Topology 6
CS	2 (3.33%)	2 (2.7%)	–
SPS	1 (1.67%)	14 (18.92%)	–
IPS	–	2 (2.7%)	–
SpPS	4 (6.67%)	–	–
OS	–	–	–
**Superficial Drainage**			
v frontopolar	2 (3.33%)	–	–
ant frontal	1 (1.67%)	–	–
mid frontal	3 (5%)	–	2 (2.44%)
post frontal	1 (1.67%)	–	2 (2.44%)
v precentral	1 (1.67%)	–	1 (1.22%)
central	1 (1.67%)	–	5 (6.1%)
v postcentral	1 (1.67%)	–	7 (8.54%)
ant parietal	1 (1.67%)	–	20 (24.39%)
post parietal	2 (3.33%)	–	31 (37.8%)
occipital	–	–	15 (18.29%)
trolard	–	–	6 (7.32%)
anteromedial frontal	–	–	–
centromedial frontal	1 (1.67%)	–	–
posteromedial front	2 (3.33%)	–	–
v paracentral	5 (8.33%)	–	–
anteromedial parietal	3 (5%)	–	7 (8.54%)
posteromedial parietal	3 (5%)	–	12 (14.63%)
post calcarine	1 (1.67%)	–	8 (9.76%)
SMCV	2 (3.33%)	–	2 (2.44%)
frontosylvian	–	–	–
parietosylvian	–	–	1 (1.22%)
temporosylvian	2 (3.33%)	–	1 (1.22%)

Feature	Meta Topology 4	Meta Topology 5	Meta Topology 6
ant_temporal	–	–	–
mid_temporal	–	–	1 (1.22%)
post_temporal	–	–	6 (7.32%)
ant_temporobasal	–	–	–
mid_temporobasal	–	–	1 (1.22%)
post_temporobasal	–	–	2 (2.44%)
occipital_basal	–	–	12 (14.63%)
labbe	1 (1.67%)	–	6 (7.32%)
**Deep Drainage**			
paraterminal	–	–	–
post_frontoorbital	–	–	–
olfactory	–	–	–
ant_pericallosal	2 (3.33%)	–	–
post_pericallosal	7 (11.67%)	–	2 (2.44%)
medial_temporal	1 (1.67%)	–	–
ant_calcarine	–	–	11 (13.41%)
internal_cerebral	37 (61.67%)	–	16 (19.51%)
septal	6 (10%)	–	–
medial_atrial	10 (16.67%)	–	18 (21.95%)
v_caudate	3 (5%)	–	–
thalamostriate	17 (28.33%)	–	1 (1.22%)
thalamocaudate	8 (13.33%)	–	1 (1.22%)
thalamic	11 (18.33%)	–	–
basal	20 (33.33%)	5 (6.76%)	10 (12.2%)
ant_cerebral	2 (3.33%)	–	–
DMCV	4 (6.67%)	–	1 (1.22%)
inf_ventricular	7 (11.67%)	–	–
lat_atrial	13 (21.67%)	–	7 (8.54%)

Feature	Meta Topology 4	Meta Topology 5	Meta Topology 6
PCV	1 (1.67%)	21 (28.38%)	–
SVV	–	29 (39.19%)	–
APMV	1 (1.67%)	13 (17.57%)	–
PMVP	–	3 (4.05%)	–
petrosal	–	22 (29.73%)	–
hemispheric	–	36 (48.65%)	–
IVV	–	12 (16.22%)	–
galen	11 (18.33%)	37 (50%)	18 (21.95%)
